# Bridging psychiatry and rare genetic diseases: a scoping review of therapeutic strategies and diagnostic delay paired with healthcare economic burden analysis

**DOI:** 10.1186/s13023-025-03941-8

**Published:** 2025-08-01

**Authors:** Sheldon R. Garrison, Isaac J. Siegel, Christopher R. Takala, Sarah L. Vaithilingam, Gene W. Yang, Anthony W. Zoghbi, Madeline M. Hartig, Sreya Vadapalli, Margaret E. Anderson

**Affiliations:** 1 Rogers Behavioral Health, Research Center, Ladish Building, 34700 Valley Road, Oconomowoc, WI 53066 USA; 2 Milwaukee Area Technical College, Milwaukee, WI 53233 USA; 3 Rogers Behavioral Health, Oconomowoc, WI 53066 USA; 4 Rogers Behavioral Health, Brown Deer, WI 53223 USA; 5 Rogers Behavioral Health, Walnut Creek, CA 94598 USA; 6 Department of Psychiatry, University of Wisconsin School of Medicine and Public Health, Madison, WI 53726 USA; 7 Menninger Department of Psychiatry and Behavioral Sciences, Baylor College of Medicine, Houston, TX 77030 USA

**Keywords:** Mental health, Addiction, Behavioral health, Psychiatric, Rare disease, Orphan drug, Medication, Diagnostic delay, Treatment-resistant

## Abstract

Rare genetic diseases (RDs) with primary neuropsychiatric symptoms pose unique challenges for diagnosis and management. While the majority of these RDs have neuropsychiatric symptoms that are secondary to the RD, a subset presents with primary neuropsychiatric symptoms directly linked to their underlying pathophysiology. This subset has significant unmet medical need with delayed diagnoses leading to prolonged delays in treatment optimization and the trialing of medications that fail to target the underlying pathophysiology. This comprehensive review identifies 108 RDs with central neuropsychiatric symptoms that have a 7.7-year average diagnostic delay. Optimal management strategies for these RDs typically includes non-psychotropic medications, dietary adjustment, avoidance of certain drug classes and other treatments that target the underlying pathophysiology before improvement of the neuropsychiatric symptoms is observed. Surprisingly, despite the limited number of RDs that fit this unique profile, the annual economic burden for these conditions had an annual mental health care-related inpatient charges totaling $4.2 billion USD. Addressing the diagnostic delay and optimizing management for these specific conditions must include increased involvement across multiple specialties, including psychiatry, family medicine, pediatrics, medical genetics, as well as an enhanced strategy for genetic testing to ensure the prompt initiation of condition-specific therapies for affected individuals.

**Supplementary Information:**

The online version contains supplementary material available at 10.1186/s13023-025-03941-8.

## Background

Rare diseases (RDs), affecting an estimated 3.5–5.9% of the population, are serious conditions with complex etiologies and genetic underpinnings [1]. Individuals with RDs often experience lengthy diagnostic delays, averaging 5–9 years [2–6], leading to prolonged burden due to ineffective pre-diagnostic treatments [7–9] and substantial health care utilization, including an estimated 30 million discharges from inpatient, readmission and emergency services annually [10]. This underscores the need for significant and innovative changes across all specialties that will result in shorter time to diagnosis. The current review characterizes individuals with RDs seeking psychiatric care, who may initially present with neuropsychiatric symptoms prior to receiving their RD diagnosis.

Managing RDs with neuropsychiatric symptoms requires a focused approach to shorten diagnostic delay and ensure individuals receive appropriate therapies. Persons with RDs may be labeled as “treatment resistant” when their neuropsychiatric symptoms are clinically similar to primary mental health conditions that may be present for months or years before the presentation of other condition-defining symptoms [11]. These symptoms may be unremitting when treated with the same ‘standard of care medications’ traditionally. The diagnostic delay is further complicated by individuals that respond to symptomatic management but may require optimization with disease-specific treatments to improve patient outcomes. The RDs identified in this review are accompanied by neuropsychiatric symptoms and may require medical workup that is outside of guidelines for initial patient encounters. This challenge is compounded by limited payor reimbursement and recommendations that include genetic testing [12, 13] that may delineate individuals with RDs who present with neuropsychiatric symptoms compared to those affected by primary mental health conditions.

Underlying this issue of mis- or non-diagnosis at the intersection of mental health and rare disease is a dearth of scientific information about, and clinical exposure to, many RDs, which has led to the potentially harmful off-label prescribing of drugs or suboptimal symptomatic medication management. Of particular concern regarding treatment of delayed or mis-diagnosed RDs is the use of selective serotonin reuptake inhibitors (SSRIs), antipsychotics, and atypical antipsychotics which can carry a black box warning and have metabolic side effects [14]. For example, some conditions such as Phelan-McDermid syndrome involve a deletion that typically includes coding for CYP2D6, an enzyme that plays a role in the metabolism of many psychiatric medications, which may be a contributing factor to the consensus guidelines that suggest avoiding SSRIs due to the high risk of side effects [15, 16]. Collectively, mental health outcomes may be highly influenced by an underlying rare disease status.

To effectively address this problem, it is critically important to identify and characterize RDs that present with mental health symptoms prior to affected persons being considered treatment- or medication-resistant. The presenting symptom burden may reflect the primary symptoms of the disease itself or represent neuropsychiatric symptoms that develop secondary to the RD. It is worth noting the high lifetime prevalence of affective disorders (46%) reported in persons with a chronic RD [17], which may reflect secondary illnesses resulting from the diagnostic odyssey or disease burden. Importantly, for a subset of RDs, neuropsychiatric symptoms may be better understood as a fundamental presenting symptom of the underlying RD itself, reflecting a complex interplay of genetic, metabolic, neurobiological, and psychological factors. Clarifying this association is essential to improving diagnostic accuracy and therapeutic strategies in psychiatric settings.

Genetic screening clinical decision-making tools are one solution to help distinguish between the symptoms caused by the RD and those symptoms of a primary mental health condition, particularly with 72–80% of RDs having some level of genetic involvement [1]. With decreasing genetic testing costs, there may be benefits to utilizing a genetic screen prior to prescribing psychiatric medications, particularly for monogenic conditions that can be screened with exome sequencing. Therefore, in this scoping review the authors identified 108 unique RDs that are candidates for inclusion in a mental health genetic screen, that may aid health care providers across multiple specialties in making more accurate diagnoses and decreasing the burden experienced by individuals affected by these conditions.

## Methods

### Data source

A scoping review was used to guide the synthesis of evidence across broad and heterogenous published data for the conditions under review. A comprehensive list of rare conditions was primarily obtained from Orphanet [18], a global rare disease database, and the Online Mendelian Inheritance in Man (OMIM) catalog of human genes and genetic disorders [19]. To identify RDs with documented neuropsychiatric symptoms and to maximize the inclusion of articles in the review, MEDLINE was searched for each condition, including alternative names for each condition obtained from the OMIM and Orphanet disease databases. The final search strings included free-text words (e.g. treatment, time to diagnosis, age of onset, neuropsychiatric symptoms, prevalence) combined with Boolean operators, for example “condition name” OR “alternative name(s)” AND “diagnostic delay”. Data were extracted from a range of peer-reviewed sources, including cohort studies, clinical studies, case series and case reports. For each rare genetic disease, preference was given to studies reporting diagnostic delays quantified using large patient samples.

### Condition selection

Condition selection criteria were restrictive to align with manifestation of RDs that are usually diagnosed in childhood and adulthood. Those RDs that are universally fatal or typically diagnosed within the first three years of life were excluded from the analysis. For example, early-onset Tay-Sachs disease was omitted, while the late-onset subtype was included due to its diagnostic challenges later in life. RDs were further restricted to monogenic conditions, with a few exceptions for polygenic conditions where specific genes have a clearly defined role in neuropsychiatric presentations. For instance, conditions like the behavioral variant of frontotemporal dementia (bvFTD) with involvement of *chromosome 9 open reading frame 72* (*C9orf72*) and *progranulin* (*GRN*) were included. Conversely, chromosomal conditions such as Prader-Willi and Williams syndromes were excluded, as their genetic complexity lies outside the scope of this study’s objectives. Additionally, at least some individuals must initially present with neuropsychiatric symptoms to be included. These symptoms spanned a wide spectrum, including mood and anxiety disorders (e.g., anxiety, depression, and bipolar disorder), developmental and behavioral conditions (e.g., autism spectrum disorder [ASD]), attention-deficit/hyperactivity disorder [ADHD], and stereotypies), and impulse-control and obsessive–compulsive behaviors (e.g., trichotillomania, excoriation disorder, and obsessive–compulsive disorder [OCD]). Symptoms of psychotic spectrum disorders (e.g., psychosis, hallucinations, and delusions), cognitive and intellectual impairments (e.g., memory decline, cognitive impairment, and intellectual disability), and self-harm and aggression (e.g., self-injurious behavior, suicidal ideation, and aggression) were also included. Other symptoms such as sleep disturbances and substance use disorders were evaluated when they appeared as presenting symptoms. This comprehensive approach ensured that the conditions analyzed accurately represented the intersection of rare diseases and neuropsychiatric manifestations.

To standardize the analysis of diagnostic delay, the age of onset was defined as the age at which the first symptom of the rare disease appeared, based on descriptions in the literature. When the age of onset was not explicitly reported, it was defined as the age at which the first author-reported symptom was documented (e.g., the patient began experiencing motor difficulties and memory decline at age 21). The age at diagnosis referred to the age at which a definitive diagnosis was established using genetic or clinical criteria. The diagnostic delay was then calculated as the time elapsed between the age of onset and the age at diagnosis. To align with the review’s objective of screening these conditions across diverse treatment settings, the prevalence and presentation of neuropsychiatric symptoms was recorded across all cases but excluded from the calculation of diagnostic delay.

### Calculation of cost and utilization

Healthcare Cost and Utilization Project (HCUP) data from 2019 was used for original research to demonstrate the degree of impact of the selected conditions in health care. Data was extracted from the Nationwide Inpatient Sample (NIS), Kids’ Inpatient Database (KID), Nationwide Readmissions Database (NRD), and Nationwide Emergency Department Sample (NEDS) databases using the International Classification of Diseases, Tenth Revision (ICD-10) codes linked to RDs and Diagnosis-related group (DRG codes). The DRG codes utilized focused on mental health and addiction, which included all codes 880–897. The ICD-10 and ICD, Eleventh Revision (ICD-11) codes were primarily determined using Orphanet, OMIM, and Kyoto Encyclopedia of Genes and Genomes (KEGG). At the time of analysis, the most recent data set that included the KID database was 2019.

The databases covered a broad range of health care utilization, with the NIS as the largest all-payor healthcare database of inpatient data from 98% of discharges in the United States (U.S.), including encounters, charges, diagnostic-related group codes, demographics, and other key cost-related data. The 2019 NIS database included 35.4 million hospital discharges from 48 states plus the District of Columbia (D.C.) [20]. The KID subset reported similar information for pediatric and transitional age youth (TAY) inpatient charges from patients younger than 21 years of age. The 2019 KID database included 3.1 million hospital discharges [21]. The NRD includes 1.8 million hospital discharges and was used to estimate 30-day, all-cause hospital readmissions [22]. The NEDS database included 33.1 million hospital discharges derived from 995 hospitals in 40 states and D.C. [23] All n’s in each database are estimated and the analysis done for each data element can vary due to missing values. A breakdown of cost and discharge by ICD-10 and DRG codes is provided (Table S1).

## Results

### Diagnostic delay and age of onset

**Fig. 1:**
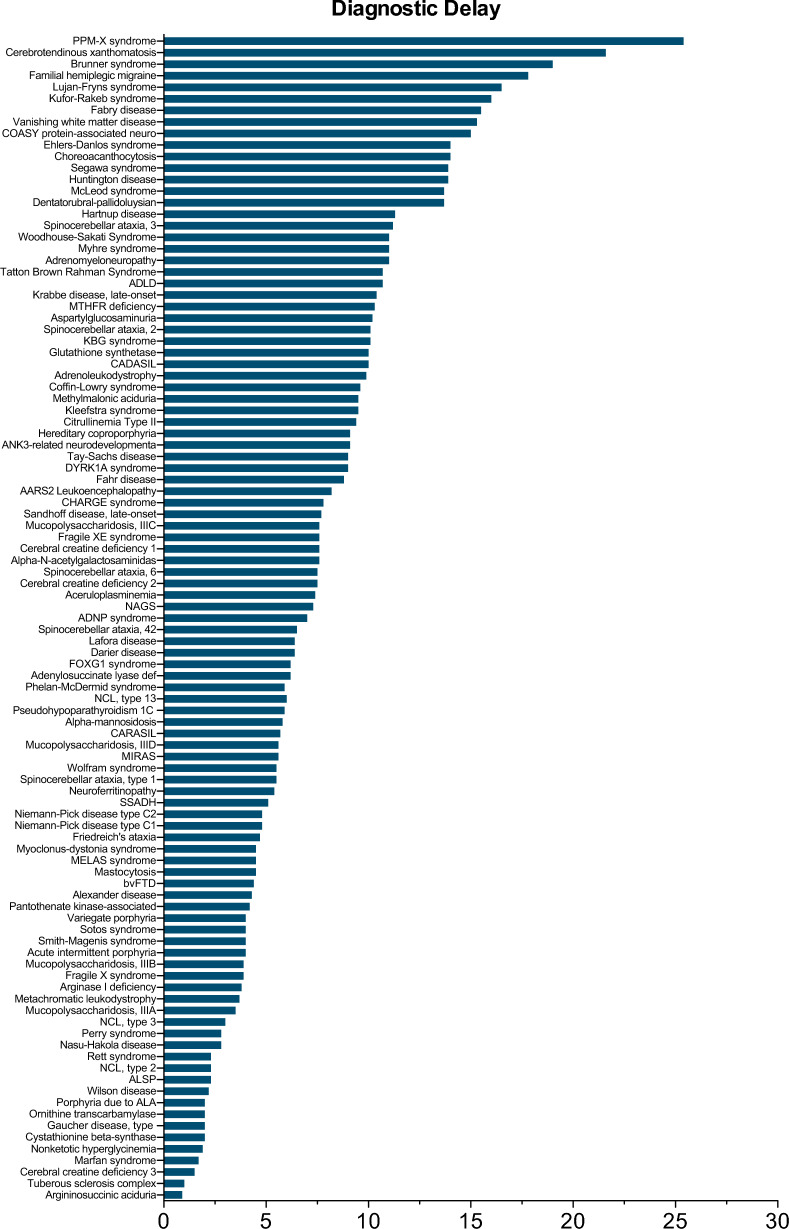
Average diagnostic delay by rare genetic disease. The average diagnostic delay of all 108 rare diseases was 7.7 ± 4.6 years, range 0.3–25.4 years

The diagnostic odyssey experienced by individuals with RDs continues to be arduous, delaying optimized clinical management and resulting in significant psychosocial and medical burden [24]. Diagnostic delay averaged 7.7 ± 4.6 years, with averages ranging from 0.3 to 25.4 years across the selected conditions (Fig. 1). Subanalysis revealed variations by biological area with leukodystrophies averaging 9.4 ± 5.3 years; neurodevelopmental conditions averaging 9.1 ± 5.7 years; neurological conditions averaging 8.4 ± 4.6 years; metabolic conditions averaging 6.4 ± 4.1 years; and the remaining (other) conditions averaging 4.8 ± 1.9 (Table S2). Age of onset varied widely from infancy through adulthood (Table S3), with 31% having an onset in adolescence or adulthood. The diagnostic delay likely involves a combination of clinical factors, including variable age of onset and neuropsychiatric presentation, as well as socioeconomic factors that include 52% of individuals using public paper insurance (data not shown). Given the limited direct association between RDs and mental health, apart from secondary depression, anxiety and other conditions related to chronic illness management, characterizing RDs that may initially present with neuropsychiatric symptoms across diverse biological areas is central to raising awareness of their heterogenous presentations.

### Classification of rare diseases by biological involvement

**Fig. 2:**
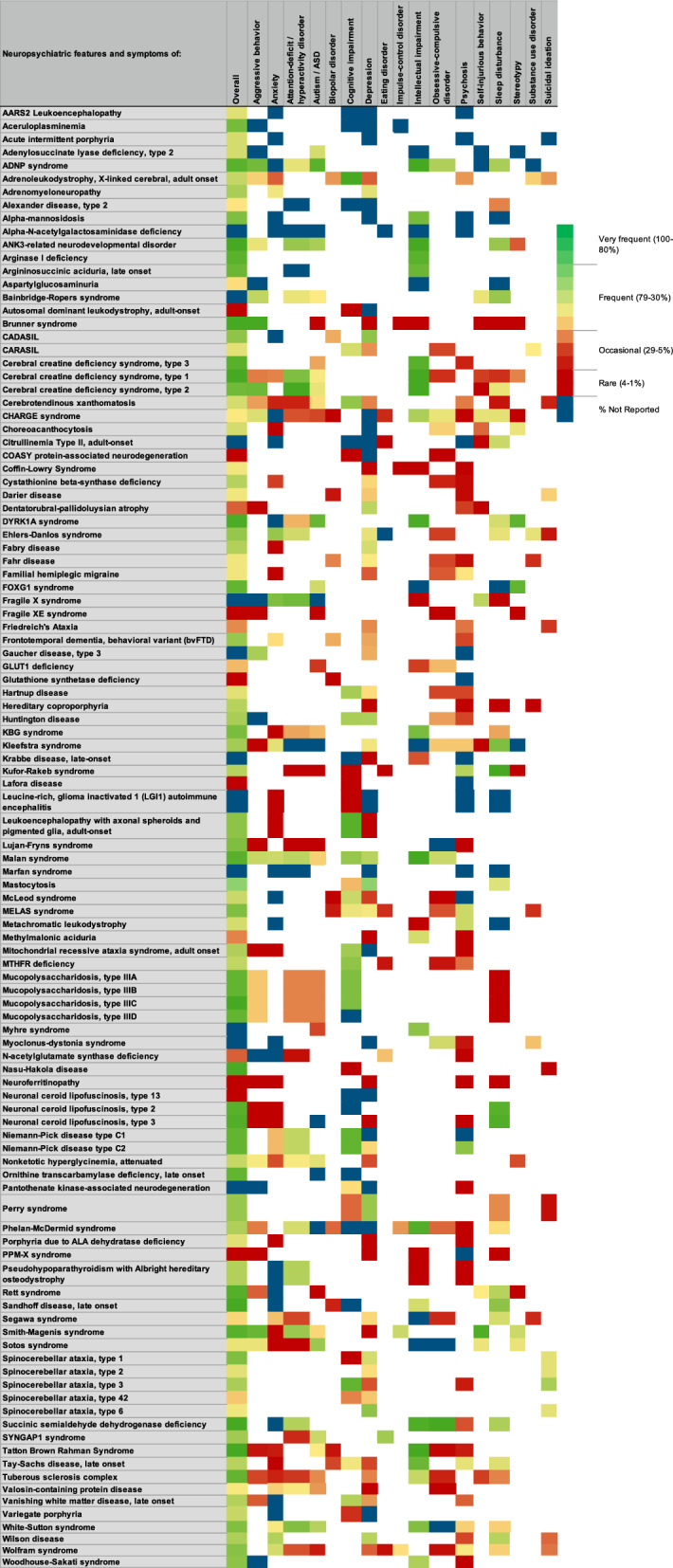
Neuropsychiatric symptoms reported for all 108 conditions. Symptoms are categorized either as individual symptoms or as symptoms of the listed condition. The symptom frequency is represented using a color-coded key linked to specific frequency ranges. Rare genetic diseases where the percentage was not reported for the patient population is indicated in dark blue

Delineating neuropsychiatric symptoms caused by an underlying RD from those that due to comorbid mental health disorders can be improved by linking the neuropsychiatric symptoms to the underlying pathophysiology. Focusing on biological classifications that incorporate a full range of presenting symptoms, including neurological, vascular, and others, may aid in the differential diagnosis. It may also help guide the medical workup and subsequent medication management for each condition. The neuropsychiatric symptoms for each rare disease were represented using a color-coded key linked to reported frequency ranges (Fig. 2).

#### Leukodystrophies

Leukodystrophies are genetic multisystem diseases, characterized by progressive white matter degeneration, and often include substantial morbidity and mortality concerns. In the central nervous system (CNS), alterations to peroxisomal, lysosomal, and lipid storage can lead to neurodegeneration of white matter in the brain and spinal cord. This leads to a broad range of cognitive, neuropsychiatric, sensory and neuromuscular problems. For most leukodystrophies, treatments were traditionally supportive; however, advancements in gene therapies, hemopoietic stem cell transplants, and others have decreased mortality and morbidity [25–27]. Twelve conditions were identified (Table S3), including AARS2 leukodystrophy, adult-onset X-linked adrenoleukodystrophy (X-ALD, adult onset) and adrenomyeloneuropathy (AMN), autosomal dominant leukodystrophy with autonomic disease (ADLD), type 2 Alexander disease, cerebrotendinous xanthomatosis, adult-onset Krabbe disease, CADASIL, CARASIL, metachromatic leukodystrophy, adult-onset leukoencephalopathy with axonal spheroids and pigmented glia, and late-onset vanishing white matter disease. The onset of X-ALD, AMN, ADLD, CADASIL, and CARASIL tend to be in the 30s and 40 s, and have a prolonged delay before a diagnosis is made. Those affected often have neuropsychiatric manifestations [28]. Adult-onset X-ALD, AMN, and ADLD account for approximately half of all adrenoleukodystrophy subtypes [29]. These conditions tend to progress more slowly than other subtypes, such as childhood cerebral X-ALD, and can present with neuropsychiatric symptoms, including depression, anxiety, OCD, and dementia, as the initial presenting symptoms [30, 31]. Cerebrotendinous xanthomatosis and metachromatic leukodystrophy also can present with psychosis, hallucinations, and the milder symptoms of disorganized thinking and unexpected decreased performance in school [32–36]. Those affected by type 2 Alexander disease, unlike the much more severe neonatal or infantile type 1 [37], have an average life expectancy of 30 years [38]. However, during that time a diverse set of neuropsychiatric symptoms are frequently observed in the population including depression, self-injurious behavior and sleep disturbances. Evidence is emerging that these symptoms may arise from the neurodegeneration in brain regions and neural networks that correspond to these symptoms [39], although significant work remains to delineate the neuropsychiatric—brain region relationship.

#### Metabolic conditions

Inherited metabolic conditions, or diseases with inborn errors of metabolism, represent a diverse range of RDs. Many of the 40 identified conditions are complex with broad pathology (Table S4). The early and accurate identification of these conditions can have a profound impact on morbidity and mortality for those affected. For example, in addition to an initial presenting concern such as autism, individuals affected by glycine encephalopathy also can present with seizures. Of great concern is that medications often prescribed for seizures and comorbid psychiatric symptoms, including valproic acid, are contraindicated for and can lead to increased seizures, coma, or even death [40, 41]. Another condition with significant treatment concerns is adult-onset Neiman-Pick Type C1, which is noted to present with schizophrenia-like symptoms that include auditory hallucinations and paranoid delusions for years without other classic Niemann-Pick symptoms. Reports of these patients have revealed that they may be diagnosed with schizophrenia, prescribed schizophrenia-indicated medications, and undergo electroconvulsive therapy treatments for years without symptom improvement [42]. However, these “treatment resistant” neuropsychiatric symptoms appear to improve from treatment with the enzyme inhibitor, miglustat. Some other metabolic conditions may have acute, but potentially profound, symptoms that may be severe enough for psychiatric inpatient admission or emergency department visits, such as the porphyrias that include acute intermittent porphyria, porphyria variegate, hereditary coproporphyria, and porphyria due to ALA dehydratase deficiency [43]. Batten disease, or neuronal ceroid lipofuscinosis, is another condition with multiple subtypes with hallmark neuropsychiatric symptoms ranging from depression and anxiety to aggressive behavior [44] (Table S5).

#### Neurodevelopmental conditions

Neurodevelopmental disorders are complex genetic conditions that are particularly symptomatic during brain development. Fifteen (15) conditions identified (Table S3), almost all monogenic, typically involve disrupted neural and glial signal transduction. Typical symptoms of autism (e.g., social reciprocity and restrictive repetitive behaviors) and aggressive behaviors were the most common symptoms among these conditions; however, attention-deficit/hyperactivity disorder (ADHD), stereotypies, and sleep problems were also present in many of the selected RDs. Onset ranged from infancy to adolescence, with the exception of Smith-Magenis syndrome [45] that routinely presents in adults. Despite the earlier developmental timeline and with an increasing societal focus on autism, the average diagnostic delay ranged from 2.3 years for Rett syndrome [46] to an average of 25.4 years for the ultra-rare condition PPM-X syndrome [47–49] (Fig. 1). It has been postulated that increasing comprehensive genetic testing, such as exome sequencing, specifically in the neurodevelopmental space, may address many of these delays [50, 51].

#### Neurological conditions

The current review of neurological RDs identified serious conditions with neuropsychiatric symptoms. Out of the 32 RDs identified, the average diagnostic delay ranged from 0.3 to 17.8 years (Fig. 1). These conditions are characterized by their biological underpinnings such as neurodegenerative pathologies, disruption in mitochondrial function, and disturbances of neural signal transduction. It is important to note that these pathologies are the result of considerable complexity that is not fully understood; however, many of the conditions responded to symptomatic management (Table S6). Additionally, the age of onset tended to be later for these conditions, with the onset of 16 of 28 neurological RDs occurring in adolescence or adulthood, while only 9 RDs had an onset in infancy or childhood.

#### Other conditions

Additional conditions were classified as cardiovascular, dermatologic, endocrine, and hematologic type conditions, all which include neuropsychiatric involvement. Their diverse biology led to unique presentations. For example, despite classically being described as an autosomal dominant skin condition, Darier disease is also frequently associated with neuropsychiatric symptoms [52]. Marfan syndrome, a well-described connective tissue disorder, presents with neuropsychiatric symptoms ranging from schizophrenia-like behavior, depression, and anxiety to ADHD [53–55]. These examples underscore the necessity of broadened genetic testing, such as exome sequencing, and enhanced incorporation of physical observations such as craniofacial abnormalities, dermatological markings, and other features into physical examinations to distinguish symptoms of a primary mental health condition from those of the selected RDs. Furthermore, revising the assessment criteria for these conditions to encompass these neuropsychiatric symptoms may facilitate diagnosis, particularly for providers in pediatrics, family medicine, and psychiatry. The successful implementation of expanded genetic testing is likely to expedite the detection of these conditions, reducing diagnostic delays and enabling the prompt initiation of condition-specific treatment.

### Should an innovative strategy around this relatively small number of RDs within psychiatry be developed?

**Fig. 3:**
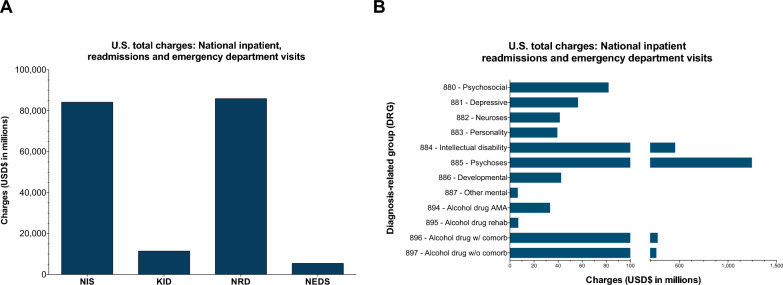
Estimated total charges for US health-care utilization of rare genetic diseases (RDs), with all charges in USD. **A** Estimated total charges for RDs (in millions USD) for the Nationwide Inpatient Sample (NIS), Kids’ Inpatient Database (KID), Nationwide Readmissions Database (NRD), and NEDS. **B** Estimated total charges per diagnosis-related group (DRG) codes for RD inpatient stay from NRD and NEDS were combined. All estimated charges were broken down by DRG code and reported in millions ($)

A key consideration for the selected 108 conditions was to determine their impact in health care in terms of utilization and cost, particularly when receiving care related to the neuropsychiatric symptoms. Individually, the prevalence rates for most of the selected conditions was low yet underscored disproportionate health care utilization. They ranged from 1–5 per 10,000 to < 1 per 1,000,000 (Table S4). To broadly estimate the health care utilization of those affected, national data was analyzed to estimate the number of annual discharges using NIS (inpatient), KID (pediatric inpatient), NRD (readmission), and NEDS (emergency) data sources. A total of 892,110 inpatient discharges were reported in the NIS for the ICD-10 codes associated with the 108 RDs. Furthermore, pediatric/TAY inpatient admissions reported 152,049 discharges in the KID database. Readmissions were common, with 948,432 readmissions in 2019. There was significant utilization of emergency services with 999,871 discharges reported (Table 1). The charges (in USD) were substantial, totaling an estimated $181 billion in 2019, with inpatient charges reaching $84 billion, which was 4% of the total charges of all conditions in 2019 (Fig. 3a). Additionally, there was $5 billion in emergency department charges, $86 billion in readmission charges and $11 billion specifically for pediatric/TAY inpatient stays in 2019 (Table 1). The charges and dischargers per ICD-10 code used were further described in Table S1. This highlights utilization across a range of medical specialties, which may provide the initial diagnosis and associated treatment for individuals affected by the RDs.

**Table 1:** Utilization and demographic characteristics, weighted estimate in USD ($)

	National inpatient stay (NIS)	Kids inpatient database (KID)	Nationwide readmissions database (NRD)	National emergency department sample (NEDS)
Discharges	892,110	188,747	948,432	999,871
Charges				
Total in aggregate	$84,183,760,711	$11,475,748,123	$85,925,794,740	$5,435,898,660
Average per discharge	$94,972	$61,228	$90,598	$6,614
Diagnosis related group: Mental health (880–897) in aggregate	$1,714,012,979	$91,497,350	$2,481,824,491	$70,784,259
Length of stay (days)	7.9	5.8	8.0	
Age average (y)	52.3	3.4	51.5	58.7
Sex (%)				
Female	48.8	49.0	49.3	48.6
Male	51.2	51.0	50.7	51.4
Race and ethnicity (%)				
White	60.4	30.4		65.6
Black	19.5	28.4		18.9
Hispanic	11.7	23.8		10.0
Asian or Pacific Islander	3.8	8.4		2.3
Native American	0.7	0.7		0.5
Other	3.9	8.3		2.8
Payer (%)				
Private	19.0	33.8	18.0	15.9
Medicare	51.5	0.4	52.1	58.3
Medicaid	23.4	58.2	24.0	19.5
Self-pay	3.4	4.8	3.0	4.0
No charge	0.2	0.1	0.2	0.2
Other	2.5	2.8	2.6	2.1
Discharge disposition (%)				
Routine	45.2	92.2	48.3	27.9
Transfer to short-term hospital	2.8	1.8	1.3	2.5
Transfer to other facility	30.0	1.9	27.5	3.0
Home health care	13.6	3.3	14.6	1.0
Left against medical advice	1.1	0.1	1.2	0.5
Died	7.2	0.7	7.0	0.1
Admitted inpatient				64.9
Charges per discharge, USD ($)				
Routine	23,049,060,467	8,075,466,418	24,638,939,193	2,656,856,543
Transfer to short-term hospital	3,119,134,714	753,813,123	1,807,953,777	137,424,608
Transfer to other facility	35,355,571,396	947,572,354	32,973,123,601	576,861,710
Home health care	11,793,630,930	1,131,212,016	14,669,708,102	316,600,056
Left against medical advice	648,315,023	14,342,262	744,173,987	118,208,104
Died	10,148,319,038	528,970,695	10,951,729,336	151,122,660
Admitted inpatient				3,402,633,231

Missing and non-reported values not displayed

To estimate the cost and utilizations for patients receiving care for mental health and addiction services, the charges were then stratified by DRG codes. These codes were specific to inpatient stays, where the primary billing code was for all related conditions that included psychosis, major depressive disorder, bipolar disorder, alcohol-use disorder, Fragile X syndrome and others. A total of $1.7 billion for NIS, $91 million for KID, $2.5 billion for NRD and $71 million for NEDS inpatient stays and further broken down by DRG code (Table 1). Readmissions charges were the most impact ful, with the greatest impact with inpatient stays related to treating psychotic disorders (Fig. 3b). Collectively, this suggests that the selected conditions have disproportionate utilization in health care.

## Discussion

Our review of the literature found 108 conditions that spanned 20 different disease areas (e.g., urea cycle disorders) (Table S4) and included a diverse array of neuropsychiatric symptoms (e.g., depression, anxiety, aggressive behavior; Table S5) that resemble symptoms of primary mental health diagnoses. While patient presentation may differ between the rare disease and the common conditions for which they may be (mis)diagnosed, the heterogeneity of these conditions makes differential diagnosis challenging for health care providers. This issue is amplified due to the limited information available to clinicians, such as qualitative assessments that include psychological measurements, family and patient history, and parental- or self-description of symptoms.

### Rethinking the label treatment-resistant without evaluating genetic conditions

Pharmacological intervention is a key component of the standard of care for most psychiatric conditions. However, studies also show that many affected individuals do not adequately respond to psychotropic medications if the underlying etiology remains unaddressed (Table S6). Treatment- or medication-resistance has been documented across the spectrum of mental health conditions, including major depressive disorder, schizophrenia [56], and obsessive–compulsive disorder [57, 58], and is associated with worsened clinical outcomes and increased health care costs, particularly during the diagnostic delay. There is a clear need for more effective and personalized treatment approaches, including genetic testing, to be more accessible for providers managing patients with behavioral health needs.

### Symptom management may delay condition-specific treatment

By their very nature, specific RDs are observed infrequently, with many physicians perceiving their knowledge on RDs as insufficient or very poor [59]. As a result of this limited exposure, particularly in psychiatry, substantial delays in diagnosis are common. One comprehensive study showed that individuals with a RD experience an average diagnostic delay of 7.6 years, and visit an average of 8 health care providers prior to diagnosis [60]. This highlights the issue where specialists, such as psychiatrists, assume that the medical workup has been completed by primary care providers prior to referral.

At admission into behavioral health care programs or during the initial evaluation, affected individuals may be in crisis or present with urgent needs related to their mental health when presenting to psychiatrists and genetic testing may be overlooked. Psychiatrists and other medical professionals are typically trained to follow guidelines and best practices to treat a condition and presenting symptoms, without a specific focus on whether the condition is rare. Additionally, providers often work with limited historical information regarding the experiences of affected individuals seeking treatment outside of their insurance network or geographical region during their diagnostic journey. An affected individual may be trialed on multiple medications after being diagnosed with different disorders over time, which may result in side-effects (e.g., weight gain, dyslipidemia) without improvement in symptoms. Furthermore, drug-drug interactions may result in decreased efficacy of treatment and increased burnout in families. Practice parameters and guidelines developed by major specialty groups highlight this approach. This is an important distinction from the present review, which is aimed at highlighting the continued need to identify rare genetic diseases within the patient population so that indicated treatments are provided with optimal timing, decreasing patient suffering and system costs. In the general population, using genetic information remains a challenge due to the potential impact of novel and causal variants on the sensitivity and specificity of genetic tests.

We propose that expanded genetic testing integrated earlier in the treatment protocol can offer significant benefits in guiding medication selection for mental health patients, without creating false positives when non-pathogenetic variants are detected. To maximize its effectiveness, we suggest implementing strategies to integrate easy access to screening for providers, which may be used early in treatment. While future investigation is necessary to determine the optimal timepoint, this screening may occur when patients do not respond to the first or second medication, as well as patients identified by their provider on a case-by-case basis. This approach could improve the time to diagnosis and contribute to the base of knowledge of both RDs and psychiatric disease. Notably, recent evidence has suggested a two-fold enrichment of RDs in psychiatric populations compared to the general population, highlighting the diagnostic opportunity that exists when RD-focused tools are implemented in mental health care settings [61]. Large-scale implementation studies have further shown that integrating genome and transcriptome sequencing in clinical workflows can significantly increase diagnostic yield and inform medical decision-making [62, 63]. By integrating genotypic and phenotypic information with the medical workup, more accurate diagnoses can be provided, leading to improved treatment outcomes for each patient. If specifically done within the mental health space, this allows psychiatrists, clinical psychologists, and advanced practitioners to better lead an interdisciplinary team to address the behavioral, cognitive, and biological components of patients to improve their neuropsychiatric symptoms and quality of life.

## Conclusions

The involvement of neuropsychiatric symptoms in the selected RDs suggests that a significant population of individuals seeking mental health treatment may have an underlying rare disease. The primary objective of this review is to explore a novel opportunity: the detection of RDs in mental health patients through the development of a cost-effective genetic testing platform with an exome sequencing backbone. This platform is designed to detect RDs with neuropsychiatric symptoms that may guide providers towards disease-modifying treatments, dietary adjustments, or the incorporation of standardized disease monitoring protocols into treatment plans. By focusing on these specific conditions, the genetic screening tool avoids the many ethical concerns and the potential for false positives or false negatives associated with genome sequencing. This approach better enables psychiatrists, clinical geneticists, and other specialists to diagnose a range of genetic conditions that are typically not assessed but may be affecting patients’ responses to standard psychiatric medications. Ultimately, this innovative genetic screening approach will reduce the time to diagnosis, optimize medical follow-up procedures, provide timely best-in-class therapies, substantially reduce the healthcare costs, and decrease the suffering associated with the diagnostic odyssey of individuals affected by RDs with core neuropsychiatric symptoms.

## Limitations

- Diagnostic delay is not frequently reported, resulting in some studies that pre-date the genetic panels that can be utilized as of the time of publication of this review.
- The neuropsychiatric symptoms of the identified conditions may be most pronounced with a later age of onset, or when the disease severity is mild.
- Neuropsychiatric symptoms are not frequently reported and therefore the actual presentation percentages may differ as case studies and older descriptive studies were included.
- The ICD-10 codes utilized to determine healthcare cost and utilization has multiple limitations that include, but are not limited to, a single code may be used for multiple additional conditions that were not investigated, undiagnosed patients would not have an associated ICD-10 diagnostic code, and a single patient may have multiple codes.
- Individual rare diseases may be associated with multiple biological classifications, which was not further characterized in the review.

## Supplementary Information

Additional file 1.

Additional file 2.

Additional file 3.

Additional file 4.

Additional file 5.

Additional file 6.

Additional file 7.

## Data Availability

All data generated or analyzed during this study are included in this published article [and its supplementary information files].

## Abbreviations

ADHD: Attention-deficit/hyperactivity disorder
AMN: Adrenomyeloneuropathy
ASD: Autism spectrum disorder
CNS: Central nervous system
DRG: Diagnosis-related group
HCUP: Health care cost and utilization
ICD-10: International classification of diseases, tenth revision
ICD-11: International classification of diseases, eleventh revision
KID: Kids’ inpatient database
NEDS: Nationwide emergency department sample
NHGRI: National Human Genome Research Institute
NIS: Nationwide inpatient sample
NRD: Nationwide readmissions database
OCD: Obsessive–compulsive disorder
OMIM: Online Mendelian inheritance in man
RD: Rare disease
RUSP: Recommended uniform screening program
SSRI: Selective serotonin reuptake inhibitor
TAY: Transitional age youth
U.S.: United States
USD: United States Dollar
X-ALD: X-linked adrenoleukodystrophy
