# Supplementary material for: Bridging psychiatry and rare genetic diseases: a scoping review of therapeutic strategies and diagnostic delay paired with healthcare economic burden analysis
Source: Orphanet J Rare Dis. 2025 Aug 1;20:393. doi: 10.1186/s13023-025-03941-8 (PMC12315305; doi:10.1186/s13023-025-03941-8)
Supplement: Supplementary file 1 — Additional file 1. [file 13023_2025_3941_MOESM1_ESM.docx]

**SUPPLEMENTARY REFERENCES**

64. Axelsen TM, Vammen TL, Bak M, Pourhadi N, Stenor CM, Gronborg S: **Case report: 'AARS2 leukodystrophy'**. *Mol Genet Metab Rep* 2021, **28**:100782.

65. Uzun GA: **Adult-onset leukodystrophy with homozygous AARS2 mutation located in the aminoacylation domain**. *Neurol India* 2019, **67**(3):871-872.

66. Parra SP, Heckers SH, Wilcox WR, McKnight CD, Jinnah HA: **The emerging neurological spectrum of AARS2-associated disorders**. *Parkinsonism Relat Disord* 2021, **93**:50-54.

67. Vila Cuenca M, Marchi G, Barque A, Esteban-Jurado C, Marchetto A, Giorgetti A, Chelban V, Houlden H, Wood NW, Piubelli C *et al*: **Genetic and Clinical Heterogeneity in Thirteen New Cases with Aceruloplasminemia. Atypical Anemia as a Clue for an Early Diagnosis**. *Int J Mol Sci* 2020, **21**(7).

68. Vroegindeweij LHP, Langendonk JG, Langeveld M, Hoogendoorn M, Kievit AJA, Di Raimondo D, Wilson JHP, Boon AJW: **New insights in the neurological phenotype of aceruloplasminemia in Caucasian patients**. *Parkinsonism Relat Disord* 2017, **36**:33-40.

69. Fasano A, Colosimo C, Miyajima H, Tonali PA, Re TJ, Bentivoglio AR: **Aceruloplasminemia: a novel mutation in a family with marked phenotypic variability**. *Mov Disord* 2008, **23**(5):751-755.

70. Haemers I, Kono S, Goldman S, Gitlin JD, Pandolfo M: **Clinical, molecular, and PET study of a case of aceruloplasminaemia presenting with focal cranial dyskinesia**. *J Neurol Neurosurg Psychiatry* 2004, **75**(2):334-337.

71. Loreal O, Turlin B, Pigeon C, Moisan A, Ropert M, Morice P, Gandon Y, Jouanolle AM, Verin M, Hider RC *et al*: **Aceruloplasminemia: new clinical, pathophysiological and therapeutic insights**. *J Hepatol* 2002, **36**(6):851-856.

72. Chretien F, Servan J, Mikol J, Trierweiller M, Elghozi D, Gray F: **A 70-year-old man with extrapyramidal symptoms, dementia and hemosiderosis**. *Brain Pathol* 2006, **16**(3):235-236.

73. Bosio S, De Gobbi M, Roetto A, Zecchina G, Leonardo E, Rizzetto M, Lucetti C, Petrozzi L, Bonuccelli U, Camaschella C: **Anemia and iron overload due to compound heterozygosity for novel ceruloplasmin mutations**. *Blood* 2002, **100**(6):2246-2248.

74. Bjork MH, Gjerde IO, Tzoulis C, Ulvik RJ, Bindoff LA: **A man in his 50s with high ferritin levels and increasing cognitive impairment**. *Tidsskr Nor Laegeforen* 2015, **135**(15):1369-1372.

75. Hellman NE, Schaefer M, Gehrke S, Stegen P, Hoffman WJ, Gitlin JD, Stremmel W: **Hepatic iron overload in aceruloplasminaemia**. *Gut* 2000, **47**(6):858-860.

76. Dunaief JL, Richa C, Franks EP, Schultze RL, Aleman TS, Schenck JF, Zimmerman EA, Brooks DG: **Macular degeneration in a patient with aceruloplasminemia, a disease associated with retinal iron overload**. *Ophthalmology* 2005, **112**(6):1062-1065.

77. Skidmore FM, Drago V, Foster P, Schmalfuss IM, Heilman KM, Streiff RR: **Aceruloplasminaemia with progressive atrophy without brain iron overload: treatment with oral chelation**. *J Neurol Neurosurg Psychiatry* 2008, **79**(4):467-470.

78. Mykletun M, Aarsand AK, Stole E, Villanger JH, Tollanes MC, Baravelli C, Sandberg S: **Porphyrias in Norway**. *Tidsskr Nor Laegeforen* 2014, **134**(8):831-836.

79. Mastrogiorgio G, Macchiaiolo M, Buonuomo PS, Bellacchio E, Bordi M, Vecchio D, Brown KP, Watson NK, Contardi B, Cecconi F *et al*: **Clinical and molecular characterization of patients with adenylosuccinate lyase deficiency**. *Orphanet J Rare Dis* 2021, **16**(1):112.

80. Gozes I, Patterson MC, Van Dijck A, Kooy RF, Peeden JN, Eichenberger JA, Zawacki-Downing A, Bedrosian-Sermone S: **The Eight and a Half Year Journey of Undiagnosed AD: Gene Sequencing and Funding of Advanced Genetic Testing Has Led to Hope and New Beginnings**. *Front Endocrinol (Lausanne)* 2017, **8**:107.

81. Iqbal Z, Vandeweyer G, van der Voet M, Waryah AM, Zahoor MY, Besseling JA, Roca LT, Vulto-van Silfhout AT, Nijhof B, Kramer JM *et al*: **Homozygous and heterozygous disruptions of ANK3: at the crossroads of neurodevelopmental and psychiatric disorders**. *Hum Mol Genet* 2013, **22**(10):1960-1970.

82. Chen LJ, You ZM, Chen WH, Yang S, Feng CC, Wang HY, Wang T, Zhu YY: **Helsmoortel-van der Aa syndrome in a Chinese pediatric patient due to ADNP nonsense mutation: A case report**. *Front Pediatr* 2023, **11**:1122513.

83. Szabo TM, Balogh I, Ujfalusi A, Szucs Z, Madar L, Koczok K, Bessenyei B, Csurke I, Szakszon K: **Helsmoortel-Van der Aa Syndrome-Cardiothoracic and Ectodermal Manifestations in Two Patients as Further Support of a Previous Observation on Phenotypic Overlap with RASopathies**. *Genes (Basel)* 2022, **13**(12).

84. Shillington A, Pedapati E, Hopkin R, Suhrie K: **Early behavioral and developmental interventions in ADNP-syndrome: A case report of SWI/SNF-related neurodevelopmental syndrome**. *Mol Genet Genomic Med* 2020, **8**(6):e1230.

85. Huynh MT, Boudry-Labis E, Massard A, Thuillier C, Delobel B, Duban-Bedu B, Vincent-Delorme C: **A heterozygous microdeletion of 20q13.13 encompassing ADNP gene in a child with Helsmoortel-van der Aa syndrome**. *Eur J Hum Genet* 2018, **26**(10):1497-1501.

86. Pescosolido MF, Schwede M, Johnson Harrison A, Schmidt M, Gamsiz ED, Chen WS, Donahue JP, Shur N, Jerskey BA, Phornphutkul C *et al*: **Expansion of the clinical phenotype associated with mutations in activity-dependent neuroprotective protein**. *J Med Genet* 2014, **51**(9):587-589.

87. Gale MJ, Titus HE, Harman GA, Alabduljalil T, Dennis A, Wilson JL, Koeller DM, Finanger E, Blasco PA, Chiang PW *et al*: **Longitudinal ophthalmic findings in a child with Helsmoortel-Van der Aa Syndrome**. *Am J Ophthalmol Case Rep* 2018, **10**:244-248.

88. Van Dijck A, Vulto-van Silfhout AT, Cappuyns E, van der Werf IM, Mancini GM, Tzschach A, Bernier R, Gozes I, Eichler EE, Romano C *et al*: **Clinical Presentation of a Complex Neurodevelopmental Disorder Caused by Mutations in ADNP**. *Biol Psychiatry* 2019, **85**(4):287-297.

89. van Geel BM, Assies J, Haverkort EB, Barth PG, Wanders RJ, Schutgens RB, Keyser A, Zwetsloot CP: **Delay in diagnosis of X-linked adrenoleukodystrophy**. *Clin Neurol Neurosurg* 1993, **95**(2):115-120.

90. Pareyson D, Fancellu R, Mariotti C, Romano S, Salmaggi A, Carella F, Girotti F, Gattellaro G, Carriero MR, Farina L *et al*: **Adult-onset Alexander disease: a series of eleven unrelated cases with review of the literature**. *Brain* 2008, **131**(Pt 9):2321-2331.

91. Zielonka M, Garbade SF, Kolker S, Hoffmann GF, Ries M: **Ultra-orphan lysosomal storage diseases: A cross-sectional quantitative analysis of the natural history of alpha-mannosidosis**. *J Inherit Metab Dis* 2019, **42**(5):975-983.

92. Lipinski P, Rozdzynska-Swiatkowska A, Iwanicka-Pronicka K, Perkowska B, Pokora P, Tylki-Szymanska A: **Long-term outcome of patients with alpha-mannosidosis - A single center study**. *Mol Genet Metab Rep* 2022, **30**:100826.

93. Belanger-Quintana A, Martinez-Pardo M, Garcia MJ, Wermuth B, Torres J, Pallares E, Ugarte M: **Hyperammonaemia as a cause of psychosis in an adolescent**. *Eur J Pediatr* 2003, **162**(11):773-775.

94. Cavicchi C, Chilleri C, Fioravanti A, Ferri L, Ripandelli F, Costa C, Calabresi P, Prontera P, Pochiero F, Pasquini E *et al*: **Late-Onset N-Acetylglutamate Synthase Deficiency: Report of a Paradigmatic Adult Case Presenting with Headaches and Review of the Literature**. *Int J Mol Sci* 2018, **19**(2).

95. Kanzaki T, Wang AM, Desnick RJ: **Lysosomal alpha-N-acetylgalactosaminidase deficiency, the enzymatic defect in angiokeratoma corporis diffusum with glycopeptiduria**. *J Clin Invest* 1991, **88**(2):707-711.

96. Kodama K, Kobayashi H, Abe R, Ohkawara A, Yoshii N, Yotsumoto S, Fukushige T, Nagatsuka Y, Hirabayashi Y, Kanzaki T: **A new case of alpha-N-acetylgalactosaminidase deficiency with angiokeratoma corporis diffusum, with Meniere's syndrome and without mental retardation**. *Br J Dermatol* 2001, **144**(2):363-368.

97. Posset R, Garbade SF, Boy N, Burlina AB, Dionisi-Vici C, Dobbelaere D, Garcia-Cazorla A, de Lonlay P, Teles EL, Vara R *et al*: **Transatlantic combined and comparative data analysis of 1095 patients with urea cycle disorders-A successful strategy for clinical research of rare diseases**. *J Inherit Metab Dis* 2019, **42**(1):93-106.

98. Al Kaabi EH, El-Hattab AW: **N-acetylglutamate synthase deficiency: Novel mutation associated with neonatal presentation and literature review of molecular and phenotypic spectra**. *Mol Genet Metab Rep* 2016, **8**:94-98.

99. Ah Mew N, Caldovic L: **N-acetylglutamate synthase deficiency: an insight into the genetics, epidemiology, pathophysiology, and treatment**. *Appl Clin Genet* 2011, **4**:127-135.

100. Kloth K, Lozic B, Tagoe J, Hoffer MJV, Van der Ven A, Thiele H, Altmuller J, Kubisch C, Au PYB, Denecke J *et al*: **ANK3 related neurodevelopmental disorders: expanding the spectrum of heterozygous loss-of-function variants**. *Neurogenetics* 2021, **22**(4):263-269.

101. Keshavan N, Wood M, Alderson LM, Cortina-Borja M, Skeath R, McSweeney M, Dixon M, Cleary MA, Footitt E, Batzios S: **Clinical status, biochemical profile and management of a single cohort of patients with arginase deficiency**. *JIMD Rep* 2022, **63**(2):123-130.

102. Freua F, Almeida MEC, Nobrega PR, Paiva ARB, Della-Ripa B, Cunha P, Macedo-Souza LI, Bueno C, Lynch DS, Houlden H *et al*: **Arginase 1 deficiency presenting as complicated hereditary spastic paraplegia**. *Cold Spring Harb Mol Case Stud* 2022, **8**(6).

103. Osawa Y, Wada A, Ohtsu Y, Yamada K, Takizawa T: **Late-onset argininosuccinic aciduria associated with hyperammonemia triggered by influenza infection in an adolescent: A case report**. *Mol Genet Metab Rep* 2020, **24**:100605.

104. Yamamoto T, Shimojima K, Matsufuji M, Mashima R, Sakai E, Okuyama T: **Aspartylglucosaminuria caused by a novel homozygous mutation in the AGA gene was identified by an exome-first approach in a patient from Japan**. *Brain Dev* 2017, **39**(5):422-425.

105. Vargas-Diez E, Chabas A, Coll MJ, Sanchez-Perez J, Garcia-Diez A, Fernandez-Herrera JM: **Angiokeratoma corporis diffusum in a Spanish patient with aspartylglucosaminuria**. *Br J Dermatol* 2002, **147**(4):760-764.

106. Labate A, Barone R, Gambardella A, Civitelli D, Fiumara A, Annesi G, Zappia M, Pavone L, Quattrone A: **Startle epilepsy complicating aspartylglucosaminuria**. *Brain Dev* 2004, **26**(2):130-133.

107. Kartal A, Aydin K: **Brain MRI findings in two Turkish pediatric patients with aspartylglucosaminuria**. *Neuroradiol J* 2016, **29**(5):310-313.

108. Mezaki N, Miura T, Ogaki K, Eriguchi M, Mizuno Y, Komatsu K, Yamazaki H, Suetsugi N, Kawajiri S, Yamasaki R *et al*: **Duplication and deletion upstream of LMNB1 in autosomal dominant adult-onset leukodystrophy**. *Neurol Genet* 2018, **4**(6):e292.

109. Verhoeven W, Egger J, Rakers E, van Erkelens A, Pfundt R, Willemsen MH: **Phenotypic characterization of an older adult male with late-onset epilepsy and a novel mutation in ASXL3 shows overlap with the associated Bainbridge-Ropers syndrome**. *Neuropsychiatr Dis Treat* 2018, **14**:867-870.

110. Giri D, Rigden D, Didi M, Peak M, McNamara P, Senniappan S: **Novel compound heterozygous ASXL3 mutation causing Bainbridge-ropers like syndrome and primary IGF1 deficiency**. *Int J Pediatr Endocrinol* 2017, **2017**:8.

111. Koboldt DC, Mihalic Mosher T, Kelly BJ, Sites E, Bartholomew D, Hickey SE, McBride K, Wilson RK, White P: **A de novo nonsense mutation in ASXL3 shared by siblings with Bainbridge-Ropers syndrome**. *Cold Spring Harb Mol Case Stud* 2018, **4**(3).

112. Chinen Y, Nakamura S, Ganaha A, Hayashi S, Inazawa J, Yanagi K, Nakanishi K, Kaname T, Naritomi K: **Mild prominence of the Sylvian fissure in a Bainbridge-Ropers syndrome patient with a novel frameshift variant in ASXL3**. *Clin Case Rep* 2018, **6**(2):330-336.

113. Sumeyra Naralan Y, Enes Naralan M, Hocaoglu C: **Bainbridge Ropers Syndrome as a Rare Cause of Autism Spectrum Disorder**. *Psychiatr Danub* 2023, **35**(4):582-586.

114. Kuechler A, Czeschik JC, Graf E, Grasshoff U, Huffmeier U, Busa T, Beck-Woedl S, Faivre L, Riviere JB, Bader I *et al*: **Bainbridge-Ropers syndrome caused by loss-of-function variants in ASXL3: a recognizable condition**. *Eur J Hum Genet* 2017, **25**(2):183-191.

115. Siu Xiao T, Colombari Arce G, Rojas Marron A, Benitez GA, Schwanecke R: **Bainbridge-Ropers Syndrome in a Texan Boy: A Case Report and Review of the Literature**. *Cureus* 2022, **14**(12):e32902.

116. Li JR, Huang Z, Lu Y, Ji QY, Jiang MY, Yang F: **Novel mutation in the ASXL3 gene in a Chinese boy with microcephaly and speech impairment: A case report**. *World J Clin Cases* 2020, **8**(24):6465-6472.

117. Cuddapah VA, Dubbs HA, Adang L, Kugler SL, McCormick EM, Zolkipli-Cunningham Z, Ortiz-Gonzalez XR, McCormack S, Zackai E, Licht DJ *et al*: **Understanding the phenotypic spectrum of ASXL-related disease: Ten cases and a review of the literature**. *Am J Med Genet A* 2021, **185**(6):1700-1711.

118. Ikekwere JC, Osuagwu FC, LePlatte D, Ghaziuddin M: **Comorbid Psychiatric Aspects of Bainbridge-Ropers Syndrome**. *Prim Care Companion CNS Disord* 2021, **23**(3).

119. Myers KA, White SM, Mohammed S, Metcalfe KA, Fry AE, Wraige E, Vasudevan PC, Balasubramanian M, Scheffer IE: **Childhood-onset generalized epilepsy in Bainbridge-Ropers syndrome**. *Epilepsy Res* 2018, **140**:166-170.

120. Contreras-Capetillo SN, Vilchis-Zapata ZH, Ribbon-Conde J, Pinto-Escalante D: **Global developmental delay and postnatal microcephaly: Bainbridge-Ropers syndrome with a new mutation in ASXL3**. *Neurologia (Engl Ed)* 2018, **33**(7):484-486.

121. Kuseyri Hubschmann O, Horvath G, Cortes-Saladelafont E, Yildiz Y, Mastrangelo M, Pons R, Friedman J, Mercimek-Andrews S, Wong SN, Pearson TS *et al*: **Insights into the expanding phenotypic spectrum of inherited disorders of biogenic amines**. *Nat Commun* 2021, **12**(1):5529.

122. Piton A, Poquet H, Redin C, Masurel A, Lauer J, Muller J, Thevenon J, Herenger Y, Chancenotte S, Bonnet M *et al*: **20 ans apres: a second mutation in MAOA identified by targeted high-throughput sequencing in a family with altered behavior and cognition**. *Eur J Hum Genet* 2014, **22**(6):776-783.

123. Ortiz-Garcia J, Orjuela K, Sweis R, Biller J: **Misdiagnosis of CADASIL: A single Medical Center experience (P1.251)**. *Neurology* 2017, **88**(16 Supplement):P1.251.

124. Liu JY, Zhu YC, Zhou LX, Wei YP, Mao CH, Cui LY, Peng B, Yao M: **HTRA1-related autosomal dominant cerebral small vessel disease**. *Chin Med J (Engl)* 2020, **134**(2):178-184.

125. Morey K, Hallinan B, Cecil KM: **Case report: Clinical and magnetic resonance spectroscopy presentation of a female severely affected with X-linked creatine transporter deficiency**. *Radiol Case Rep* 2022, **17**(4):1115-1119.

126. Cervera-Acedo C, Lopez M, Aguirre-Lamban J, Santibanez P, Garcia-Oguiza A, Poch-Olive ML, Dominguez-Garrido E: **A novel SLC6A8 mutation associated with motor dysfunction in a child exhibiting creatine transporter deficiency**. *Hum Genome Var* 2015, **2**:15037.

127. Rostami P, Hosseinpour S, Ashrafi MR, Alizadeh H, Garshasbi M, Tavasoli AR: **Primary creatine deficiency syndrome as a potential missed diagnosis in children with psychomotor delay and seizure: case presentation with two novel variants and literature review**. *Acta Neurol Belg* 2020, **120**(3):511-516.

128. Cheillan D, Joncquel-Chevalier Curt M, Briand G, Salomons GS, Mention-Mulliez K, Dobbelaere D, Cuisset JM, Lion-Francois L, Portes VD, Chabli A *et al*: **Screening for primary creatine deficiencies in French patients with unexplained neurological symptoms**. *Orphanet J Rare Dis* 2012, **7**:96.

129. Hinnell C, Samuel M, Alkufri F, Ashkan K, Rahman Y, Turner C, Dalton RN, Nashef L: **Creatine deficiency syndromes: diagnostic pearls and pitfalls**. *Can J Neurol Sci* 2011, **38**(5):765-767.

130. Khaikin Y, Sidky S, Abdenur J, Anastasi A, Ballhausen D, Buoni S, Chan A, Cheillan D, Dorison N, Goldenberg A *et al*: **Treatment outcome of twenty-two patients with guanidinoacetate methyltransferase deficiency: An international retrospective cohort study**. *Eur J Paediatr Neurol* 2018, **22**(3):369-379.

131. Ndika JD, Johnston K, Barkovich JA, Wirt MD, O'Neill P, Betsalel OT, Jakobs C, Salomons GS: **Developmental progress and creatine restoration upon long-term creatine supplementation of a patient with arginine:glycine amidinotransferase deficiency**. *Mol Genet Metab* 2012, **106**(1):48-54.

132. Battini R, Leuzzi V, Carducci C, Tosetti M, Bianchi MC, Item CB, Stockler-Ipsiroglu S, Cioni G: **Creatine depletion in a new case with AGAT deficiency: clinical and genetic study in a large pedigree**. *Mol Genet Metab* 2002, **77**(4):326-331.

133. Pilo-de-la-Fuente B, Jimenez-Escrig A, Lorenzo JR, Pardo J, Arias M, Ares-Luque A, Duarte J, Muniz-Perez S, Sobrido MJ: **Cerebrotendinous xanthomatosis in Spain: clinical, prognostic, and genetic survey**. *Eur J Neurol* 2011, **18**(10):1203-1211.

134. Verrips A, Dotti MT, Mignarri A, Stelten BML, Verma S, Federico A: **The safety and effectiveness of chenodeoxycholic acid treatment in patients with cerebrotendinous xanthomatosis: two retrospective cohort studies**. *Neurol Sci* 2020, **41**(4):943-949.

135. Antoniou MC, Bouthors T, Xu C, Phan-Hug F, Elowe-Gruau E, Stoppa-Vaucher S, Sloot AV, Acierno J, Cassatella D, Richard C *et al*: **A novel CHD7 mutation in an adolescent presenting with growth and pubertal delay**. *Ann Pediatr Endocrinol Metab* 2019, **24**(1):49-54.

136. Galvez-Ruiz A, Galindo-Ferreiro A, Lehner AJ: **CHARGE syndrome: A case report of two new CDH7 gene mutations**. *Saudi J Ophthalmol* 2020, **34**(4):306-309.

137. Wells C, Loundon N, Garabedian N, Wiener-Vacher S, Cordier-Bouvier MD, Goudeffroye G, Attie-Bitach T, Marlin S: **A case of mild CHARGE syndrome associated with a splice site mutation in CHD7**. *Eur J Med Genet* 2016, **59**(4):195-197.

138. Benninger F, Afawi Z, Korczyn AD, Oliver KL, Pendziwiat M, Nakamura M, Sano A, Helbig I, Berkovic SF, Blatt I: **Seizures as presenting and prominent symptom in chorea-acanthocytosis with c.2343del VPS13A gene mutation**. *Epilepsia* 2016, **57**(4):549-556.

139. Bharucha EP, Bharucha NE: **Choreo-acanthocytosis**. *J Neurol Sci* 1989, **89**(2-3):135-139.

140. Kyo M, Mii H, Takekita Y, Tokuhara D, Yazaki M, Nakamori Y, Kono S, Kinoshita T: **Case of adult-onset type II citrullinemia treated as schizophrenia for a long time**. *Psychiatry Clin Neurosci* 2015, **69**(5):306-307.

141. Oshiro S, Kochinda T, Tana T, Yamazato M, Kobayashi K, Komine Y, Muratani H, Saheki T, Iseki K, Takishita S: **A patient with adult-onset type II citrullinemia on long-term hemodialysis: reversal of clinical symptoms and brain MRI findings**. *Am J Kidney Dis* 2002, **39**(1):189-192.

142. Annesi G, Gagliardi M, Iannello G, Quattrone A, Iannello G, Quattrone A: **Mutational analysis of COASY in an Italian patient with NBIA**. *Parkinsonism Relat Disord* 2016, **28**:150-151.

143. Dusi S, Valletta L, Haack TB, Tsuchiya Y, Venco P, Pasqualato S, Goffrini P, Tigano M, Demchenko N, Wieland T *et al*: **Exome sequence reveals mutations in CoA synthase as a cause of neurodegeneration with brain iron accumulation**. *Am J Hum Genet* 2014, **94**(1):11-22.

144. Arslan EA, Ceylaner S, Turanli G: **Stimulus-induced myoclonus treated effectively with clonazepam in genetically confirmed Coffin-Lowry syndrome**. *Epilepsy Behav Case Rep* 2014, **2**:196-198.

145. Jin H, Li H, Qiang S: **Coffin-Lowry Syndrome Induced by RPS6KA3 Gene Variation in China: A Case Report in Twins**. *Medicina (Kaunas)* 2022, **58**(7).

146. Lange IR, Stone P, Aftimos S: **The Coffin-Lowry syndrome: a case report and review of the literature**. *J Obstet Gynaecol Can* 2010, **32**(7):691-694.

147. Lv Y, Zhu L, Zheng J, Wu D, Shao J: **Growth Concerns in Coffin-Lowry Syndrome: A Case Report and Literature Review**. *Front Pediatr* 2018, **6**:430.

148. Hirakawa M, Nishihara T, Nakanishi K, Kitamura S, Fujii S, Ikemune K, Dote K, Takasaki Y, Yorozuya T: **Perioperative management of a patient with Coffin-Lowry syndrome complicated by severe obesity: A case report and literature review**. *Medicine (Baltimore)* 2017, **96**(49):e9026.

149. Yamoto K, Saitsu H, Fujisawa Y, Kato F, Matsubara K, Fukami M, Kagami M, Ogata T: **Coffin-Lowry syndrome in a girl with 46,XX,t(X;11)(p22;p15)dn: Identification of RPS6KA3 disruption by whole genome sequencing**. *Clin Case Rep* 2020, **8**(6):1076-1080.

150. Tise CG, Matalon DR, Manning MA, Byers HM, Grover M: **Short Bones, Renal Stones, and Diagnostic Moans: Hypercalcemia in a Girl Found to Have Coffin-Lowry Syndrome**. *J Investig Med High Impact Case Rep* 2022, **10**:23247096221101844.

151. Di Stazio M, Bigoni S, Iuso N, Vuch J, Selvatici R, Ulivi S, d'Adamo PA: **Identification of a New Mutation in RSK2, the Gene for Coffin-Lowry Syndrome (CLS), in Two Related Patients with Mild and Atypical Phenotypes**. *Brain Sci* 2021, **11**(8).

152. Moura EB, Moura EL, Amorim FF, Oliveira VM: **Mechanical ventilation in Coffin-Lowry syndrome: a case report**. *Rev Bras Ter Intensiva* 2016, **28**(4):483-487.

153. Welborn M, Farrell S, Knott P, Mayekar E, Mardjetko S: **The natural history of spinal deformity in patients with Coffin-Lowry syndrome**. *J Child Orthop* 2018, **12**(1):70-75.

154. Bras A, Pedruco A, Maia S, Fineza I, Morgadinho A, Bento C: **A Bizarre Gait as a Result of Overlapping Functional Disorder With Coffin-Lowry Syndrome**. *Mov Disord Clin Pract* 2019, **6**(6):491-493.

155. Singh PM, Baidya DK, Govindarajan S, Trikha A: **Ocular surgery in a child with Coffin Lowry syndrome: Anesthetic concerns**. *J Anaesthesiol Clin Pharmacol* 2013, **29**(1):114-116.

156. Concannon N, Hegarty AM, Stallings RL, Reardon W: **Coffin-Lowry phenotype in a patient with a complex chromosome rearrangement**. *J Med Genet* 2002, **39**(8):e41.

157. Manouvrier-Hanu S, Amiel J, Jacquot S, Merienne K, Moerman A, Coeslier A, Labarriere F, Vallee L, Croquette MF, Hanauer A: **Unreported RSK2 missense mutation in two male sibs with an unusually mild form of Coffin-Lowry syndrome**. *J Med Genet* 1999, **36**(10):775-778.

158. Field M, Tarpey P, Boyle J, Edkins S, Goodship J, Luo Y, Moon J, Teague J, Stratton MR, Futreal PA *et al*: **Mutations in the RSK2(RPS6KA3) gene cause Coffin-Lowry syndrome and nonsyndromic X-linked mental retardation**. *Clin Genet* 2006, **70**(6):509-515.

159. Kozich V, Sokolova J, Morris AAM, Pavlikova M, Gleich F, Kolker S, Krijt J, Dionisi-Vici C, Baumgartner MR, Blom HJ *et al*: **Cystathionine beta-synthase deficiency in the E-HOD registry-part I: pyridoxine responsiveness as a determinant of biochemical and clinical phenotype at diagnosis**. *J Inherit Metab Dis* 2021, **44**(3):677-692.

160. Yeshurun A, Ziv M, Cohen-Barak E, Vered S, Rozenman D, Sah M, Khayat M, Polyakov O, Amichai B, Zlotogorski A *et al*: **An Update on the Cutaneous Manifestations of Darier Disease**. *J Cutan Med Surg* 2021, **25**(5):498-503.

161. Tunc S, Tadic V, Zuhlke C, Hellenbroich Y, Bruggemann N: **Pearls & Oy-sters: Family history of Huntington disease disguised a case of dentatorubral-pallidoluysian atrophy**. *Neurology* 2018, **90**(3):142-143.

162. Braga-Neto P, Pedroso JL, Furtado GV, Gheno TC, Saraiva-Pereira ML, Jardim LB, Barsottini OGP, Rede N: **Dentatorubro-Pallidoluysian Atrophy (DRPLA) among 700 Families with Ataxia in Brazil**. *Cerebellum* 2017, **16**(4):812-816.

163. Le Ber I, Camuzat A, Castelnovo G, Azulay JP, Genton P, Gastaut JL, Broglin D, Labauge P, Brice A, Durr A: **Prevalence of dentatorubral-pallidoluysian atrophy in a large series of white patients with cerebellar ataxia**. *Arch Neurol* 2003, **60**(8):1097-1099.

164. Wardle M, Majounie E, Williams NM, Rosser AE, Morris HR, Robertson NP: **Dentatorubral pallidoluysian atrophy in South Wales**. *J Neurol Neurosurg Psychiatry* 2008, **79**(7):804-807.

165. Qiao F, Shao B, Wang C, Wang Y, Zhou R, Liu G, Meng L, Hu P, Xu Z: **A De Novo Mutation in DYRK1A Causes Syndromic Intellectual Disability: A Chinese Case Report**. *Front Genet* 2019, **10**:1194.

166. Luco SM, Pohl D, Sell E, Wagner JD, Dyment DA, Daoud H: **Case report of novel DYRK1A mutations in 2 individuals with syndromic intellectual disability and a review of the literature**. *BMC Med Genet* 2016, **17**:15.

167. Murray CR, Abel SN, McClure MB, Foster J, 2nd, Walke MI, Jayakar P, Bademci G, Tekin M: **Novel Causative Variants in DYRK1A, KARS, and KAT6A Associated with Intellectual Disability and Additional Phenotypic Features**. *J Pediatr Genet* 2017, **6**(2):77-83.

168. Moller RS, Kubart S, Hoeltzenbein M, Heye B, Vogel I, Hansen CP, Menzel C, Ullmann R, Tommerup N, Ropers HH *et al*: **Truncation of the Down syndrome candidate gene DYRK1A in two unrelated patients with microcephaly**. *Am J Hum Genet* 2008, **82**(5):1165-1170.

169. van Bon BW, Hoischen A, Hehir-Kwa J, de Brouwer AP, Ruivenkamp C, Gijsbers AC, Marcelis CL, de Leeuw N, Veltman JA, Brunner HG *et al*: **Intragenic deletion in DYRK1A leads to mental retardation and primary microcephaly**. *Clin Genet* 2011, **79**(3):296-299.

170. Oegema R, de Klein A, Verkerk AJ, Schot R, Dumee B, Douben H, Eussen B, Dubbel L, Poddighe PJ, van der Laar I *et al*: **Distinctive Phenotypic Abnormalities Associated with Submicroscopic 21q22 Deletion Including DYRK1A**. *Mol Syndromol* 2010, **1**(3):113-120.

171. Ruaud L, Mignot C, Guet A, Ohl C, Nava C, Heron D, Keren B, Depienne C, Benoit V, Maystadt I *et al*: **DYRK1A mutations in two unrelated patients**. *Eur J Med Genet* 2015, **58**(3):168-174.

172. A K, F F: **The voice of 12,000 patients: experiences and expectations of rare disease patients on diagnosis and care in Europe.** In*.* Eurodis; 2009.

173. Mehta A, Ricci R, Widmer U, Dehout F, Garcia de Lorenzo A, Kampmann C, Linhart A, Sunder-Plassmann G, Ries M, Beck M: **Fabry disease defined: baseline clinical manifestations of 366 patients in the Fabry Outcome Survey**. *Eur J Clin Invest* 2004, **34**(3):236-242.

174. Barba-Romero MA, Rivera-Gallego A, Pintos-Morell G, Spanish FOSSG: **Fabry disease in Spain: description of Spanish patients and a comparison with other European countries using data from the Fabry Outcome Survey (FOS)**. *Int J Clin Pract* 2011, **65**(8):903-910.

175. Andrikos E, Iatrou C, Boletis JN, Diamandopoulos A, Katsinas C, Kalaitzidis K, Galinas A, Xaidara A, Pappas M, Siamopoulos KC: **Evolution of Fabry disease in male patients: the Greek experience**. *Clin Nephrol* 2010, **73**(1):58-63.

176. Marchesoni CL, Roa N, Pardal AM, Neumann P, Caceres G, Martinez P, Kisinovsky I, Bianchi S, Tarabuso AL, Reisin RC: **Misdiagnosis in Fabry disease**. *J Pediatr* 2010, **156**(5):828-831.

177. Mengel E, Gaedeke J, Gothe H, Krupka S, Lachmann A, Reinke J, Ohlmeier C: **The patient journey of patients with Fabry disease, Gaucher disease and Mucopolysaccharidosis type II: A German-wide telephone survey**. *PLoS One* 2020, **15**(12):e0244279.

178. Wang H, Shao B, Wang L, Ye Q: **Fahr's disease in two siblings in a family: A case report**. *Exp Ther Med* 2015, **9**(5):1931-1933.

179. Asokan AG, D'Souza S, Jeganathan J, Pai S: **Fahr's Syndrome- An Interesting Case Presentation**. *J Clin Diagn Res* 2013, **7**(3):532-533.

180. Dela Cruz W, Aguirre A: **Fahr's Disease in a 26 year-old female: A case report**. *Journal of the Neurological Sciences* 2017, **381**:799-800.

181. Durante A, Audino N, Cristiano M, Tanga M, Martino MT, Noschese I, D'Auria D, Pinto F: **Basal ganglia calcification: a Fahr's disease case report**. *Radiol Case Rep* 2021, **16**(10):3055-3059.

182. Thillaigovindan R, Arumugam E, Rai R, R P, Kesavan R: **Idiopathic Basal Ganglia Calcification: Fahr's Syndrome, a Rare Disorder**. *Cureus* 2019, **11**(10):e5895.

183. Castagna A, Ruberto C, Cerra RP, Greco L, Ruotolo G: **Fahr’s disease: familial idiopathic basal ganglia calcification with and without extrapyramidal disorders**. *Geriatric Care* 2020, **6**(2).

184. Ahmed MA, Reid E, Cooke A, Arngrimsson R, Tolmie JL, Stephenson JB: **Familial hemiplegic migraine in the west of Scotland: a clinical and genetic study of seven families**. *J Neurol Neurosurg Psychiatry* 1996, **61**(6):616-620.

185. Indelicato E, Nachbauer W, Eigentler A, Donnemiller E, Wagner M, Unterberger I, Boesch S: **Ten years of follow-up in a large family with familial hemiplegic migraine type 1: Clinical course and implications for treatment**. *Cephalalgia* 2018, **38**(6):1167-1176.

186. Shoichet SA, Kunde SA, Viertel P, Schell-Apacik C, von Voss H, Tommerup N, Ropers HH, Kalscheuer VM: **Haploinsufficiency of novel FOXG1B variants in a patient with severe mental retardation, brain malformations and microcephaly**. *Hum Genet* 2005, **117**(6):536-544.

187. Ariani F, Hayek G, Rondinella D, Artuso R, Mencarelli MA, Spanhol-Rosseto A, Pollazzon M, Buoni S, Spiga O, Ricciardi S *et al*: **FOXG1 is responsible for the congenital variant of Rett syndrome**. *Am J Hum Genet* 2008, **83**(1):89-93.

188. Mencarelli MA, Spanhol-Rosseto A, Artuso R, Rondinella D, De Filippis R, Bahi-Buisson N, Nectoux J, Rubinsztajn R, Bienvenu T, Moncla A *et al*: **Novel FOXG1 mutations associated with the congenital variant of Rett syndrome**. *J Med Genet* 2010, **47**(1):49-53.

189. Philippe C, Amsallem D, Francannet C, Lambert L, Saunier A, Verneau F, Jonveaux P: **Phenotypic variability in Rett syndrome associated with FOXG1 mutations in females**. *J Med Genet* 2010, **47**(1):59-65.

190. Mitter D, Pringsheim M, Kaulisch M, Plumacher KS, Schroder S, Warthemann R, Abou Jamra R, Baethmann M, Bast T, Buttel HM *et al*: **FOXG1 syndrome: genotype-phenotype association in 83 patients with FOXG1 variants**. *Genet Med* 2018, **20**(1):98-108.

191. Gabis LV, Hochberg O, Leon Attia O, Banet-Levi Y, Topf D, Shefer S: **Prolonged Time Lag to Final Diagnosis of Fragile X Syndrome**. *J Pediatr* 2018, **193**:217-221 e211.

192. Krishnan V, Clouston P, Crocker M, Macpherson J, Heydon F, Stewart H: **Fragile XE: an important differential diagnosis**. *BMJ Case Rep* 2010, **2010**.

193. Ahmed I, Ilyas M, Harlalka GV, Mir A: **A Novel Hemizygous Variant in the AFF2 Gene Causing Fragile XE (FRAXE) Syndrome: First Report from Pakistan**. *Pakistan Journal of Medical Research* 2021, **60**(2):85-89.

194. Stettner GM, Shoukier M, Hoger C, Brockmann K, Auber B: **Familial intellectual disability and autistic behavior caused by a small FMR2 gene deletion**. *Am J Med Genet A* 2011, **155A**(8):2003-2007.

195. Indelicato E, Nachbauer W, Eigentler A, Amprosi M, Matteucci Gothe R, Giunti P, Mariotti C, Arpa J, Durr A, Klopstock T *et al*: **Onset features and time to diagnosis in Friedreich's Ataxia**. *Orphanet J Rare Dis* 2020, **15**(1):198.

196. Zapata-Restrepo L, Rivas J, Miranda C, Miller BL, Ibanez A, Allen IE, Possin K: **The Psychiatric Misdiagnosis of Behavioral Variant Frontotemporal Dementia in a Colombian Sample**. *Front Neurol* 2021, **12**:729381.

197. Coyle-Gilchrist IT, Dick KM, Patterson K, Vazquez Rodriquez P, Wehmann E, Wilcox A, Lansdall CJ, Dawson KE, Wiggins J, Mead S *et al*: **Prevalence, characteristics, and survival of frontotemporal lobar degeneration syndromes**. *Neurology* 2016, **86**(18):1736-1743.

198. Thomas AS, Mehta AB, Hughes DA: **Diagnosing Gaucher disease: an on-going need for increased awareness amongst haematologists**. *Blood Cells Mol Dis* 2013, **50**(3):212-217.

199. Robertson PL, Buchanan DN, Muenzer J: **5-Oxoprolinuria in an adolescent with chronic metabolic acidosis, mental retardation, and psychosis**. *J Pediatr* 1991, **118**(1):92-95.

200. Melamed I, Kobayashi RH, O'Connor M, Kobayashi AL, Schechterman A, Heffron M, Canterberry S, Miranda H, Rashid N: **Evaluation of Intravenous Immunoglobulin in Pediatric Acute-Onset Neuropsychiatric Syndrome**. *J Child Adolesc Psychopharmacol* 2021, **31**(2):118-128.

201. Kolic I, Radic Nisevic J, Vlasic Cicvaric I, Butorac Ahel I, Lah Tomulic K, Segulja S, Baraba Dekanic K, Serifi S, Ovuka A, Prpic I: **GLUT1 Deficiency Syndrome-Early Treatment Maintains Cognitive Development? (Literature Review and Case Report)**. *Genes (Basel)* 2021, **12**(9).

202. Gburek-Augustat J, Heinze A, Abou Jamra R, Merkenschlager A: **Hemiplegic Migraine in Glut1 Deficiency Syndrome and Paroxysmal Dyskinesia at Ketogenic Diet Induction: Case Report and Literature Review**. *Mov Disord Clin Pract* 2020, **7**(8):965-970.

203. Furia A, Muccioli L, Santucci M, Licchetta L, Bisulli F: **Pseudohyperkalemia due to cryohydrocytosis in GLUT1 deficiency syndrome. A case report and literature review**. *Epileptic Disord* 2023, **25**(3):410-415.

204. Sandu C, Burloiu CM, Barca DG, Magureanu SA, Craiu DC: **Ketogenic Diet in Patients with GLUT1 Deficiency Syndrome**. *Maedica (Bucur)* 2019, **14**(2):93-97.

205. Bozkurt T, Alanay Y, Isik U, Sezerman U: **Re-analysis of whole-exome sequencing data reveals a novel splicing variant in the SLC2A1 in a patient with GLUT1 Deficiency Syndrome 1 accompanied by hemangioma: a case report**. *BMC Med Genomics* 2021, **14**(1):197.

206. Hu Q, Shen Y, Su T, Liu Y, Xu S: **Clinical and Genetic Characteristics of Chinese Children With GLUT1 Deficiency Syndrome: Case Report and Literature Review**. *Front Genet* 2021, **12**:734481.

207. Pearson TS, Pons R, Engelstad K, Kane SA, Goldberg ME, De Vivo DC: **Paroxysmal eye-head movements in Glut1 deficiency syndrome**. *Neurology* 2017, **88**(17):1666-1673.

208. Kim YS, Kim W, Na JH, Lee YM: **Nutritional Intervention Through Ketogenic Diet in GLUT1 Deficiency Syndrome**. *Clin Nutr Res* 2023, **12**(3):169-176.

209. Narvaez C, Lacaux P, Cortes C, Manterola C, Carrasco X: **Phenotypic variability of GLUT1 deficiency: When is necessary to suspect?** *Rev Chil Pediatr* 2020, **91**(2):260-264.

210. Kraoua I, Benrhouma H, Vuillaumier-Barrot S, Klaa H, Youssef-Turki IB: **A Case of Progressive Chorea Resulting From GLUT1 Deficiency**. *Mov Disord Clin Pract* 2015, **2**(4):424-425.

211. Rohatgi S, Rao P, Nirhale S, Naphade P, Dubey P: **GLUT-1 Deficiency Syndrome; HSP Mimic: A Case Report**. *Ann Indian Acad Neurol* 2023, **26**(3):307-308.

212. Klepper J, Leiendecker B, Eltze C, Heussinger N: **Paroxysmal Nonepileptic Events in Glut1 Deficiency**. *Mov Disord Clin Pract* 2016, **3**(6):607-610.

213. Yu M, Miao J, Lv Y, Wang X, Zhang W, Shao N, Meng H: **A Challenging Diagnosis of Atypical Glut1-DS: A Case Report and Literature Review**. *Front Neurol* 2020, **11**:549331.

214. Coleman J, Dean M: **Longstanding Paroxysmal Dyskinesia in GLUT1 Deficiency Syndrome**. *Mov Disord Clin Pract* 2020, **7**(Suppl 3):S96-S98.

215. Scoppola C, Magli G, Conti M, Fadda M, Luzzu GM, Simula DM, Carta A, Sotgiu S, Casellato S: **CACNA1A-Linked Hemiplegic Migraine in GLUT 1 Deficiency Syndrome: A Case Report**. *Front Neurol* 2021, **12**:679354.

216. Madaan P, Jauhari P, Chakrabarty B, Gulati S: **Jeavons syndrome in a family with GLUT1-deficiency syndrome**. *Seizure* 2019, **71**:158-160.

217. Tornese G, Patti G, Pellegrin MC, Costa P, Faletra F, Faleschini E, Barbi E: **A case report of glucose transporter 1 deficiency syndrome with growth hormone deficiency diagnosed before starting ketogenic diet**. *Ital J Pediatr* 2020, **46**(1):119.

218. Angeli M, Vergadi E, Niotakis G, Raissaki M, Galanakis E: **Abnormal gait and hypoglycorrhachia in a toddler with seizures**. *Pediatr Investig* 2022, **6**(1):47-49.

219. Diaz J, Fonseca AG, Arboleda R, Frade A, Gennaro MP, Jayakar P, Schleifer P, Hernandez E: **Case Report: The Association of Wilson Disease in a Patient With Ataxia and GLUT-1 Deficiency**. *Front Pediatr* 2021, **9**:750593.

220. Yubero D, O'Callaghan M, Montero R, Ormazabal A, Armstrong J, Espinos C, Rodriguez MA, Jou C, Castejon E, Aracil MA *et al*: **Association between coenzyme Q10 and glucose transporter (GLUT1) deficiency**. *BMC Pediatr* 2014, **14**:284.

221. Thouin A, Crompton DE: **Glut1 deficiency syndrome: Absence epilepsy and La Soupe du Jour**. *Pract Neurol* 2016, **16**(1):50-52.

222. Chenouard A, Vuillaumier-Barrot S, Seta N, Kuster A: **A Cause of Permanent Ketosis: GLUT-1 Deficiency**. *JIMD Rep* 2015, **18**:79-83.

223. Hanci I, Kamm C, Scholten M, Roncoroni LP, Weber Y, Kruger R, Plewnia C, Gharabaghi A, Weiss D: **Long-Term Effect of GPi-DBS in a Patient With Generalized Dystonia Due to GLUT1 Deficiency Syndrome**. *Front Neurol* 2018, **9**:381.

224. Rotstein M, Doran J, Yang H, Ullner PM, Engelstad K, De Vivo DC: **Glut1 deficiency and alternating hemiplegia of childhood**. *Neurology* 2009, **73**(23):2042-2044.

225. Santarone ME, Piscitello LM, Volponi C, Vigevano F, Fusco L: **Focal non-motor seizures and subsequent focal motor seizures as the main clinical expression of GLUT-1 deficiency**. *Epilepsy Behav Rep* 2022, **20**:100571.

226. Graham JM, Jr.: **GLUT1 deficiency syndrome as a cause of encephalopathy that includes cognitive disability, treatment-resistant infantile epilepsy and a complex movement disorder**. *Eur J Med Genet* 2012, **55**(5):332-334.

227. Pascual JM, Ronen GM: **Glucose Transporter Type I Deficiency (G1D) at 25 (1990-2015): Presumptions, Facts, and the Lives of Persons With This Rare Disease**. *Pediatr Neurol* 2015, **53**(5):379-393.

228. Posar A, Santucci M: **Unusual phenotype of glucose transport protein type 1 deficiency syndrome: A case report and literature review**. *J Pediatr Neurosci* 2014, **9**(1):36-38.

229. Bawazir WM, Gevers EF, Flatt JF, Ang AL, Jacobs B, Oren C, Grunewald S, Dattani M, Bruce LJ, Stewart GW: **An infant with pseudohyperkalemia, hemolysis, and seizures: cation-leaky GLUT1-deficiency syndrome due to a SLC2A1 mutation**. *J Clin Endocrinol Metab* 2012, **97**(6):E987-993.

230. Leen WG, de Wit CJ, Wevers RA, van Engelen BG, Kamsteeg EJ, Klepper J, Verbeek MM, Willemsen MA: **Child neurology: differential diagnosis of a low CSF glucose in children and young adults**. *Neurology* 2013, **81**(24):e178-181.

231. Wang D, Yang H, Shi L, Ma L, Fujii T, Engelstad K, Pascual JM, De Vivo DC: **Functional studies of the T295M mutation causing Glut1 deficiency: glucose efflux preferentially affected by T295M**. *Pediatr Res* 2008, **64**(5):538-543.

232. Sen S, Keough K, Gibson J: **Clinical reasoning: novel GLUT1-DS mutation: refractory seizures and ataxia**. *Neurology* 2015, **84**(15):e111-114.

233. Colak R, Alkan Ozdemir S, Yangin Ergon E, Kagnici M, Calkavur S: **A Different SLC2A1 Gene Mutation in Glut 1 Deficiency Syndrome: c.734A>C**. *Balkan Med J* 2017, **34**(6):580-583.

234. Zanaboni MP, Pasca L, Geraci MA, Varesio C, Guglielmetti M, Tagliabue A, Grumi S, De Giorgis V: **Case report: KETOLAND the psychoeducation program for ketogenic diet**. *Front Psychiatry* 2023, **14**:1155717.

235. Lee HH, Hur YJ: **Glucose transport 1 deficiency presenting as infantile spasms with a mutation identified in exon 9 of SLC2A1**. *Korean J Pediatr* 2016, **59**(Suppl 1):S29-S31.

236. Panandikar GA, Ravat SH, Ansari RR, Desai KM: **Rare and Treatable Cause of Early-Onset Refractory Absence Seizures**. *J Pediatr Neurosci* 2018, **13**(3):358-361.

237. Menalled GS, Montero S, Faustinelli V, Poeta Casalis LDV, Crespo D, Colombo H: **[Hemidystonia and hemichorea in a pediatric patient with glucose transporter type 1 deficiency]**. *Arch Argent Pediatr* 2022, **120**(5):e207-e209.

238. Koy A, Assmann B, Klepper J, Mayatepek E: **Glucose transporter type 1 deficiency syndrome with carbohydrate-responsive symptoms but without epilepsy**. *Dev Med Child Neurol* 2011, **53**(12):1154-1156.

239. Pawlik W, Okulewicz P, Pawlik J, Krzywinska-Zdeb E: **Diagnostic and Clinical Manifestation Differences of Glucose Transporter Type 1 Deficiency Syndrome in a Family with SLC2A1 Gene Mutation**. *Int J Environ Res Public Health* 2022, **19**(6).

240. Good JM, Atallah I, Castro Jimenez M, Benninger D, Kuntzer T, Superti-Furga A, Tran C: **NGS-Based Diagnosis of Treatable Neurogenetic Disorders in Adults: Opportunities and Challenges**. *Genes (Basel)* 2021, **12**(5).

241. Hoshino H, Takayama K, Ishii A, Takahashi Y, Kanemura H: **Glucose transporter type 1 deficiency syndrome associated with autoantibodies to glutamate receptors**. *Brain Dev* 2020, **42**(9):686-690.

242. Algahtani H, Shirah B, Albarakaty A, Al-Qahtani MH, Abdulkareem AA, Naseer MI: **A Novel Intronic Variant in SLC2A1 Gene in a Saudi Patient with Myoclonic Epilepsy**. *J Epilepsy Res* 2020, **10**(1):40-43.

243. von Moers A, Brockmann K, Wang D, Korenke CG, Huppke P, De Vivo DC, Hanefeld F: **EEG features of glut-1 deficiency syndrome**. *Epilepsia* 2002, **43**(8):941-945.

244. Yu LF, Zhang YQ, Duan J, Ni Y, Gong XY, Lu ZY, Liao JX, Lu XP, Shi ZN, Lei MF *et al*: **[Clinical characteristics and ketogenic diet therapy of glucose transporter type 1 deficiency syndrome in children: a multicenter clinical study]**. *Zhonghua Er Ke Za Zhi* 2020, **58**(11):881-886.

245. Messana T, Russo A, Vergaro R, Boni A, Santucci M, Pini A: **Glucose Transporter Type 1 Deficiency Syndrome: Developmental Delay and Early-Onset Ataxia in a Novel Mutation of the SLC2A1 Gene**. *J Pediatr Neurosci* 2018, **13**(4):496-499.

246. Cornejo VE, Cabello JF, Colombo MC, Raimann EB: **[Glucose transponer type 1 deficiency sindrome (GLUT-1 SD) treated with ketogenic diet. Report of one case]**. *Rev Med Chil* 2007, **135**(5):631-635.

247. Wood AM, Geddes GC, Marashly A: **Language regression, hemichorea and focal subclinical seizures in a 6-year-old girl with GLUT-1 deficiency**. *Epilepsy Behav Rep* 2020, **14**:100340.

248. Roulet-Perez E, Ballhausen D, Bonafe L, Cronel-Ohayon S, Maeder-Ingvar M: **Glut-1 deficiency syndrome masquerading as idiopathic generalized epilepsy**. *Epilepsia* 2008, **49**(11):1955-1958.

249. Byrne S, Kearns J, Carolan R, Mc Menamin J, Klepper J, Webb D: **Refractory absence epilepsy associated with GLUT-1 deficiency syndrome**. *Epilepsia* 2011, **52**(5):1021-1024.

250. De Giorgis V, Ferraris C, Brena ML, Farris G, Gentilino V, Guglielmetti M, Marazzi C, Pasca L, Trentani C, Tagliabue A *et al*: **Classic ketogenic diet in parenteral nutrition in a GLUT1DS patient: Doing more with less in an acute surgical setting**. *Front Nutr* 2023, **10**:1114386.

251. Anand G, Padeniya A, Hanrahan D, Scheffer H, Zaiwalla Z, Cox D, Mann N, Hewertson J, Price S, Nemeth A *et al*: **Milder phenotypes of glucose transporter type 1 deficiency syndrome**. *Dev Med Child Neurol* 2011, **53**(7):664-668.

252. Leary LD, Wang D, Nordli DR, Jr., Engelstad K, De Vivo DC: **Seizure characterization and electroencephalographic features in Glut-1 deficiency syndrome**. *Epilepsia* 2003, **44**(5):701-707.

253. Gowda VK, Sheshu S: **Intermittent Ataxia with Early Onset Absence Epilepsy in Glucose Transporter Type 1 Deficiency Syndrome**. *Indian Pediatr* 2015, **52**(11):997.

254. Woo SB, Lee KH, Kang HC, Yang H, De Vivo DC, Kim SK: **First report of glucose transporter 1 deficiency syndrome in Korea with a novel splice site mutation**. *Gene* 2012, **506**(2):380-382.

255. Ismayilova N, Hacohen Y, MacKinnon AD, Elmslie F, Clarke A: **GLUT-1 deficiency presenting with seizures and reversible leukoencephalopathy on MRI imaging**. *Eur J Paediatr Neurol* 2018, **22**(6):1161-1164.

256. Bekker YAC, Lambrechts DA, Verhoeven JS, van Boxtel J, Troost C, Kamsteeg EJ, Willemsen MA, Braakman HMH: **Failure of ketogenic diet therapy in GLUT1 deficiency syndrome**. *Eur J Paediatr Neurol* 2019, **23**(3):404-409.

257. Gramer G, Wolf NI, Vater D, Bast T, Santer R, Kamsteeg EJ, Wevers RA, Ebinger F: **Glucose transporter-1 (GLUT1) deficiency syndrome: diagnosis and treatment in late childhood**. *Neuropediatrics* 2012, **43**(3):168-171.

258. Leen WG, Taher M, Verbeek MM, Kamsteeg EJ, van de Warrenburg BP, Willemsen MA: **GLUT1 deficiency syndrome into adulthood: a follow-up study**. *J Neurol* 2014, **261**(3):589-599.

259. Pellegrin S, Cantalupo G, Opri R, Dalla Bernardina B, Darra F: **EEG findings during "paroxysmal hemiplegia" in a patient with GLUT1-deficiency**. *Eur J Paediatr Neurol* 2017, **21**(3):580-582.

260. Diaz-Arias LA, Henry-Barron BJ, Buchholz A, Cervenka MC: **Positive impact of a modified Atkins diet on cognition, seizure control, and abnormal movements in an adult with glucose transporter type 1 deficiency syndrome: case report**. *Neurol Sci* 2022, **43**(5):3449-3452.

261. Klepper J, Scheffer H, Elsaid MF, Kamsteeg EJ, Leferink M, Ben-Omran T: **Autosomal recessive inheritance of GLUT1 deficiency syndrome**. *Neuropediatrics* 2009, **40**(5):207-210.

262. Hashimoto N, Kagitani-Shimono K, Sakai N, Otomo T, Tominaga K, Nabatame S, Mogami Y, Takahashi Y, Imai K, Yanagihara K *et al*: **SLC2A1 gene analysis of Japanese patients with glucose transporter 1 deficiency syndrome**. *J Hum Genet* 2011, **56**(12):846-851.

263. Vykuntaraju KN, Bhat S, Sanjay KS, Govindaraju M: **Symptomatic west syndrome secondary to glucose transporter-1(GLUT1) deficiency with complete response to 4:1 ketogenic diet**. *Indian J Pediatr* 2014, **81**(9):934-936.

264. Ustyol A, Takahashi S, Hatipoglu HU, Duman MA, Elevli M, Selcuk-Duru HN: **A novel mutation in SLC2A1 gene causing GLUT-1 deficiency syndrome in a young adult patient**. *Turk J Pediatr* 2019, **61**(6):946-948.

265. Haberlandt E, Karall D, Jud V, Baumgartner SS, Zotter S, Rostasy K, Baumann M, Scholl-Buergi S: **Glucose transporter type 1 deficiency syndrome effectively treated with modified Atkins diet**. *Neuropediatrics* 2014, **45**(2):117-119.

266. Gaspard N, Suls A, Vilain C, De Jonghe P, Van Bogaert P: **"Benign" myoclonic epilepsy of infancy as the initial presentation of glucose transporter-1 deficiency**. *Epileptic Disord* 2011, **13**(3):300-303.

267. Mohammad SS, Coman D, Calvert S: **Glucose transporter 1 deficiency syndrome and hemiplegic migraines as a dominant presenting clinical feature**. *J Paediatr Child Health* 2014, **50**(12):1025-1026.

268. Fazal A, Jose M, Rudrabhatla PK, Chandrasekharan SV, Sundaram S, Radhakrishnan A, Banerjee M, Menon RN: **Visual-sensitive epilepsy in GLUT-1 deficiency syndrome: Expanding the phenotype**. *Epileptic Disord* 2023, **25**(2):265-268.

269. Klepper J, Engelbrecht V, Scheffer H, van der Knaap MS, Fiedler A: **GLUT1 deficiency with delayed myelination responding to ketogenic diet**. *Pediatr Neurol* 2007, **37**(2):130-133.

270. Wang X, Li XY, Piao Y, Yuan G, Lin Y, Chen H, Wang Z, Li C, Wang C: **Hartnup disease presenting as hereditary spastic paraplegia and severe peripheral neuropathy**. *Am J Med Genet A* 2022, **188**(1):237-242.

271. Zhu Y, Chen L, He J, Chen Y, Gou H, Ma L, Qu Y, Liu Y, Wang D, Zhu Y: **Study of Seizure-Manifested Hartnup Disorder Case Induced By Novel Mutations in SLC6A19**. *Open Life Sci* 2018, **13**:22-27.

272. Martin-Lagos Maldonado A, Borrego Garcia E, Ruiz Sancho A: **A case report of hereditary coproporphyria with neurological, haematological and renal involvement**. *Gastroenterol Hepatol* 2019, **42**(7):438-439.

273. Lambie D, Florkowski C, Sies C, Raizis A, Siu WK, Towns C: **A case of hereditary coproporphyria with posterior reversible encephalopathy and novel coproporphyrinogen oxidase gene mutation c.863T>G (p.Leu288Trp)**. *Ann Clin Biochem* 2018, **55**(5):616-619.

274. Towns C, Mee H, McBride S: **Opioid dependence with successful transition to suboxone (buprenorphine/naloxone) in a young woman with hereditary coproporphyria**. *N Z Med J* 2020, **133**(1518):81-83.

275. Eroglu S, Birsenogul I: **Delirium with delayed diagnosis of hereditary coproporphyria**. *Clin Case Rep* 2022, **10**(6):e05937.

276. Baumgartner A, Quesenberry PJ: **A Unique Neuropsychiatric Syndrome in Variant Hereditary Coproporphyria: Case Report and Review of the Literature**. *J Hematol* 2017, **6**(1):21-24.

277. Grimes R, Gilleece Y, Appleby T, Stockwell S, Pinto-Sander N, Sahabandu T, Stein P, Bradshaw D: **A case of hereditary coproporphyria precipitated by efavirenz**. *AIDS* 2016, **30**(13):2142-2143.

278. Haimowitz S, Hsieh J, Shcherba M, Averbukh Y: **Liver failure after Hydroxycut use in a patient with undiagnosed hereditary coproporphyria**. *J Gen Intern Med* 2015, **30**(6):856-859.

279. Tran TP, Leduc K, Savard M, Dupre N, Rivest D, Nguyen DK: **Acute porphyria presenting as epilepsia partialis continua**. *Case Rep Neurol* 2013, **5**(2):116-124.

280. Jain G, Bennett JI, Resch DS, Godwin JE: **Schizoaffective disorder with missed diagnosis of acute porphyria: a case report and overview**. *Prim Care Companion CNS Disord* 2011, **13**(6).

281. Dahlgren M, Khosroshahi A, Stone JH: **A 22-year-old woman with severe headaches, vomiting, and tonic-clonic seizures**. *Arthritis Care Res (Hoboken)* 2011, **63**(1):165-171.

282. Bonkovsky HL, Maddukuri VC, Yazici C, Anderson KE, Bissell DM, Bloomer JR, Phillips JD, Naik H, Peter I, Baillargeon G *et al*: **Acute porphyrias in the USA: features of 108 subjects from porphyrias consortium**. *Am J Med* 2014, **127**(12):1233-1241.

283. Hasanoglu A, Balwani M, Kasapkara CS, Ezgu FS, Okur I, Tumer L, Cakmak A, Nazarenko I, Yu C, Clavero S *et al*: **Harderoporphyria due to homozygosity for coproporphyrinogen oxidase missense mutation H327R**. *J Inherit Metab Dis* 2011, **34**(1):225-231.

284. Liu A, Zhou L, Zhu H, Li Y, Yang J: **Systemic Lupus Erythematosus and Hereditary Coproporphyria: Two Different Entities Diagnosed by WES in the Same Patient**. *Biomed Res Int* 2022, **2022**:9096999.

285. Hasegawa K, Tanaka H, Yamashita M, Higuchi Y, Miyai T, Yoshimoto J, Okada A, Suzuki N, Iwatsuki K, Tsukahara H: **Neonatal-Onset Hereditary Coproporphyria: A New Variant of Hereditary Coproporphyria**. *JIMD Rep* 2017, **37**:99-106.

286. Henry SM, Stanfield MM: **Case Report of Hereditary Coproporphyria Accompanied by Nystagmus and Vestibular Dysfunction**; 2018.

287. To-Figueras J, Badenas C, Enriquez MT, Segura S, Alvarez C, Mila M, Lecha M, Herrero C: **Biochemical and genetic characterization of four cases of hereditary coproporphyria in Spain**. *Mol Genet Metab* 2005, **85**(2):160-163.

288. A. G, S. S: **P01-026 - A case of FMF and hereditary coprorphyria**. *Pediatr Rheumatol Online J* 2013, **11**:A30.

289. Paulsen JS, Langbehn DR, Stout JC, Aylward E, Ross CA, Nance M, Guttman M, Johnson S, MacDonald M, Beglinger LJ *et al*: **Detection of Huntington's disease decades before diagnosis: the Predict-HD study**. *J Neurol Neurosurg Psychiatry* 2008, **79**(8):874-880.

290. Kleyner R, Malcolmson J, Tegay D, Ward K, Maughan A, Maughan G, Nelson L, Wang K, Robison R, Lyon GJ: **KBG syndrome involving a single-nucleotide duplication in ANKRD11**. *Cold Spring Harb Mol Case Stud* 2016, **2**(6):a001131.

291. Murphy MJ, McSweeney N, Cavalleri GL, Greally MT, Benson KA, Costello DJ: **KBG syndrome mimicking genetic generalized epilepsy**. *Epilepsy Behav Rep* 2022, **19**:100545.

292. Libianto R, Wu KH, Devery S, Eisman JA, Center JR: **KBG syndrome presenting with brachydactyly type E**. *Bone* 2019, **123**:18-22.

293. Alves RM, Uva P, Veiga MF, Oppo M, Zschaber FCR, Porcu G, Porto HP, Persico I, Onano S, Cuccuru G *et al*: **Novel ANKRD11 gene mutation in an individual with a mild phenotype of KBG syndrome associated to a GEFS+ phenotypic spectrum: a case report**. *BMC Med Genet* 2019, **20**(1):16.

294. Schwaibold EM, Smogavec M, Hobbiebrunken E, Winter L, Zoll B, Burfeind P, Brockmann K, Pauli S: **Intragenic duplication of EHMT1 gene results in Kleefstra syndrome**. *Mol Cytogenet* 2014, **7**(1):74.

295. Nillesen WM, Yntema HG, Moscarda M, Verbeek NE, Wilson LC, Cowan F, Schepens M, Raas-Rothschild A, Gafni-Weinstein O, Zollino M *et al*: **Characterization of a novel transcript of the EHMT1 gene reveals important diagnostic implications for Kleefstra syndrome**. *Hum Mutat* 2011, **32**(7):853-859.

296. He X, Caluseriu O, Srivastava R, Denny AM, Bolduc FV: **Reversible white matter lesions associated with mutant EHMT1 and Kleefstra syndrome**. *Neurol Genet* 2016, **2**(2):e58.

297. Blackburn PR, Williams M, Cousin MA, Boczek NJ, Beek GJ, Lomberk GA, Urrutia RA, Babovic-Vuksanovic D, Klee EW: **A novel de novo frameshift deletion in EHMT1 in a patient with Kleefstra Syndrome results in decreased H3K9 dimethylation**. *Mol Genet Genomic Med* 2017, **5**(2):141-146.

298. Noruzinia M, Ahmadvand M, Bashti O, Salehi Chaleshtori AR: **Kleefstra Syndrome: The First Case Report From Iran**. *Acta Med Iran* 2017, **55**(10):650-654.

299. Rump A, Hildebrand L, Tzschach A, Ullmann R, Schrock E, Mitter D: **A mosaic maternal splice donor mutation in the EHMT1 gene leads to aberrant transcripts and to Kleefstra syndrome in the offspring**. *Eur J Hum Genet* 2013, **21**(8):887-890.

300. Bock I, Nemeth K, Pentelenyi K, Balicza P, Balazs A, Molnar MJ, Roman V, Nagy J, Levay G, Kobolak J *et al*: **Targeted next generation sequencing of a panel of autism-related genes identifies an EHMT1 mutation in a Kleefstra syndrome patient with autism and normal intellectual performance**. *Gene* 2016, **595**(2):131-141.

301. Verhoeven WM, Egger JI, Vermeulen K, van de Warrenburg BP, Kleefstra T: **Kleefstra syndrome in three adult patients: further delineation of the behavioral and neurological phenotype shows aspects of a neurodegenerative course**. *Am J Med Genet A* 2011, **155A**(10):2409-2415.

302. Kleefstra T, Brunner HG, Amiel J, Oudakker AR, Nillesen WM, Magee A, Genevieve D, Cormier-Daire V, van Esch H, Fryns JP *et al*: **Loss-of-function mutations in euchromatin histone methyl transferase 1 (EHMT1) cause the 9q34 subtelomeric deletion syndrome**. *Am J Hum Genet* 2006, **79**(2):370-377.

303. Forbes E, Smith K, Petluru M, Nystrom J, Fridman V: **Adult-onset Krabbe disease presenting as isolated sensorimotor demyelinating polyneuropathy: A case report**. *J Peripher Nerv Syst* 2022, **27**(4):320-324.

304. Zhang T, Yan C, Ji K, Lin P, Chi L, Zhao X, Zhao Y: **Adult-onset Krabbe disease in two generations of a Chinese family**. *Ann Transl Med* 2018, **6**(10):174.

305. Satoh JI, Tokumoto H, Kurohara K, Yukitake M, Matsui M, Kuroda Y, Yamamoto T, Furuya H, Shinnoh N, Kobayashi T *et al*: **Adult-onset Krabbe disease with homozygous T1853C mutation in the galactocerebrosidase gene. Unusual MRI findings of corticospinal tract demyelination**. *Neurology* 1997, **49**(5):1392-1399.

306. Xia Z, Wenwen Y, Xianfeng Y, Panpan H, Xiaoqun Z, Zhongwu S: **Adult-onset Krabbe disease due to a homozygous GALC mutation without abnormal signals on an MRI in a consanguineous family: A case report**. *Mol Genet Genomic Med* 2020, **8**(9):e1407.

307. Bajaj NP, Waldman A, Orrell R, Wood NW, Bhatia KP: **Familial adult onset of Krabbe's disease resembling hereditary spastic paraplegia with normal neuroimaging**. *J Neurol Neurosurg Psychiatry* 2002, **72**(5):635-638.

308. Zhuang S, Kong L, Li C, Chen L, Zhang T: **GALC mutations in Chinese patients with late-onset Krabbe disease: a case report**. *BMC Neurol* 2019, **19**(1):122.

309. Lim SM, Choi BO, Oh SI, Choi WJ, Oh KW, Nahm M, Xue Y, Choi JH, Choi JY, Kim YE *et al*: **Patient fibroblasts-derived induced neurons demonstrate autonomous neuronal defects in adult-onset Krabbe disease**. *Oncotarget* 2016, **7**(46):74496-74509.

310. Duraes J, Salsano E, Macario MDC: **Adult-Onset Krabbe Disease: The Importance of a Systematic Approach to Brain MRI Findings**. *Neurol Clin Pract* 2021, **11**(1):e15-e17.

311. Nutakki A, Jacobson R: **Adult-Onset Krabbe Disease Presenting as Demyelinating Neuropathy in a Much Older Adult: A Case Report (P10-13.010)**. *Neurology* 2022, **98**(18 Supplement):2346.

312. Prashanth LK, Murugan S, Kamath V, Gupta R, Jadav R, Sreekantaswamy S, Ramprasad VL: **First Report of Kufor-Rakeb Syndrome (PARK 9) from India, and a Novel Nonsense Mutation in ATP13A2 Gene**. *Mov Disord Clin Pract* 2015, **2**(3):326-327.

313. Rohani M, Lang AE, Sina F, Elahi E, Fasano A, Hardy J, Bras J, Alavi A: **Action Myoclonus and Seizure in Kufor-Rakeb Syndrome**. *Mov Disord Clin Pract* 2018, **5**(2):195-199.

314. Inzelberg R, Estrada-Cuzcano A, Laitman Y, De Vriendt E, Friedman E, Jordanova A: **Kufor-Rakeb Syndrome/PARK9: One Novel and One Possible Recurring Ashkenazi ATP13A2 Mutation**. *J Parkinsons Dis* 2018, **8**(3):399-403.

315. d'Orsi G, Lalla A, Palumbo O, Di Claudio MT, Valenzano A, Sabetta A, Lopopolo A, Muro ED, Palumbo P, Copetti M *et al*: **The presenting symptoms of Lafora Disease: An electroclinical and genetic study in five Apulian (Southern Italy) families**. *Seizure* 2020, **83**:145-153.

316. Arino H, Armangue T, Petit-Pedrol M, Sabater L, Martinez-Hernandez E, Hara M, Lancaster E, Saiz A, Dalmau J, Graus F: **Anti-LGI1-associated cognitive impairment: Presentation and long-term outcome**. *Neurology* 2016, **87**(8):759-765.

317. Muniz-Castrillo S, Haesebaert J, Thomas L, Vogrig A, Pinto AL, Picard G, Blanc C, Do LD, Joubert B, Berzero G *et al*: **Clinical and Prognostic Value of Immunogenetic Characteristics in Anti-LGI1 Encephalitis**. *Neurol Neuroimmunol Neuroinflamm* 2021, **8**(3).

318. Navarro V, Kas A, Apartis E, Chami L, Rogemond V, Levy P, Psimaras D, Habert MO, Baulac M, Delattre JY *et al*: **Motor cortex and hippocampus are the two main cortical targets in LGI1-antibody encephalitis**. *Brain* 2016, **139**(Pt 4):1079-1093.

319. Mickeviciute GC, Valiuskyte M, Platten M, Wszolek ZK, Andersen O, Danylaite Karrenbauer V, Ineichen BV, Granberg T: **Neuroimaging phenotypes of CSF1R-related leukoencephalopathy: Systematic review, meta-analysis, and imaging recommendations**. *J Intern Med* 2022, **291**(3):269-282.

320. Soni A, Meenawat A, Soni K, Srivastava V: **Lujan Fryns Syndrome - A Case Report**. *Clinical Practice* 2014, **3**(2):11-13.

321. Genis B, Sahin F, Cosar B: **Lujan-Fryns Syndrome Phenotype with Autism-Like Behavior and Atypical Psychotic Symptoms: Case Report**. *Turk Psikiyatri Derg* 2020, **31**(3):216-220.

322. Bellucco FT, de Mello CB, Meloni VA, Melaragno MI: **Malan syndrome in a patient with 19p13.2p13.12 deletion encompassing NFIX and CACNA1A genes: Case report and review of the literature**. *Mol Genet Genomic Med* 2019, **7**(12):e997.

323. Macchiaiolo M, Panfili FM, Vecchio D, Gonfiantini MV, Cortellessa F, Caciolo C, Zollino M, Accadia M, Seri M, Chinali M *et al*: **A deep phenotyping experience: up to date in management and diagnosis of Malan syndrome in a single center surveillance report**. *Orphanet J Rare Dis* 2022, **17**(1):235.

324. Alfieri P, Macchiaiolo M, Collotta M, Montanaro FAM, Caciolo C, Cumbo F, Galassi P, Panfili FM, Cortellessa F, Zollino M *et al*: **Characterization of Cognitive, Language and Adaptive Profiles of Children and Adolescents with Malan Syndrome**. *J Clin Med* 2022, **11**(14).

325. Mulder PA, van Balkom IDC, Landlust AM, Priolo M, Menke LA, Acero IH, Alkuraya FS, Arias P, Bernardini L, Bijlsma EK *et al*: **Development, behaviour and sensory processing in Marshall-Smith syndrome and Malan syndrome: phenotype comparison in two related syndromes**. *J Intellect Disabil Res* 2020, **64**(12):956-969.

326. Hancarova M, Havlovicova M, Putzova M, Vseticka J, Prchalova D, Stranecky V, Sedlacek Z: **Parental gonadal but not somatic mosaicism leading to de novo NFIX variants shared by two brothers with Malan syndrome**. *Am J Med Genet A* 2019, **179**(10):2119-2123.

327. Roll K: **The influence of regional health care structures on delay in diagnosis of rare diseases: the case of Marfan Syndrome**. *Health Policy* 2012, **105**(2-3):119-127.

328. Jendoubi F, Severino-Freire M, Negretto M, Arbus C, Paul C, Bulai Livideanu C: **Neuropsychiatric, cognitive and sexual impairment in mastocytosis patients**. *Orphanet J Rare Dis* 2021, **16**(1):118.

329. Chakravarty A, Bhattacharya P, Banerjee D, Mukherjee S: **Mcleod syndrome: Report of an Indian family with phenotypic heterogeneity**. *Ann Indian Acad Neurol* 2011, **14**(1):53-55.

330. Komiya H, Takasu M, Hashiguchi S, Uematsu E, Fukai R, Tanaka K, Tada M, Joki H, Takahashi T, Koyano S *et al*: **A Case of McLeod Syndrome with A Novel XK Missense Mutation**. *Mov Disord Clin Pract* 2018, **5**(3):333-336.

331. Ying Y, Yu S, Zhang J, He J, Xu X, Hong X, Zhu F: **A case of McLeod syndrome caused by a nonsense variation c.942G>A in the XK gene: A case report**. *Front Genet* 2023, **14**:1073139.

332. Xia S, Yu X, Song F, Sun B, Wang Y: **McLeod syndrome with a novel XK frameshift mutation: A case report**. *Medicine (Baltimore)* 2022, **101**(10):e28996.

333. Narumi S, Natori T, Miyazawa H, Kato T, Yonezawa H, Nishino I, Nakamura M, Sano A, Terayama Y: **Case of McLeod syndrome with a novel genetic mutation**. *Neurology and Clinical Neuroscience* 2016, **4**(3):115-117.

334. Yatsuga S, Povalko N, Nishioka J, Katayama K, Kakimoto N, Matsuishi T, Kakuma T, Koga Y, Taro Matsuoka for MSGiJ: **MELAS: a nationwide prospective cohort study of 96 patients in Japan**. *Biochim Biophys Acta* 2012, **1820**(5):619-624.

335. Anglin RE, Tarnopolsky MA, Mazurek MF, Rosebush PI: **The psychiatric presentation of mitochondrial disorders in adults**. *J Neuropsychiatry Clin Neurosci* 2012, **24**(4):394-409.

336. Alsalamah AK, Khan AO: **Asymptomatic retinal dysfunction in alpha-methylacyl-CoA racemase deficiency**. *Mol Vis* 2021, **27**:396-402.

337. Dick D, Horvath R, Chinnery PF: **AMACR mutations cause late-onset autosomal recessive cerebellar ataxia**. *Neurology* 2011, **76**(20):1768-1770.

338. Henao AI, Pira S, Herrera DA, Vargas SA, Montoya J, Castillo M: **Characteristic brain MRI findings in ataxia-neuropathy spectrum related to POLG mutation**. *Neuroradiol J* 2016, **29**(1):46-48.

339. Qiu J, Kumar KR, Watson E, Ahmad K, Sue CM, Hayes MW: **Dystonia Responsive to Dopamine: POLG Mutations Should Be Considered If Sensory Neuropathy Is Present**. *J Mov Disord* 2021, **14**(2):157-160.

340. Mongin M, Delorme C, Lenglet T, Jardel C, Vignal C, Roze E: **Progressive Ataxia and Palatal Tremor: Think about POLG Mutations**. *Tremor Other Hyperkinet Mov (N Y)* 2016, **6**:382.

341. Milone M, Brunetti-Pierri N, Tang LY, Kumar N, Mezei MM, Josephs K, Powell S, Simpson E, Wong LJ: **Sensory ataxic neuropathy with ophthalmoparesis caused by POLG mutations**. *Neuromuscul Disord* 2008, **18**(8):626-632.

342. Nuzhnyi E, Seliverstov Y, Klyushnikov S, Krylova T, Tsygankova P, Bychkov I, Zakharova E, Konovalov R, Fedin P, Abramycheva N *et al*: **POLG-associated ataxias can represent a substantial part of recessive and sporadic ataxias in adults**. *Clin Neurol Neurosurg* 2021, **201**:106462.

343. Marelli C, Lavigne C, Stepien KM, Janssen MCH, Feillet F, Kozich V, Jesina P, Schule R, Kessler C, Redonnet-Vernhet I *et al*: **Clinical and molecular characterization of adult patients with late-onset MTHFR deficiency**. *J Inherit Metab Dis* 2021, **44**(3):777-786.

344. Gales A, Masingue M, Millecamps S, Giraudier S, Grosliere L, Adam C, Salim C, Navarro V, Nadjar Y: **Adolescence/adult onset MTHFR deficiency may manifest as isolated and treatable distinct neuro-psychiatric syndromes**. *Orphanet J Rare Dis* 2018, **13**(1):29.

345. Kim MS, Yang A, Noh ES, Kim C, Bae GY, Lim HH, Park HD, Cho SY, Jin DK: **Natural History and Molecular Characteristics of Korean Patients with Mucopolysaccharidosis Type III**. *J Pers Med* 2022, **12**(5).

346. Martins C, de Medeiros PFV, Leistner-Segal S, Dridi L, Elcioglu N, Wood J, Behnam M, Noyan B, Lacerda L, Geraghty MT *et al*: **Molecular characterization of a large group of Mucopolysaccharidosis type IIIC patients reveals the evolutionary history of the disease**. *Hum Mutat* 2019, **40**(8):1084-1100.

347. Valstar MJ, Bertoli-Avella AM, Wessels MW, Ruijter GJ, de Graaf B, Olmer R, Elfferich P, Neijs S, Kariminejad R, Suheyl Ezgu F *et al*: **Mucopolysaccharidosis type IIID: 12 new patients and 15 novel mutations**. *Hum Mutat* 2010, **31**(5):E1348-1360.

348. Caputo V, Bocchinfuso G, Castori M, Traversa A, Pizzuti A, Stella L, Grammatico P, Tartaglia M: **Novel SMAD4 mutation causing Myhre syndrome**. *Am J Med Genet A* 2014, **164A**(7):1835-1840.

349. Picco P, Naselli A, Pala G, Marsciani A, Buoncompagni A, Martini A: **Recurrent pericarditis in Myhre syndrome**. *Am J Med Genet A* 2013, **161A**(5):1164-1166.

350. Artemios P, Areti S, Katerina P, Helen F, Eirini T, Charalambos P: **Autism Spectrum Disorder and Psychiatric Comorbidity in a Patient with Myhre Syndrome**. *J Autism Dev Disord* 2019, **49**(7):3031-3035.

351. Garcia-Cruz D, Figuera LE, Feria-Velazco A, Sanchez-Corona J, Garcia-Cruz MO, Ramirez-Duenas RM, Hernandez-Cordova A, Ruiz MX, Bitar-Alatorre WE, Ramirez-Duenas ML *et al*: **The Myhre syndrome: report of two cases**. *Clin Genet* 1993, **44**(4):203-207.

352. Ghosh D, Indulkar S: **Primary Myoclonus-Dystonia: A Diagnosis Often Missed in Children**. *J Child Neurol* 2013, **28**(11):1418-1422.

353. Jensen B, James R, Hong Y, Omoyinmi E, Pilkington C, Sebire NJ, Howell KJ, Brogan PA, Eleftheriou D: **A case of Myhre syndrome mimicking juvenile scleroderma**. *Pediatr Rheumatol Online J* 2020, **18**(1):72.

354. Cartagena A, Prasad AN, Rupar CA, Strong M, Tuchman M, Ah Mew N, Prasad C: **Recurrent encephalopathy: NAGS (N-acetylglutamate synthase) deficiency in adults**. *Can J Neurol Sci* 2013, **40**(1):3-9.

355. Hinnie J, Colombo JP, Wermuth B, Dryburgh FJ: **N-Acetylglutamate synthetase deficiency responding to carbamylglutamate**. *J Inherit Metab Dis* 1997, **20**(6):839-840.

356. Koseoglu E, Tepgec F, Yetkin MF, Uyguner O, Ekinci A, Abdulrezzak U, Hanagasi H: **Nasu Hakola Disease: A Rare Cause of Dementia and Cystic Bone Lesions, Report of a New Turkish Family**. *Noro Psikiyatr Ars* 2018, **55**(1):98-102.

357. Yamazaki K, Yoshino Y, Mori Y, Ochi S, Yoshida T, Ishimaru T, Ueno S: **A Case of Nasu-Hakola Disease without Fractures or Consanguinity Diagnosed Using Exome Sequencing and Treated with Sodium Valproate**. *Clin Psychopharmacol Neurosci* 2015, **13**(3):324-326.

358. Rashid Nezhad A, Olfati N, Shoeibi A, Rezaei Talab F, Soltani Sabi M: **Nasu-Hakola Disease With Stroke-like Attack: A Case Report**. *Alzheimer Dis Assoc Disord* 2023, **37**(2):168-170.

359. Kuroda R, Satoh J, Yamamura T, Anezaki T, Terada T, Yamazaki K, Obi T, Mizoguchi K: **A novel compound heterozygous mutation in the DAP12 gene in a patient with Nasu-Hakola disease**. *J Neurol Sci* 2007, **252**(1):88-91.

360. Chinnery PF, Crompton DE, Birchall D, Jackson MJ, Coulthard A, Lombes A, Quinn N, Wills A, Fletcher N, Mottershead JP *et al*: **Clinical features and natural history of neuroferritinopathy caused by the FTL1 460InsA mutation**. *Brain* 2007, **130**(Pt 1):110-119.

361. Smith KR, Dahl HH, Canafoglia L, Andermann E, Damiano J, Morbin M, Bruni AC, Giaccone G, Cossette P, Saftig P *et al*: **Cathepsin F mutations cause Type B Kufs disease, an adult-onset neuronal ceroid lipofuscinosis**. *Hum Mol Genet* 2013, **22**(7):1417-1423.

362. Ardicli D, Haliloglu G, Gocmen R, Gunbey C, Topcu M: **Unraveling neuronal ceroid lipofuscinosis type 2 (CLN2) disease: A tertiary center experience for determinants of diagnostic delay**. *Eur J Paediatr Neurol* 2021, **33**:94-98.

363. Wright GA, Georgiou M, Robson AG, Ali N, Kalhoro A, Holthaus SK, Pontikos N, Oluonye N, de Carvalho ER, Neveu MM *et al*: **Juvenile Batten Disease (CLN3): Detailed Ocular Phenotype, Novel Observations, Delayed Diagnosis, Masquerades, and Prospects for Therapy**. *Ophthalmol Retina* 2020, **4**(4):433-445.

364. Patterson MC, Mengel E, Wijburg FA, Muller A, Schwierin B, Drevon H, Vanier MT, Pineda M: **Disease and patient characteristics in NP-C patients: findings from an international disease registry**. *Orphanet J Rare Dis* 2013, **8**:12.

365. Stampfer M, Theiss S, Amraoui Y, Jiang X, Keller S, Ory DS, Mengel E, Fischer C, Runz H: **Niemann-Pick disease type C clinical database: cognitive and coordination deficits are early disease indicators**. *Orphanet J Rare Dis* 2013, **8**:35.

366. Sevin M, Lesca G, Baumann N, Millat G, Lyon-Caen O, Vanier MT, Sedel F: **The adult form of Niemann-Pick disease type C**. *Brain* 2007, **130**(Pt 1):120-133.

367. Kuseyri Hubschmann O, Julia-Palacios NA, Olivella M, Guder P, Zafeiriou DI, Horvath G, Kulhanek J, Pearson TS, Kuster A, Cortes-Saladelafont E *et al*: **Integrative Approach to Predict Severity in Nonketotic Hyperglycinemia**. *Ann Neurol* 2022, **92**(2):292-303.

368. Buerger C, Garbade SF, Dietrich Alber F, Waisbren SE, McCarter R, Kolker S, Burgard P, Urea Cycle Disorders C: **Impairment of cognitive function in ornithine transcarbamylase deficiency is global rather than domain-specific and is associated with disease onset, sex, maximum ammonium, and number of hyperammonemic events**. *J Inherit Metab Dis* 2019, **42**(2):243-253.

369. Marshall RD, Collins A, Escolar ML, Jinnah HA, Klopstock T, Kruer MC, Videnovic A, Robichaux-Viehoever A, Burns C, Swett LL *et al*: **Diagnostic and clinical experience of patients with pantothenate kinase-associated neurodegeneration**. *Orphanet J Rare Dis* 2019, **14**(1):174.

370. Boardman J, Mascareno Ponte M, Chaouch A, Kobylecki C: **Perry Syndrome with Intrafamilial Heterogeneity in Presentation and Survival Including Acute Respiratory Failure: Case Series**. *Mov Disord Clin Pract* 2022, **9**(6):816-820.

371. Umemoto G, Tsuboi Y, Furuya H, Mishima T, Fujioka S, Fujii N, Arahata H, Sugahara M, Sakai M: **Dysphagia in Perry Syndrome: Pharyngeal Pressure in Two Cases**. *Case Rep Neurol* 2017, **9**(2):161-167.

372. Stoker TB, Dostal V, Cochius J, Williams-Gray CH, Scherzer CR, Wang J, Liu G, Coyle-Gilchrist I: **DCTN1 mutation associated parkinsonism: case series of three new families with perry syndrome**. *J Neurol* 2022, **269**(12):6667-6672.

373. Nevado J, Garcia-Minaur S, Palomares-Bralo M, Vallespin E, Guillen-Navarro E, Rosell J, Bel-Fenellos C, Mori MA, Mila M, Del Campo M *et al*: **Variability in Phelan-McDermid Syndrome in a Cohort of 210 Individuals**. *Front Genet* 2022, **13**:652454.

374. Garcia-Bravo C, Palacios-Cena D, Garcia-Bravo S, Perez-Corrales J, Perez-de-Heredia-Torres M, Martinez-Piedrola RM: **Social and Family Challenges of Having a Child Diagnosed with Phelan-McDermid Syndrome: A Qualitative Study of Parents' Experiences**. *Int J Environ Res Public Health* 2022, **19**(17).

375. Akagi R, Kato N, Inoue R, Anderson KE, Jaffe EK, Sassa S: **delta-Aminolevulinate dehydratase (ALAD) porphyria: the first case in North America with two novel ALAD mutations**. *Mol Genet Metab* 2006, **87**(4):329-336.

376. Doss MO, Stauch T, Gross U, Renz M, Akagi R, Doss-Frank M, Seelig HP, Sassa S: **The third case of Doss porphyria (delta-amino-levulinic acid dehydratase deficiency) in Germany**. *J Inherit Metab Dis* 2004, **27**(4):529-536.

377. Bhadada SK, Bhansali A, Upreti V, Subbiah S, Khandelwal N: **Spectrum of neurological manifestations of idiopathic hypoparathyroidism and pseudohypoparathyroidism**. *Neurol India* 2011, **59**(4):586-589.

378. Sung AR, Moretti P, Shaibani A: **Case of late-onset Sandhoff disease due to a novel mutation in the HEXB gene**. *Neurol Genet* 2018, **4**(4):e260.

379. Gort L, de Olano N, Macias-Vidal J, Coll MA, Spanish GMWG: **GM2 gangliosidoses in Spain: analysis of the HEXA and HEXB genes in 34 Tay-Sachs and 14 Sandhoff patients**. *Gene* 2012, **506**(1):25-30.

380. Khani M, Shamshiri H, Moazzeni H, Taheri H, Ahmadieh H, Alavi A, Farboodi N, Nafissi S, Elahi E: **A case of adult onset Sandhoff disease that mimics Brown-Vialetto-Van Laere syndrome**. *Neuromuscul Disord* 2021, **31**(6):528-531.

381. Salamon A, Szpisjak L, Zadori D, Lenart I, Maroti Z, Kalmar T, Brierley CMH, Deegan PB, Klivenyi P: **Atypical presentation of late-onset Sandhoff disease: a case report**. *Ideggyogy Sz* 2021, **74**(11-12):425-429.

382. Gomez-Lira M, Sangalli A, Mottes M, Perusi C, Pignatti PF, Rizzuto N, Salviati A: **A common beta hexosaminidase gene mutation in adult Sandhoff disease patients**. *Hum Genet* 1995, **96**(4):417-422.

383. Maegawa GH, Stockley T, Tropak M, Banwell B, Blaser S, Kok F, Giugliani R, Mahuran D, Clarke JT: **The natural history of juvenile or subacute GM2 gangliosidosis: 21 new cases and literature review of 134 previously reported**. *Pediatrics* 2006, **118**(5):e1550-1562.

384. Kim W, Cho JS, Shim YK, Ko YJ, Choi SA, Kim SY, Kim H, Lim BC, Hwang H, Choi J *et al*: **Early-onset autosomal dominant GTP-cyclohydrolase I deficiency: Diagnostic delay and residual motor signs**. *Brain Dev* 2021, **43**(7):759-767.

385. Rive Le Gouard N, Jacquinet A, Ruaud L, Deleersnyder H, Ageorges F, Gallard J, Lacombe D, Odent S, Mikaty M, Manouvrier-Hanu S *et al*: **Smith-Magenis syndrome: Clinical and behavioral characteristics in a large retrospective cohort**. *Clin Genet* 2021, **99**(4):519-528.

386. Alsaffar H, Al Shidhani A, Zadjali A, Hameed Z, Ullah I, Al Maawali A: **De novo NSD1 mutation leading to Sotos syndrome – First case report from Oman**. *Journal of Diabetes and Endocrine Practice* 2022, **04**(04):202-205.

387. Shao Y-R, Dong Y, Du Y-C, Ma Y, Wu Z-Y: **[Preprint] Genetic and clinical profiles in a large cohort of Chinese individuals with spinocerebellar ataxia type 1**. 2022.

388. Giardina F, Lanza G, Cali F, Ferri R: **Late-onset oro-facial dyskinesia in Spinocerebellar Ataxia type 2: a case report**. *BMC Neurol* 2020, **20**(1):156.

389. Ramocki MB, Chapieski L, McDonald RO, Fernandez F, Malphrus AD: **Spinocerebellar ataxia type 2 presenting with cognitive regression in childhood**. *J Child Neurol* 2008, **23**(9):999-1001.

390. Almaguer-Mederos LE, Sarr L, Abascal JV, Aguilera-Rodriquez R, Martin MA, Khalil MI, Al-Jafari MA, de Jorge Lopez L, Volpini V, Nyan O: **Spinocerebellar ataxia type 2 in The Gambia: A case report**. *J Neurol Sci* 2015, **349**(1-2):269-271.

391. Singh RK, Sonkar KK, Bhoi S, Kalita J, Misra U: **Spinocerebellar ataxia type 2 associated with amyotrophic lateral sclerosis**. *Neurol India* 2017, **65**(5):1153-1155.

392. Bhalsing KS, Sowmya V, Netravathi M, Jain S, Pal PK: **Spinocerebellar Ataxia (SCA) type 2 presenting with chorea**. *Parkinsonism Relat Disord* 2013, **19**(12):1171-1172.

393. Gwinn-Hardy K, Singleton A, O'Suilleabhain P, Boss M, Nicholl D, Adam A, Hussey J, Critchley P, Hardy J, Farrer M: **Spinocerebellar ataxia type 3 phenotypically resembling parkinson disease in a black family**. *Arch Neurol* 2001, **58**(2):296-299.

394. Aminah S, Huda F, Gamayani U, Pusparini I, Mochyadin MFA, Sribudiani Y, Ibrahim NM, Achmad TH: **Clinical and genetic profile in index patients with spinocerebellar ataxia type 3 in Indonesia: case report**. *Heliyon* 2021, **7**(7):e07519.

395. Bettencourt C, Santos C, Coutinho P, Rizzu P, Vasconcelos J, Kay T, Cymbron T, Raposo M, Heutink P, Lima M: **Parkinsonian phenotype in Machado-Joseph disease (MJD/SCA3): a two-case report**. *BMC Neurol* 2011, **11**:131.

396. Son H, Yoon JG, Kim MJ, Moon J, Kim HJ: **First Cases of Spinocerebellar Ataxia 42 in Two Korean Families**. *J Mov Disord* 2023, **16**(1):110-113.

397. Iwata R, Inagawa T, Noda T, Takahashi Y, Kito S: **Spinocerebellar ataxia type 6 presenting with hallucination**. *Psychogeriatrics* 2021, **21**(3):446.

398. Lau KK, Au KM, Chen ML, Li HL, Sheng B, Chan AY: **Spinocerebellar ataxia type 6**. *Hong Kong Med J* 2005, **11**(3):207-209.

399. Chen Y, Li D, Wei M, Zhou M, Zhang L, Yu J, Qiu M, Jin Y, Lu X: **Homozygous spinocerebellar ataxia type 3 in China: a case report**. *J Int Med Res* 2021, **49**(6):3000605211021370.

400. Ikezawa J, Shimazaki R, Tobisawa S, Sugaya K, Takahashi K: **Dopa-responsive dystonia in spinocerebellar ataxia 6: A case report**. *Clin Neurol Neurosurg* 2023, **229**:107721.

401. Teive HA, Munhoz RP, Raskin S, Werneck LC: **Spinocerebellar ataxia type 6 in Brazil**. *Arq Neuropsiquiatr* 2008, **66**(3B):691-694.

402. Pedroso JL, de Carvalho Campos-Neto G, Speciali DS, Barsottini OG, Bor-Seng-Shu E, Felicio AC: **Spinocerebellar ataxia type 6 presenting with parkinsonism, pre-synaptic dopaminergic dysfunction and hyperechogenicity of the substantia nigra**. *J Neurol Sci* 2017, **376**:60-62.

403. Pearl PL, Gibson KM, Cortez MA, Wu Y, Carter Snead O, 3rd, Knerr I, Forester K, Pettiford JM, Jakobs C, Theodore WH: **Succinic semialdehyde dehydrogenase deficiency: lessons from mice and men**. *J Inherit Metab Dis* 2009, **32**(3):343-352.

404. Wright D, Kenny A, Eley S, McKechanie AG, Stanfield AC: **Clinical and behavioural features of SYNGAP1-related intellectual disability: a parent and caregiver description**. *J Neurodev Disord* 2022, **14**(1):34.

405. Gupta S, Hwang Y, Ludwig N, Henry J, Kadam SD: **Case report: Off-label use of low-dose perampanel in a 25-month-old girl with a pathogenic SYNGAP1 variant**. *Front Neurol* 2023, **14**:1221161.

406. Prchalova D, Havlovicova M, Sterbova K, Stranecky V, Hancarova M, Sedlacek Z: **Analysis of 31-year-old patient with SYNGAP1 gene defect points to importance of variants in broader splice regions and reveals developmental trajectory of SYNGAP1-associated phenotype: case report**. *BMC Med Genet* 2017, **18**(1):62.

407. Kimura Y, Akahira-Azuma M, Harada N, Enomoto Y, Tsurusaki Y, Kurosawa K: **Novel SYNGAP1 variant in a patient with intellectual disability and distinctive dysmorphisms**. *Congenit Anom (Kyoto)* 2018, **58**(6):188-190.

408. Writzl K, Knegt AC: **6p21.3 microdeletion involving the SYNGAP1 gene in a patient with intellectual disability, seizures, and severe speech impairment**. *Am J Med Genet A* 2013, **161A**(7):1682-1685.

409. Lee CG, Jang JH, Seo JY: **First identified Korean family with Tatton-Brown-Rahman Syndrome caused by the novel DNMT3A variant c.118G>C p.(Glu40Gln)**. *Ann Pediatr Endocrinol Metab* 2019, **24**(4):253-256.

410. Lennartsson O, Lodefalk M, Wehtje H, Stattin EL, Savendahl L, Nilsson O: **Case Report: Bilateral Epiphysiodesis Due to Extreme Tall Stature in a Girl With a De Novo DNMT3A Variant Associated With Tatton-Brown-Rahman Syndrome**. *Front Endocrinol (Lausanne)* 2021, **12**:752756.

411. Tenorio J, Alarcon P, Arias P, Dapia I, Garcia-Minaur S, Palomares Bralo M, Campistol J, Climent S, Valenzuela I, Ramos S *et al*: **Further delineation of neuropsychiatric findings in Tatton-Brown-Rahman syndrome due to disease-causing variants in DNMT3A: seven new patients**. *Eur J Hum Genet* 2020, **28**(4):469-479.

412. Balci TB, Strong A, Kalish JM, Zackai E, Maris JM, Reilly A, Surrey LF, Wertheim GB, Marcadier JL, Graham GE *et al*: **Tatton-Brown-Rahman syndrome: Six individuals with novel features**. *Am J Med Genet A* 2020, **182**(4):673-680.

413. Yokoi T, Enomoto Y, Naruto T, Kurosawa K, Higurashi N: **Tatton-Brown-Rahman syndrome with a novel DNMT3A mutation presented severe intellectual disability and autism spectrum disorder**. *Hum Genome Var* 2020, **7**:15.

414. Paz-Alegria MC, Gomez-Forero D, Osorio-Patino J, Jaramillo-Echeverry A: **Behavioral and dental management of a patient with Tatton-Brown-Rahman syndrome: Case report**. *Spec Care Dentist* 2020, **40**(6):597-604.

415. **Our Members** [https://tbrsyndrome.org/profiles]

416. Neudorfer O, Pastores GM, Zeng BJ, Gianutsos J, Zaroff CM, Kolodny EH: **Late-onset Tay-Sachs disease: phenotypic characterization and genotypic correlations in 21 affected patients**. *Genet Med* 2005, **7**(2):119-123.

417. Jahnova H, Poupetova H, Jireckova J, Vlaskova H, Kostalova E, Mazanec R, Zumrova A, Mecir P, Musova Z, Magner M: **Amyotrophy, cerebellar impairment and psychiatric disease are the main symptoms in a cohort of 14 Czech patients with the late-onset form of Tay-Sachs disease**. *J Neurol* 2019, **266**(8):1953-1959.

418. Kingswood JC, d'Augeres GB, Belousova E, Ferreira JC, Carter T, Castellana R, Cottin V, Curatolo P, Dahlin M, de Vries PJ *et al*: **TuberOus SClerosis registry to increase disease Awareness (TOSCA) - baseline data on 2093 patients**. *Orphanet J Rare Dis* 2017, **12**(1):2.

419. Schiava M, Ikenaga C, Villar-Quiles RN, Caballero-Avila M, Topf A, Nishino I, Kimonis V, Udd B, Schoser B, Zanoteli E *et al*: **Genotype-phenotype correlations in valosin-containing protein disease: a retrospective muticentre study**. *J Neurol Neurosurg Psychiatry* 2022.

420. Accogli A, Brais B, Tampieri D, La Piana R: **Long-Standing Psychiatric Features as the Only Clinical Presentation of Vanishing White Matter Disease**. *J Neuropsychiatry Clin Neurosci* 2019, **31**(3):276-279.

421. Ashrafi MR, Rezaei Z, Heidari M, Nikbakht S, Malamiri RA, Mohammadi M, Zamani GR, Badv RS, Rostami P, Movahedinia M *et al*: **The First Report of Relative Incidence of Inherited White Matter Disorders in an Asian Country Based on an Iranian Bioregistry System**. *J Child Neurol* 2018, **33**(4):255-259.

422. Klingelhoefer L, Misbahuddin A, Jawad T, Mellers J, Jarosz J, Weeks R, Ray Chaudhuri K: **Vanishing white matter disease presenting as opsoclonus myoclonus syndrome in childhood--a case report and review of the literature**. *Pediatr Neurol* 2014, **51**(1):157-164.

423. Nagdev G, Vhora RS, Chavan G, Sahu G: **Adult Onset Vanishing White Matter Disease: A Rare Case Report**. *Cureus* 2022, **14**(10):e30177.

424. Parihar J, Vibha D, Rajan R, Pandit AK, Srivastava AK, Prasad K: **Vanishing White Matter Disease Presenting as Dementia and Infertility: A Case Report**. *Neurol Genet* 2022, **8**(3):e643.

425. Ghezzi L, Scarpini E, Rango M, Arighi A, Bassi MT, Tenderini E, De Riz M, Jacini F, Fumagalli GG, Pietroboni AM *et al*: **A 66-year-old patient with vanishing white matter disease due to the p.Ala87Val EIF2B3 mutation**. *Neurology* 2012, **79**(20):2077-2078.

426. Wei C, Qin Q, Chen F, Zhou A, Wang F, Zuo X, Chen R, Lyu J, Jia J: **Adult-onset vanishing white matter disease with the EIF2B2 gene mutation presenting as menometrorrhagia**. *BMC Neurol* 2019, **19**(1):203.

427. Damasio J, van der Lei HD, van der Knaap MS, Santos E: **Late onset vanishing white matter disease presenting with learning difficulties**. *J Neurol Sci* 2012, **314**(1-2):169-170.

428. Agorastos A, Huber CG: **Vanishing white-matter disease: a case of severe adult onset with prolonged course under anticonvulsive therapy**. *J Neuropsychiatry Clin Neurosci* 2012, **24**(4):E24-25.

429. Ibitoye RT, Renowden SA, Faulkner HJ, Scolding NJ, Rice CM: **Ovarioleukodystrophy due to EIF2B5 mutations**. *Pract Neurol* 2016, **16**(6):496-499.

430. S. B, S.D. B, I. B, M. O, S.M. M: **Adult-onset vanishing white matter disease presenting as dementia**. *Annals of Alzheimer's and Dementia Care* 2021, **5**(1):4-8.

431. Vafaee-Shahi M, Ghasemi S, Riahi A, Sadr Z: **A boy with blistering of sun-exposed skin and finger shortening: the first case of Variegate Porphyria with a novel mutation in protoporphyrinogen oxidase (PPOX) gene in Iran: a case report and literature review**. *Ital J Pediatr* 2022, **48**(1):27.

432. Collaud E, Wittwer L, Minder AE, Annoni JM, Minder EI, Chabwine JN: **Case Report: Variegate porphyria disclosed by post-gastric bypass complications and causing predominant painful sensorimotor axonal peripheral neuropathy**. *Front Genet* 2022, **13**:993453.

433. Tsuchiya Y, Hoshino J, Suwabe T, Sumida K, Hiramatsu R, Mise K, Hasegawa E, Yamanouchi M, Hayami N, Sawa N *et al*: **Variegate porphyria complicated by systemic AA amyloidosis: a case report**. *Amyloid* 2013, **20**(4):272-274.

434. Shen M-H, Chi M-H, Kuo T-T: **New heterozygous mutation of protoporphyrinogen oxidase gene in a case of variegate porphyria in Taiwan**. *Dermatologica Sinica* 2020, **38**(3):196-197.

435. Duan J, Ye Y, Liao J, Chen L, Zhao X, Liu C, Wen J: **White-Sutton syndrome and congenital heart disease: case report and literature review**. *BMC Pediatr* 2023, **23**(1):158.

436. Murch O, Jain V, Benneche A, Metcalfe K, Hobson E, Prescott K, Chandler K, Ghali N, Carmichael J, Foulds NC *et al*: **Further delineation of the clinical spectrum of White-Sutton syndrome: 12 new individuals and a review of the literature**. *Eur J Hum Genet* 2022, **30**(1):95-100.

437. Assia Batzir N, Posey JE, Song X, Akdemir ZC, Rosenfeld JA, Brown CW, Chen E, Holtrop SG, Mizerik E, Nieto Moreno M *et al*: **Phenotypic expansion of POGZ-related intellectual disability syndrome (White-Sutton syndrome)**. *Am J Med Genet A* 2020, **182**(1):38-52.

438. Liu S, Yan Z, Huang Y, Zheng W, Deng Y, Zou Y, Xie H: **A case of White-Sutton syndrome arising from a maternally-inherited mutation in POGZ**. *Psychiatr Genet* 2021, **31**(4):135-139.

439. Donnarumma B, Riccio MP, Terrone G, Palma M, Strisciuglio P, Scala I: **Expanding the neurological and behavioral phenotype of White-Sutton syndrome: a case report**. *Ital J Pediatr* 2021, **47**(1):148.

440. Merriweather A, Murdock DR, Rosenfeld JA, Dai H, Ketkar S, Emrick L, Nicholas S, Lewis RA, Undiagnosed Diseases N, Bacino CA *et al*: **A novel, de novo intronic variant in POGZ causes White-Sutton syndrome**. *Am J Med Genet A* 2022, **188**(7):2198-2203.

441. Trimarchi G, Caraffi SG, Radio FC, Barresi S, Contro G, Pizzi S, Maini I, Pollazzon M, Fusco C, Sassi S *et al*: **Adducted Thumb and Peripheral Polyneuropathy: Diagnostic Supports in Suspecting White-Sutton Syndrome: Case Report and Review of the Literature**. *Genes (Basel)* 2021, **12**(7).

442. Giraldo-Ocampo S, Pacheco-Orozco RA, Pachajoa H: **A Novel POGZ Variant in a Patient with Intellectual Disability and Obesity**. *Appl Clin Genet* 2022, **15**:63-68.

443. White J, Beck CR, Harel T, Posey JE, Jhangiani SN, Tang S, Farwell KD, Powis Z, Mendelsohn NJ, Baker JA *et al*: **POGZ truncating alleles cause syndromic intellectual disability**. *Genome Med* 2016, **8**(1):3.

444. Villalba MF, Chang TC: **Congenital corneal opacities as a new feature in an unusual case of White-Sutton syndrome**. *J AAPOS* 2022, **26**(5):265-268.

445. Ferretti A, Barresi S, Trivisano M, Ciolfi A, Dentici ML, Radio FC, Vigevano F, Tartaglia M, Specchio N: **POGZ-related epilepsy: Case report and review of the literature**. *Am J Med Genet A* 2019, **179**(8):1631-1636.

446. Tan B, Zou Y, Zhang Y, Zhang R, Ou J, Shen Y, Zhao J, Luo X, Guo J, Zeng L *et al*: **A novel de novo POGZ mutation in a patient with intellectual disability**. *J Hum Genet* 2016, **61**(4):357-359.

447. Ye Y, Cho MT, Retterer K, Alexander N, Ben-Omran T, Al-Mureikhi M, Cristian I, Wheeler PG, Crain C, Zand D *et al*: **De novo POGZ mutations are associated with neurodevelopmental disorders and microcephaly**. *Cold Spring Harb Mol Case Stud* 2015, **1**(1):a000455.

448. Merle U, Schaefer M, Ferenci P, Stremmel W: **Clinical presentation, diagnosis and long-term outcome of Wilson's disease: a cohort study**. *Gut* 2007, **56**(1):115-120.

449. Zimbrean PC, Schilsky ML: **Psychiatric aspects of Wilson disease: a review**. *Gen Hosp Psychiatry* 2014, **36**(1):53-62.

450. Bueno GE, Ruiz-Castaneda D, Martinez JR, Munoz MR, Alascio PC: **Natural history and clinical characteristics of 50 patients with Wolfram syndrome**. *Endocrine* 2018, **61**(3):440-446.

451. Zmyslowska A, Borowiec M, Fichna P, Iwaniszewska B, Majkowska L, Pietrzak I, Szalecki M, Szypowska A, Mlynarski W: **Delayed recognition of Wolfram syndrome frequently misdiagnosed as type 1 diabetes with early chronic complications**. *Exp Clin Endocrinol Diabetes* 2014, **122**(1):35-38.

452. Hdiji O, Turki E, Bouzidi N, Bouchhima I, Damak M, Bohlega S, Mhiri C: **Woodhouse-Sakati Syndrome: Report of the First Tunisian Family with the C2orf37 Gene Mutation**. *J Mov Disord* 2016, **9**(2):120-123.

453. Almeqdadi M, Kemppainen JL, Pichurin PN, Gavrilova RH: **Phenotypic Variability of c.436delC DCAF17 Gene Mutation in Woodhouse-Sakati Syndrome**. *Am J Case Rep* 2018, **19**:347-353.

454. Nanda A, Pasternack SM, Mahmoudi H, Ishorst N, Grimalt R, Betz RC: **Alopecia and hypotrichosis as characteristic findings in Woodhouse-Sakati syndrome: report of a family with mutation in the C2orf37 gene**. *Pediatr Dermatol* 2014, **31**(1):83-87.

455. Ben-Omran T, Ali R, Almureikhi M, Alameer S, Al-Saffar M, Walsh CA, Felie JM, Teebi A: **Phenotypic heterogeneity in Woodhouse-Sakati syndrome: two new families with a mutation in the C2orf37 gene**. *Am J Med Genet A* 2011, **155A**(11):2647-2653.

456. Sheridan MB, Wohler E, Batista DA, Applegate C, Hoover-Fong J: **The Use of High-Density SNP Array to Map Homozygosity in Consanguineous Families to Efficiently Identify Candidate Genes: Application to Woodhouse-Sakati Syndrome**. *Case Rep Genet* 2015, **2015**:169482.

457. Alderson J, Ghosh PS: **Clinical Reasoning: Seven-year-old girl with progressive gait difficulties**. *Neurology* 2020, **94**(8):364-367.

458. Habib R, Basit S, Khan S, Khan MN, Ahmad W: **A novel splice site mutation in gene C2orf37 underlying Woodhouse-Sakati syndrome (WSS) in a consanguineous family of Pakistani origin**. *Gene* 2011, **490**(1-2):26-31.

459. Haeri G, Akhoundi FH, Alavi A, Abdi S, Rohani M: **Endocrine Abnormalities in a Case of Neurodegeneration with Brain Iron Accumulation**. *Mov Disord Clin Pract* 2020, **7**(6):706-707.

460. Chen G, Zhou L, Chen Q, Wang J, Jiang P, Shen R, Long M, Zhou H: **Case Report: A Deletion Variant in the DCAF17 Gene Underlying Woodhouse-Sakati Syndrome in a Chinese Consanguineous Family**. *Front Genet* 2021, **12**:741323.

461. Abdulla MC, Alazami AM, Alungal J, Koya JM, Musambil M: **Novel compound heterozygous frameshift mutations of C2orf37 in a familial Indian case of Woodhouse-Sakati syndrome**. *J Genet* 2015, **94**(3):489-492.

462. Miyajima H, Kohno S, Takahashi Y, Yonekawa O, Kanno T: **Estimation of the gene frequency of aceruloplasminemia in Japan**. *Neurology* 1999, **53**(3):617-619.

463. Elder G, Harper P, Badminton M, Sandberg S, Deybach JC: **The incidence of inherited porphyrias in Europe**. *J Inherit Metab Dis* 2013, **36**(5):849-857.

464. Gitiaux C, Ceballos-Picot I, Marie S, Valayannopoulos V, Rio M, Verrieres S, Benoist JF, Vincent MF, Desguerre I, Bahi-Buisson N: **Misleading behavioural phenotype with adenylosuccinate lyase deficiency**. *Eur J Hum Genet* 2009, **17**(1):133-136.

465. Helsmoortel C, Vulto-van Silfhout AT, Coe BP, Vandeweyer G, Rooms L, van den Ende J, Schuurs-Hoeijmakers JH, Marcelis CL, Willemsen MH, Vissers LE *et al*: **A SWI/SNF-related autism syndrome caused by de novo mutations in ADNP**. *Nat Genet* 2014, **46**(4):380-384.

466. Wiens K, Berry SA, Choi H, Gaviglio A, Gupta A, Hietala A, Kenney-Jung D, Lund T, Miller W, Pierpont EI *et al*: **A report on state-wide implementation of newborn screening for X-linked Adrenoleukodystrophy**. *Am J Med Genet A* 2019, **179**(7):1205-1213.

467. Yoshida T, Sasaki M, Yoshida M, Namekawa M, Okamoto Y, Tsujino S, Sasayama H, Mizuta I, Nakagawa M, Alexander Disease Study Group in J: **Nationwide survey of Alexander disease in Japan and proposed new guidelines for diagnosis**. *J Neurol* 2011, **258**(11):1998-2008.

468. Meikle PJ, Ranieri E, Simonsen H, Rozaklis T, Ramsay SL, Whitfield PD, Fuller M, Christensen E, Skovby F, Hopwood JJ: **Newborn screening for lysosomal storage disorders: clinical evaluation of a two-tier strategy**. *Pediatrics* 2004, **114**(4):909-916.

469. Pinto R, Caseiro C, Lemos M, Lopes L, Fontes A, Ribeiro H, Pinto E, Silva E, Rocha S, Marcao A *et al*: **Prevalence of lysosomal storage diseases in Portugal**. *Eur J Hum Genet* 2004, **12**(2):87-92.

470. Schindler D, Desnick RJ: **Schindler disease: deficient α-N-acetylgalactosaminidase activity**, 6th edn: Academic Press; 2020.

471. Kloth K, Denecke J, Hempel M, Johannsen J, Strom TM, Kubisch C, Lessel D: **First de novo ANK3 nonsense mutation in a boy with intellectual disability, speech impairment and autistic features**. *Eur J Med Genet* 2017, **60**(9):494-498.

472. Brusilow SW, Maestri NE: **Urea cycle disorders: diagnosis, pathophysiology, and therapy**. *Adv Pediatr* 1996, **43**:127-170.

473. Summar ML, Koelker S, Freedenberg D, Le Mons C, Haberle J, Lee HS, Kirmse B, European R, Network for Intoxication Type Metabolic Diseases . Electronic address hwe-ioeip, Members of the Urea Cycle Disorders Consortium . Electronic address hreueu: **The incidence of urea cycle disorders**. *Mol Genet Metab* 2013, **110**(1-2):179-180.

474. Hult M, Darin N, von Dobeln U, Mansson JE: **Epidemiology of lysosomal storage diseases in Sweden**. *Acta Paediatr* 2014, **103**(12):1258-1263.

475. Arvio M, Mononen I: **Aspartylglycosaminuria: a review**. *Orphanet J Rare Dis* 2016, **11**(1):162.

476. Raininko R, Gosky M, Padiath QS: **LMNB1-Related Autosomal Dominant Leukodystrophy**. In: *GeneReviews((R)).* Edited by Adam MP, Mirzaa GM, Pagon RA, Wallace SE, Bean LJH, Gripp KW, Amemiya A. Seattle (WA); 1993.

477. Balasubramanian M, Willoughby J, Fry AE, Weber A, Firth HV, Deshpande C, Berg JN, Chandler K, Metcalfe KA, Lam W *et al*: **Delineating the phenotypic spectrum of Bainbridge-Ropers syndrome: 12 new patients with de novo, heterozygous, loss-of-function mutations in ASXL3 and review of published literature**. *J Med Genet* 2017, **54**(8):537-543.

478. Schirwani S, Albaba S, Carere DA, Guillen Sacoto MJ, Milan Zamora F, Si Y, Rabin R, Pappas J, Renaud DL, Hauser N *et al*: **Expanding the phenotype of ASXL3-related syndrome: A comprehensive description of 45 unpublished individuals with inherited and de novo pathogenic variants in ASXL3**. *Am J Med Genet A* 2021, **185**(11):3446-3458.

479. Prah A, Pregeljc D, Stare J, Mavri J: **Brunner syndrome caused by point mutation explained by multiscale simulation of enzyme reaction**. *Sci Rep* 2022, **12**(1):21889.

480. Razvi SS, Davidson R, Bone I, Muir KW: **The prevalence of cerebral autosomal dominant arteriopathy with subcortical infarcts and leucoencephalopathy (CADASIL) in the west of Scotland**. *J Neurol Neurosurg Psychiatry* 2005, **76**(5):739-741.

481. Kalimo H, Ruchoux MM, Viitanen M, Kalaria RN: **CADASIL: a common form of hereditary arteriopathy causing brain infarcts and dementia**. *Brain Pathol* 2002, **12**(3):371-384.

482. Fukutake T: **Cerebral autosomal recessive arteriopathy with subcortical infarcts and leukoencephalopathy (CARASIL): from discovery to gene identification**. *J Stroke Cerebrovasc Dis* 2011, **20**(2):85-93.

483. van de Kamp JM, Betsalel OT, Mercimek-Mahmutoglu S, Abulhoul L, Grunewald S, Anselm I, Azzouz H, Bratkovic D, de Brouwer A, Hamel B *et al*: **Phenotype and genotype in 101 males with X-linked creatine transporter deficiency**. *J Med Genet* 2013, **50**(7):463-472.

484. Mercimek-Mahmutoglu S, Pop A, Kanhai W, Fernandez Ojeda M, Holwerda U, Smith D, Loeber JG, Schielen PC, Salomons GS: **A pilot study to estimate incidence of guanidinoacetate methyltransferase deficiency in newborns by direct sequencing of the GAMT gene**. *Gene* 2016, **575**(1):127-131.

485. Lorincz MT, Rainier S, Thomas D, Fink JK: **Cerebrotendinous xanthomatosis: possible higher prevalence than previously recognized**. *Arch Neurol* 2005, **62**(9):1459-1463.

486. Blake KD, Prasad C: **CHARGE syndrome**. *Orphanet J Rare Dis* 2006, **1**:34.

487. Peikert K, Dobson-Stone C, Rampoldi L, Miltenberger-Miltenyi G, Neiman A, De Camilli P, Hermann A, Walker RH, Monaco AP, Danek A: **VPS13A Disease**. In: *GeneReviews((R)).* Edited by Adam MP, Mirzaa GM, Pagon RA, Wallace SE, Bean LJH, Gripp KW, Amemiya A. Seattle (WA); 1993.

488. Jung HH, Danek A, Walker RH: **Neuroacanthocytosis syndromes**. *Orphanet J Rare Dis* 2011, **6**:68.

489. Nagata N, Matsuda I, Oyanagi K: **Estimated frequency of urea cycle enzymopathies in Japan**. *Am J Med Genet* 1991, **39**(2):228-229.

490. Pereira PM, Schneider A, Pannetier S, Heron D, Hanauer A: **Coffin-Lowry syndrome**. *Eur J Hum Genet* 2010, **18**(6):627-633.

491. Skovby F, Gaustadnes M, Mudd SH: **A revisit to the natural history of homocystinuria due to cystathionine beta-synthase deficiency**. *Mol Genet Metab* 2010, **99**(1):1-3.

492. Munro CS: **The phenotype of Darier's disease: penetrance and expressivity in adults and children**. *Br J Dermatol* 1992, **127**(2):126-130.

493. Tsuji S, Onodera O, Goto J, Nishizawa M, Study Group on Ataxic D: **Sporadic ataxias in Japan--a population-based epidemiological study**. *Cerebellum* 2008, **7**(2):189-197.

494. Demmler JC, Atkinson MD, Reinhold EJ, Choy E, Lyons RA, Brophy ST: **Diagnosed prevalence of Ehlers-Danlos syndrome and hypermobility spectrum disorder in Wales, UK: a national electronic cohort study and case-control comparison**. *BMJ Open* 2019, **9**(11):e031365.

495. Miklovic T, Sieg VC: **Ehlers-Danlos Syndrome**. In: *StatPearls.* Treasure Island (FL); 2024.

496. Saleem S, Aslam HM, Anwar M, Anwar S, Saleem M, Saleem A, Rehmani MA: **Fahr's syndrome: literature review of current evidence**. *Orphanet J Rare Dis* 2013, **8**:156.

497. Thomsen LL, Eriksen MK, Roemer SF, Andersen I, Olesen J, Russell MB: **A population-based study of familial hemiplegic migraine suggests revised diagnostic criteria**. *Brain* 2002, **125**(Pt 6):1379-1391.

498. Lopez-Rivera JA, Perez-Palma E, Symonds J, Lindy AS, McKnight DA, Leu C, Zuberi S, Brunklaus A, Moller RS, Lal D: **A catalogue of new incidence estimates of monogenic neurodevelopmental disorders caused by de novo variants**. *Brain* 2020, **143**(4):1099-1105.

499. Levesque S, Dombrowski C, Morel ML, Rehel R, Cote JS, Bussieres J, Morgan K, Rousseau F: **Screening and instability of FMR1 alleles in a prospective sample of 24,449 mother-newborn pairs from the general population**. *Clin Genet* 2009, **76**(6):511-523.

500. Coffee B, Keith K, Albizua I, Malone T, Mowrey J, Sherman SL, Warren ST: **Incidence of fragile X syndrome by newborn screening for methylated FMR1 DNA**. *Am J Hum Genet* 2009, **85**(4):503-514.

501. Youings SA, Murray A, Dennis N, Ennis S, Lewis C, McKechnie N, Pound M, Sharrock A, Jacobs P: **FRAXA and FRAXE: the results of a five year survey**. *J Med Genet* 2000, **37**(6):415-421.

502. Bidichandani SI, Delatycki MB: **Friedreich Ataxia**. In: *GeneReviews((R)).* Edited by Adam MP, Mirzaa GM, Pagon RA, Wallace SE, Bean LJH, Gripp KW, Amemiya A. Seattle (WA); 1993.

503. Logroscino G, Piccininni M, Graff C, Hardiman O, Ludolph AC, Moreno F, Otto M, Remes AM, Rowe JB, Seelaar H *et al*: **Incidence of Syndromes Associated With Frontotemporal Lobar Degeneration in 9 European Countries**. *JAMA Neurol* 2023, **80**(3):279-286.

504. Stirnemann J, Vigan M, Hamroun D, Heraoui D, Rossi-Semerano L, Berger MG, Rose C, Camou F, de Roux-Serratrice C, Grosbois B *et al*: **The French Gaucher's disease registry: clinical characteristics, complications and treatment of 562 patients**. *Orphanet J Rare Dis* 2012, **7**:77.

505. Symonds JD, Zuberi SM, Stewart K, McLellan A, O'Regan M, MacLeod S, Jollands A, Joss S, Kirkpatrick M, Brunklaus A *et al*: **Incidence and phenotypes of childhood-onset genetic epilepsies: a prospective population-based national cohort**. *Brain* 2019, **142**(8):2303-2318.

506. Atwal PS, Medina CR, Burrage LC, Sutton VR: **Nineteen-year follow-up of a patient with severe glutathione synthetase deficiency**. *J Hum Genet* 2016, **61**(7):669-672.

507. **The Metabolic and Molecular Bases of Inherited Disease (Scriver, C. R., Beaudet, A. L., Sly, W. S., Valle, D., Childs, B., Kinzler, K. W., and Vogelstein, B., eds., 8th ed., McGraw-Hill, New-York, 2001, 7012 p., $550.00)**. *Biochemistry (Moscow)* 2002, **67**(5):611-612.

508. Wilcken B, Yu JS, Brown DA: **Natural history of Hartnup disease**. *Arch Dis Child* 1977, **52**(1):38-40.

509. Pillai NR, Yubero D, Shayota BJ, Oyarzabal A, Ghosh R, Sun Q, Azamian MS, Arjona C, Brandi N, Palau F *et al*: **Loss of CLTRN function produces a neuropsychiatric disorder and a biochemical phenotype that mimics Hartnup disease**. *Am J Med Genet A* 2019, **179**(12):2459-2468.

510. Azmanov DN, Rodgers H, Auray-Blais C, Giguere R, Bailey C, Broer S, Rasko JE, Cavanaugh JA: **Persistence of the common Hartnup disease D173N allele in populations of European origin**. *Ann Hum Genet* 2007, **71**(Pt 6):755-761.

511. Medina A, Mahjoub Y, Shaver L, Pringsheim T: **Prevalence and Incidence of Huntington's Disease: An Updated Systematic Review and Meta-Analysis**. *Mov Disord* 2022, **37**(12):2327-2335.

512. Morel Swols D, Foster J, 2nd, Tekin M: **KBG syndrome**. *Orphanet J Rare Dis* 2017, **12**(1):183.

513. Sirmaci A, Spiliopoulos M, Brancati F, Powell E, Duman D, Abrams A, Bademci G, Agolini E, Guo S, Konuk B *et al*: **Mutations in ANKRD11 cause KBG syndrome, characterized by intellectual disability, skeletal malformations, and macrodontia**. *Am J Hum Genet* 2011, **89**(2):289-294.

514. Willemsen MH, Vulto-van Silfhout AT, Nillesen WM, Wissink-Lindhout WM, van Bokhoven H, Philip N, Berry-Kravis EM, Kini U, van Ravenswaaij-Arts CM, Delle Chiaie B *et al*: **Update on Kleefstra Syndrome**. *Mol Syndromol* 2012, **2**(3-5):202-212.

515. Orsini JJ, Escolar ML, Wasserstein MP, Caggana M: **Krabbe Disease**. In: *GeneReviews((R)).* Edited by Adam MP, Mirzaa GM, Pagon RA, Wallace SE, Bean LJH, Gripp KW, Amemiya A. Seattle (WA); 1993.

516. Santoro L, Breedveld GJ, Manganelli F, Iodice R, Pisciotta C, Nolano M, Punzo F, Quarantelli M, Pappata S, Di Fonzo A *et al*: **Novel ATP13A2 (PARK9) homozygous mutation in a family with marked phenotype variability**. *Neurogenetics* 2011, **12**(1):33-39.

517. Riva A, Orsini A, Scala M, Taramasso V, Canafoglia L, d'Orsi G, Di Claudio MT, Avolio C, D'Aniello A, Elia M *et al*: **Italian cohort of Lafora disease: Clinical features, disease evolution, and genotype-phenotype correlations**. *J Neurol Sci* 2021, **424**:117409.

518. Munoz-Sanchez G, Planaguma J, Naranjo L, Couso R, Sabater L, Guasp M, Martinez-Hernandez E, Graus F, Dalmau J, Ruiz-Garcia R: **The diagnosis of anti-LGI1 encephalitis varies with the type of immunodetection assay and sample examined**. *Front Immunol* 2022, **13**:1069368.

519. Sassi C, Nalls MA, Ridge PG, Gibbs JR, Lupton MK, Troakes C, Lunnon K, Al-Sarraj S, Brown KS, Medway C *et al*: **Mendelian adult-onset leukodystrophy genes in Alzheimer's disease: critical influence of CSF1R and NOTCH3**. *Neurobiol Aging* 2018, **66**:179 e117-179 e129.

520. Van Buggenhout G, Fryns JP: **Lujan-Fryns syndrome (mental retardation, X-linked, marfanoid habitus)**. *Orphanet J Rare Dis* 2006, **1**:26.

521. Khan A, Humayun M, Haider I, Ayub M: **Lujan-Fryns Syndrome (LFS): A Unique Combination of Hypernasality, Marfanoid Body Habitus, and Neuropsychiatric Issues, Presenting as Acute-Onset Dysphagia**. *Clin Med Insights Case Rep* 2016, **9**:115-118.

522. Pyeritz RE: **The Marfan syndrome**. *Am Fam Physician* 1986, **34**(6):83-94.

523. Brockow K: **Epidemiology, prognosis, and risk factors in mastocytosis**. *Immunol Allergy Clin North Am* 2014, **34**(2):283-295.

524. Walker RH, Miranda M, Jung HH, Danek A: **Life expectancy and mortality in chorea-acanthocytosis and McLeod syndrome**. *Parkinsonism Relat Disord* 2019, **60**:158-161.

525. Majamaa K, Turkka J, Karppa M, Winqvist S, Hassinen IE: **The common MELAS mutation A3243G in mitochondrial DNA among young patients with an occipital brain infarct**. *Neurology* 1997, **49**(5):1331-1334.

526. Lugowska A, Poninska J, Krajewski P, Broda G, Ploski R: **Population carrier rates of pathogenic ARSA gene mutations: is metachromatic leukodystrophy underdiagnosed?** *PLoS One* 2011, **6**(6):e20218.

527. Waters PJ, Thuriot F, Clarke JT, Gravel S, Watkins D, Rosenblatt DS, Levesque S: **Methylmalonyl-coA epimerase deficiency: A new case, with an acute metabolic presentation and an intronic splicing mutation in the MCEE gene**. *Mol Genet Metab Rep* 2016, **9**:19-24.

528. Hakonen AH, Heiskanen S, Juvonen V, Lappalainen I, Luoma PT, Rantamaki M, Goethem GV, Lofgren A, Hackman P, Paetau A *et al*: **Mitochondrial DNA polymerase W748S mutation: a common cause of autosomal recessive ataxia with ancient European origin**. *Am J Hum Genet* 2005, **77**(3):430-441.

529. Barretta F, Uomo F, Fecarotta S, Albano L, Crisci D, Verde A, Fisco MG, Gallo G, Dottore Stagna D, Pricolo MR *et al*: **Contribution of Genetic Test to Early Diagnosis of Methylenetetrahydrofolate Reductase (MTHFR) Deficiency: The Experience of a Reference Center in Southern Italy**. *Genes (Basel)* 2023, **14**(5).

530. Malm G, Lund AM, Mansson JE, Heiberg A: **Mucopolysaccharidoses in the Scandinavian countries: incidence and prevalence**. *Acta Paediatr* 2008, **97**(11):1577-1581.

531. Poorthuis BJ, Wevers RA, Kleijer WJ, Groener JE, de Jong JG, van Weely S, Niezen-Koning KE, van Diggelen OP: **The frequency of lysosomal storage diseases in The Netherlands**. *Hum Genet* 1999, **105**(1-2):151-156.

532. Puckett Y, Mallorga-Hernandez A, Montano AM: **Epidemiology of mucopolysaccharidoses (MPS) in United States: challenges and opportunities**. *Orphanet J Rare Dis* 2021, **16**(1):241.

533. Lin AE, Brunetti-Pierri N, Lindsay ME, Schimmenti LA, Starr LJ: **Myhre Syndrome**. In: *GeneReviews((R)).* Edited by Adam MP, Mirzaa GM, Pagon RA, Wallace SE, Bean LJH, Gripp KW, Amemiya A. Seattle (WA); 1993.

534. Caviness JN, Alving LI, Maraganore DM, Black RA, McDonnell SK, Rocca WA: **The incidence and prevalence of myoclonus in Olmsted County, Minnesota**. *Mayo Clin Proc* 1999, **74**(6):565-569.

535. Kenneson A, Singh RH: **Presentation and management of N-acetylglutamate synthase deficiency: a review of the literature**. *Orphanet J Rare Dis* 2020, **15**(1):279.

536. Pekkarinen P, Hovatta I, Hakola P, Jarvi O, Kestila M, Lenkkeri U, Adolfsson R, Holmgren G, Nylander PO, Tranebjaerg L *et al*: **Assignment of the locus for PLO-SL, a frontal-lobe dementia with bone cysts, to 19q13**. *Am J Hum Genet* 1998, **62**(2):362-372.

537. Kumar N, Rizek P, Jog M: **Neuroferritinopathy: Pathophysiology, Presentation, Differential Diagnoses and Management**. *Tremor Other Hyperkinet Mov (N Y)* 2016, **6**:355.

538. Santavuori P: **Neuronal ceroid-lipofuscinoses in childhood**. *Brain Dev* 1988, **10**(2):80-83.

539. Jalanko A, Braulke T: **Neuronal ceroid lipofuscinoses**. *Biochim Biophys Acta* 2009, **1793**(4):697-709.

540. Vanier MT: **Niemann-Pick disease type C**. *Orphanet J Rare Dis* 2010, **5**:16.

541. Applegarth DA, Toone JR, Lowry RB: **Incidence of inborn errors of metabolism in British Columbia, 1969-1996**. *Pediatrics* 2000, **105**(1):e10.

542. Coughlin CR, 2nd, Swanson MA, Kronquist K, Acquaviva C, Hutchin T, Rodriguez-Pombo P, Vaisanen ML, Spector E, Creadon-Swindell G, Bras-Goldberg AM *et al*: **The genetic basis of classic nonketotic hyperglycinemia due to mutations in GLDC and AMT**. *Genet Med* 2017, **19**(1):104-111.

543. Dulski J, Konno T, Wszolek Z: **DCTN1-Related Neurodegeneration**. In: *GeneReviews((R)).* Edited by Adam MP, Mirzaa GM, Pagon RA, Wallace SE, Bean LJH, Gripp KW, Amemiya A. Seattle (WA); 1993.

544. Abrahams BS, Geschwind DH: **Advances in autism genetics: on the threshold of a new neurobiology**. *Nat Rev Genet* 2008, **9**(5):341-355.

545. Mohan G, Madan A: **Ala Dehydratase Deficiency Porphyria**. In: *StatPearls.* Treasure Island (FL); 2023.

546. Burd L, Vesley B, Martsolf JT, Kerbeshian J: **Prevalence study of Rett syndrome in North Dakota children**. *Am J Med Genet* 1991, **38**(4):565-568.

547. Hagberg B, Hanefeld F, Percy A, Skjeldal O: **An update on clinically applicable diagnostic criteria in Rett syndrome. Comments to Rett Syndrome Clinical Criteria Consensus Panel Satellite to European Paediatric Neurology Society Meeting, Baden Baden, Germany, 11 September 2001**. *Eur J Paediatr Neurol* 2002, **6**(5):293-297.

548. Kozinetz CA, Skender ML, MacNaughton N, Almes MJ, Schultz RJ, Percy AK, Glaze DG: **Epidemiology of Rett syndrome: a population-based registry**. *Pediatrics* 1993, **91**(2):445-450.

549. Cantor RM, Roy C, Lim JS, Kaback MM: **Sandhoff disease heterozygote detection: a component of population screening for Tay-Sachs disease carriers. II. Sandhoff disease gene frequencies in American Jewish and non-Jewish populations**. *Am J Hum Genet* 1987, **41**(1):16-26.

550. Nygaard TG: **Dopa-responsive dystonia. Delineation of the clinical syndrome and clues to pathogenesis**. *Adv Neurol* 1993, **60**:577-585.

551. Greenberg F, Guzzetta V, Montes de Oca-Luna R, Magenis RE, Smith AC, Richter SF, Kondo I, Dobyns WB, Patel PI, Lupski JR: **Molecular analysis of the Smith-Magenis syndrome: a possible contiguous-gene syndrome associated with del(17)(p11.2)**. *Am J Hum Genet* 1991, **49**(6):1207-1218.

552. Smith AC, Magenis RE, Elsea SH: **Overview of Smith-Magenis syndrome**. *J Assoc Genet Technol* 2005, **31**(4):163-167.

553. Tatton-Brown K, Cole TRP, Rahman N: **Sotos Syndrome**. In: *GeneReviews((R)).* Edited by Adam MP, Feldman J, Mirzaa GM, Pagon RA, Wallace SE, Bean LJH, Gripp KW, Amemiya A. Seattle (WA); 1993.

554. van Prooije T, Ibrahim NM, Azmin S, van de Warrenburg B: **Spinocerebellar ataxias in Asia: Prevalence, phenotypes and management**. *Parkinsonism Relat Disord* 2021, **92**:112-118.

555. Ruano L, Melo C, Silva MC, Coutinho P: **The global epidemiology of hereditary ataxia and spastic paraplegia: a systematic review of prevalence studies**. *Neuroepidemiology* 2014, **42**(3):174-183.

556. Ngo K, Aker M, Petty LE, Chen J, Cavalcanti F, Nelson AB, Hassin-Baer S, Geschwind MD, Perlman S, Italiano D *et al*: **Expanding the global prevalence of spinocerebellar ataxia type 42**. *Neurol Genet* 2018, **4**(3):e232.

557. Bargiela D, Yu-Wai-Man P, Keogh M, Horvath R, Chinnery PF: **Prevalence of neurogenetic disorders in the North of England**. *Neurology* 2015, **85**(14):1195-1201.

558. Pearl PL, Wiwattanadittakul N, Roullet JB, Gibson KM: **Succinic Semialdehyde Dehydrogenase Deficiency**. In: *GeneReviews((R)).* Edited by Adam MP, Mirzaa GM, Pagon RA, Wallace SE, Bean LJH, Gripp KW, Amemiya A. Seattle (WA); 1993.

559. Martin K, McConnell A, Elsea SH: **Assessing Prevalence and Carrier Frequency of Succinic Semialdehyde Dehydrogenase Deficiency**. *J Child Neurol* 2021, **36**(13-14):1218-1222.

560. Berryer MH, Hamdan FF, Klitten LL, Moller RS, Carmant L, Schwartzentruber J, Patry L, Dobrzeniecka S, Rochefort D, Neugnot-Cerioli M *et al*: **Mutations in SYNGAP1 cause intellectual disability, autism, and a specific form of epilepsy by inducing haploinsufficiency**. *Hum Mutat* 2013, **34**(2):385-394.

561. Carvill GL, Heavin SB, Yendle SC, McMahon JM, O'Roak BJ, Cook J, Khan A, Dorschner MO, Weaver M, Calvert S *et al*: **Targeted resequencing in epileptic encephalopathies identifies de novo mutations in CHD2 and SYNGAP1**. *Nat Genet* 2013, **45**(7):825-830.

562. Deciphering Developmental Disorders S: **Large-scale discovery of novel genetic causes of developmental disorders**. *Nature* 2015, **519**(7542):223-228.

563. Tatton-Brown K, Seal S, Ruark E, Harmer J, Ramsay E, Del Vecchio Duarte S, Zachariou A, Hanks S, O'Brien E, Aksglaede L *et al*: **Mutations in the DNA methyltransferase gene DNMT3A cause an overgrowth syndrome with intellectual disability**. *Nat Genet* 2014, **46**(4):385-388.

564. Kosaki R, Terashima H, Kubota M, Kosaki K: **Acute myeloid leukemia-associated DNMT3A p.Arg882His mutation in a patient with Tatton-Brown-Rahman overgrowth syndrome as a constitutional mutation**. *Am J Med Genet A* 2017, **173**(1):250-253.

565. Ebrahimi-Fakhari D, Mann LL, Poryo M, Graf N, von Kries R, Heinrich B, Ebrahimi-Fakhari D, Flotats-Bastardas M, Gortner L, Zemlin M *et al*: **Incidence of tuberous sclerosis and age at first diagnosis: new data and emerging trends from a national, prospective surveillance study**. *Orphanet J Rare Dis* 2018, **13**(1):117.

566. Ikenaga C, Findlay AR, Seiffert M, Peck A, Peck N, Johnson NE, Statland JM, Weihl CC: **Phenotypic diversity in an international Cure VCP Disease registry**. *Orphanet J Rare Dis* 2020, **15**(1):267.

567. Musumano LB, Fancello V, Negossi L, Ballardini E, Bigoni S, Ciorba A: **Auditory Neuropathy Spectrum Disorder in the White Sutton Syndrome**. *J Int Adv Otol* 2023, **19**(3):260-262.

568. Nagy D, Verheyen S, Wigby KM, Borovikov A, Sharkov A, Slegesky V, Larson A, Fagerberg C, Brasch-Andersen C, Kibaek M *et al*: **Genotype-Phenotype Comparison in POGZ-Related Neurodevelopmental Disorders by Using Clinical Scoring**. *Genes (Basel)* 2022, **13**(1).

569. Sandahl TD, Laursen TL, Munk DE, Vilstrup H, Weiss KH, Ott P: **The Prevalence of Wilson's Disease: An Update**. *Hepatology* 2020, **71**(2):722-732.

570. Barrett TG, Bundey SE, Macleod AF: **Neurodegeneration and diabetes: UK nationwide study of Wolfram (DIDMOAD) syndrome**. *Lancet* 1995, **346**(8988):1458-1463.

571. Schneider SA, Bhatia KP: **Dystonia in the Woodhouse Sakati syndrome: A new family and literature review**. *Mov Disord* 2008, **23**(4):592-596.

572. Abouelhoda M, Sobahy T, El-Kalioby M, Patel N, Shamseldin H, Monies D, Al-Tassan N, Ramzan K, Imtiaz F, Shaheen R *et al*: **Clinical genomics can facilitate countrywide estimation of autosomal recessive disease burden**. *Genet Med* 2016, **18**(12):1244-1249.

573. Tang Y, Qin Q, Xing Y, Guo D, Di L, Jia J: **AARS2 leukoencephalopathy: A new variant of mitochondrial encephalomyopathy**. *Mol Genet Genomic Med* 2019, **7**(4):e00582.

574. Wu C, Wang M, Wang X, Li W, Li S, Chen B, Niu S, Tai H, Pan H, Zhang Z: **The genetic and phenotypic spectra of adult genetic leukoencephalopathies in a cohort of 309 patients**. *Brain* 2023, **146**(6):2364-2376.

575. Miyajima H, Hosoi Y: **Aceruloplasminemia**. In: *GeneReviews((R)).* Edited by Adam MP, Feldman J, Mirzaa GM, Pagon RA, Wallace SE, Bean LJH, Gripp KW, Amemiya A. Seattle (WA); 1993.

576. Spiritos Z, Salvador S, Mosquera D, Wilder J: **Acute Intermittent Porphyria: Current Perspectives And Case Presentation**. *Ther Clin Risk Manag* 2019, **15**:1443-1451.

577. Narang N, Banerjee A, Kotwal J, Kaur J, Sharma YV, Sharma CS: **Psychiatric Manifestations in three cases of Acute Intermittent Porphyria**. *Med J Armed Forces India* 2003, **59**(2):171-173.

578. Altintoprak AE, Ersel M, Bayrakci A: **An unusual suicide attempt: a case with psychosis during an acute porphyric attack**. *Eur J Emerg Med* 2009, **16**(2):106-108.

579. Duque-Serrano L, Patarroyo-Rodriguez L, Gotlib D, Molano-Eslava JC: **Psychiatric Aspects of Acute Porphyria: a Comprehensive Review**. *Curr Psychiatry Rep* 2018, **20**(1):5.

580. Jurecka A, Zikanova M, Kmoch S, Tylki-Szymanska A: **Adenylosuccinate lyase deficiency**. *J Inherit Metab Dis* 2015, **38**(2):231-242.

581. Jaeken J, Van den Berghe G: **An infantile autistic syndrome characterised by the presence of succinylpurines in body fluids**. *Lancet* 1984, **2**(8411):1058-1061.

582. Van den Berghe G, Vincent MF, Jaeken J: **Inborn errors of the purine nucleotide cycle: adenylosuccinase deficiency**. *J Inherit Metab Dis* 1997, **20**(2):193-202.

583. Stathis SL, Cowley DM, Broe D: **Autism and adenylosuccinase deficiency**. *J Am Acad Child Adolesc Psychiatry* 2000, **39**(3):274-275.

584. Fastman J, Kolevzon A: **ADNP Syndrome: A Qualitative Assessment of Symptoms, Therapies, and Challenges**. *Children (Basel)* 2023, **10**(3).

585. Kolevzon A, Levy T, Barkley S, Bedrosian-Sermone S, Davis M, Foss-Feig J, Halpern D, Keller K, Kostic A, Layton C *et al*: **An open-label study evaluating the safety, behavioral, and electrophysiological outcomes of low-dose ketamine in children with ADNP syndrome**. *HGG Adv* 2022, **3**(4):100138.

586. Garside S, Rosebush PI, Levinson AJ, Mazurek MF: **Late-onset adrenoleukodystrophy associated with long-standing psychiatric symptoms**. *J Clin Psychiatry* 1999, **60**(7):460-468.

587. Galvao ACR, Machado-Porto GCL, Porto FHG, Lucato LT, Nitrini R: **Adult-onset adrenoleukodystrophy presenting as a psychiatric disorder: MRI findings**. *Dement Neuropsychol* 2012, **6**(4):290-295.

588. Walterfang MA, O'Donovan J, Fahey MC, Velakoulis D: **The neuropsychiatry of adrenomyeloneuropathy**. *CNS Spectr* 2007, **12**(9):696-701.

589. Lichtenstein ML, Dwosh E, Roy Chowdhury A, Farrer MJ, McKenzie MB, Guella I, Evans DM, Nygaard HB, Shewchuk JR, Hayden S *et al*: **Neurobehavioral characterization of adult-onset Alexander disease: A family study**. *Neurol Clin Pract* 2017, **7**(5):425-429.

590. Arshiany H, Ezzatian B, Artounian V, Alizadeh F, Mohammadian F: **Psychiatric Onset Alexander Disease: An Important Challenge in Neuropsychiatric Diagnosis: A Case Report**. *Basic Clin Neurosci* 2022, **13**(2):269-274.

591. Beck M, Olsen KJ, Wraith JE, Zeman J, Michalski JC, Saftig P, Fogh J, Malm D: **Natural history of alpha mannosidosis a longitudinal study**. *Orphanet J Rare Dis* 2013, **8**:88.

592. Malm D, Pantel J, Linaker OM: **Psychiatric symptoms in alpha-mannosidosis**. *J Intellect Disabil Res* 2005, **49**(Pt 11):865-871.

593. Adam J, Malone R, Lloyd S, Lee J, Hendriksz CJ, Ramaswami U: **Disease progression of alpha-mannosidosis and impact on patients and carers - A UK natural history survey**. *Mol Genet Metab Rep* 2019, **20**:100480.

594. Blanchon YC, Gay C, Gibert G, Lauras B: **A case of N-acetyl galactosaminidase deficiency (Schindler disease) associated with autism**. *J Autism Dev Disord* 2002, **32**(2):145-146.

595. Bin Sawad A, Jackimiec J, Bechter M, Trucillo A, Lindsley K, Bhagat A, Uyei J, Diaz GA: **Epidemiology, methods of diagnosis, and clinical management of patients with arginase 1 deficiency (ARG1-D): A systematic review**. *Mol Genet Metab* 2022, **137**(1-2):153-163.

596. Elkhateeb N, Olivieri G, Siri B, Boyd S, Stepien KM, Sharma R, Morris AAM, Hartley T, Crowther L, Grunewald S *et al*: **Natural history of epilepsy in argininosuccinic aciduria provides new insights into pathophysiology: A retrospective international study**. *Epilepsia* 2023, **64**(6):1612-1626.

597. Leuger L, Dieu X, Chao de la Barca JM, Moriconi M, Halley G, Donin de Rosiere X, Reynier P, Mirebeau-Prunier D, Homedan C: **Late-onset argininosuccinic aciduria in a 72-year-old man presenting with fatal hyperammonemia**. *JIMD Rep* 2021, **62**(1):44-48.

598. Nagamani SCS, Erez A, Lee B: **Argininosuccinate Lyase Deficiency**. In: *GeneReviews((R)).* Edited by Adam MP, Mirzaa GM, Pagon RA, Wallace SE, Bean LJH, Gripp KW, Amemiya A. Seattle (WA); 1993.

599. Widhalm K, Koch S, Scheibenreiter S, Knoll E, Colombo JP, Bachmann C, Thalhammer O: **Long-term follow-up of 12 patients with the late-onset variant of argininosuccinic acid lyase deficiency: no impairment of intellectual and psychomotor development during therapy**. *Pediatrics* 1992, **89**(6 Pt 2):1182-1184.

600. Staretz-Chacham O, Choi JH, Wakabayashi K, Lopez G, Sidransky E: **Psychiatric and behavioral manifestations of lysosomal storage disorders**. *Am J Med Genet B Neuropsychiatr Genet* 2010, **153B**(7):1253-1265.

601. Goodspeed K, Horton D, Lowden A, Sguigna PV, Booth T, Wang ZJ, Edgar VB: **A cross-sectional natural history study of aspartylglucosaminuria**. *JIMD Rep* 2022, **63**(5):425-433.

602. Jain RS, Prakash S, Raghavendra BS, Nagpal K, Handa R: **Cyclic mood disorder heralding adult-onset autosomal dominant leucodystrophy: a clinical masquerader**. *Asian J Psychiatr* 2014, **9**:22-25.

603. Pedroso JL, Munford V, Bastos AU, Castro LP, Marussi VHR, Silva GS, Arita JH, Menck CFM, Barsottini OG: **LMNB1 mutation causes cerebellar involvement and a genome instability defect**. *J Neurol Sci* 2017, **379**:249-252.

604. Wang Q, Zhang J, Jiang N, Xie J, Yang J, Zhao X: **De novo nonsense variant in ASXL3 in a Chinese girl causing Bainbridge-Ropers syndrome: A case report and review of literature**. *Mol Genet Genomic Med* 2022, **10**(5):e1924.

605. Bortolato M, Floris G, Shih JC: **From aggression to autism: new perspectives on the behavioral sequelae of monoamine oxidase deficiency**. *J Neural Transm (Vienna)* 2018, **125**(11):1589-1599.

606. Palmer EE, Leffler M, Rogers C, Shaw M, Carroll R, Earl J, Cheung NW, Champion B, Hu H, Haas SA *et al*: **New insights into Brunner syndrome and potential for targeted therapy**. *Clin Genet* 2016, **89**(1):120-127.

607. Brunner HG, Nelen M, Breakefield XO, Ropers HH, van Oost BA: **Abnormal behavior associated with a point mutation in the structural gene for monoamine oxidase A**. *Science* 1993, **262**(5133):578-580.

608. Brunner HG, Nelen MR, van Zandvoort P, Abeling NG, van Gennip AH, Wolters EC, Kuiper MA, Ropers HH, van Oost BA: **X-linked borderline mental retardation with prominent behavioral disturbance: phenotype, genetic localization, and evidence for disturbed monoamine metabolism**. *Am J Hum Genet* 1993, **52**(6):1032-1039.

609. Valenti R, Pescini F, Antonini S, Castellini G, Poggesi A, Bianchi S, Inzitari D, Pallanti S, Pantoni L: **Major depression and bipolar disorders in CADASIL: a study using the DSM-IV semi-structured interview**. *Acta Neurol Scand* 2011, **124**(6):390-395.

610. Bianchi S, Zicari E, Carluccio A, Di Donato I, Pescini F, Nannucci S, Valenti R, Ragno M, Inzitari D, Pantoni L *et al*: **CADASIL in central Italy: a retrospective clinical and genetic study in 229 patients**. *J Neurol* 2015, **262**(1):134-141.

611. Ayrignac X, Carra-Dalliere C, Menjot de Champfleur N, Denier C, Aubourg P, Bellesme C, Castelnovo G, Pelletier J, Audoin B, Kaphan E *et al*: **Adult-onset genetic leukoencephalopathies: a MRI pattern-based approach in a comprehensive study of 154 patients**. *Brain* 2015, **138**(Pt 2):284-292.

612. Lee YC, Chung CP, Chao NC, Fuh JL, Chang FC, Soong BW, Liao YC: **Characterization of Heterozygous HTRA1 Mutations in Taiwanese Patients With Cerebral Small Vessel Disease**. *Stroke* 2018, **49**(7):1593-1601.

613. Nozaki H, Kato T, Nihonmatsu M, Saito Y, Mizuta I, Noda T, Koike R, Miyazaki K, Kaito M, Ito S *et al*: **Distinct molecular mechanisms of HTRA1 mutants in manifesting heterozygotes with CARASIL**. *Neurology* 2016, **86**(21):1964-1974.

614. Stockler-Ipsiroglu S, van Karnebeek CD: **Cerebral creatine deficiencies: a group of treatable intellectual developmental disorders**. *Semin Neurol* 2014, **34**(3):350-356.

615. Stockler-Ipsiroglu S, Apatean D, Battini R, DeBrosse S, Dessoffy K, Edvardson S, Eichler F, Johnston K, Koeller DM, Nouioua S *et al*: **Arginine:glycine amidinotransferase (AGAT) deficiency: Clinical features and long term outcomes in 16 patients diagnosed worldwide**. *Mol Genet Metab* 2015, **116**(4):252-259.

616. Degos B, Nadjar Y, Amador Mdel M, Lamari F, Sedel F, Roze E, Couvert P, Mochel F: **Natural history of cerebrotendinous xanthomatosis: a paediatric disease diagnosed in adulthood**. *Orphanet J Rare Dis* 2016, **11**:41.

617. Wachtel L, Hartshorne T, Dailor A: **Psychiatric diagnoses and psychotropic medications in CHARGE syndrome: A pediatric survey**. *Journal of Developmental and Physical Disabilities* 2007, **19**(1056-263X):471-483.

618. Thomas AT, Waite J, Williams CA, Kirk J, Oliver C, Richards C: **Phenotypic characteristics and variability in CHARGE syndrome: a PRISMA compliant systematic review and meta-analysis**. *J Neurodev Disord* 2022, **14**(1):49.

619. Blake KD, Salem-Hartshorne N, Daoud MA, Gradstein J: **Adolescent and adult issues in CHARGE syndrome**. *Clin Pediatr (Phila)* 2005, **44**(2):151-159.

620. Graham JM, Jr., Rosner B, Dykens E, Visootsak J: **Behavioral features of CHARGE syndrome (Hall-Hittner syndrome) comparison with Down syndrome, Prader-Willi syndrome, and Williams syndrome**. *Am J Med Genet A* 2005, **133A**(3):240-247.

621. Bergman JE, Janssen N, Hoefsloot LH, Jongmans MC, Hofstra RM, van Ravenswaaij-Arts CM: **CHD7 mutations and CHARGE syndrome: the clinical implications of an expanding phenotype**. *J Med Genet* 2011, **48**(5):334-342.

622. Hartshorne TS, Stratton KK, Brown D, Madhavan-Brown S, Schmittel MC: **Behavior in CHARGE syndrome**. *Am J Med Genet C Semin Med Genet* 2017, **175**(4):431-438.

623. Hardie RJ, Pullon HW, Harding AE, Owen JS, Pires M, Daniels GL, Imai Y, Misra VP, King RH, Jacobs JM *et al*: **Neuroacanthocytosis. A clinical, haematological and pathological study of 19 cases**. *Brain* 1991, **114 ( Pt 1A)**:13-49.

624. Aasly J, Skandsen T, Ro M: **Neuroacanthocytosis--the variability of presenting symptoms in two siblings**. *Acta Neurol Scand* 1999, **100**(5):322-325.

625. Walterfang M, Yucel M, Walker R, Evans A, Bader B, Ng A, Danek A, Mocellin R, Velakoulis D: **Adolescent obsessive compulsive disorder heralding chorea-acanthocytosis**. *Mov Disord* 2008, **23**(3):422-425.

626. Walterfang M, Looi JC, Styner M, Walker RH, Danek A, Niethammer M, Evans A, Kotschet K, Rodrigues GR, Hughes A *et al*: **Shape alterations in the striatum in chorea-acanthocytosis**. *Psychiatry Res* 2011, **192**(1):29-36.

627. Ikeda S, Yazaki M, Takei Y, Ikegami T, Hashikura Y, Kawasaki S, Iwai M, Kobayashi K, Saheki T: **Type II (adult onset) citrullinaemia: clinical pictures and the therapeutic effect of liver transplantation**. *J Neurol Neurosurg Psychiatry* 2001, **71**(5):663-670.

628. Committee UNS: **Screening for Citrullinaemia and Argininosuccinate lyase deficiency**. In*.* Bazian Ltd; 2014.

629. Gregory A, Hayflick S: **Neurodegeneration with Brain Iron Accumulation Disorders Overview**. In: *GeneReviews((R)).* Edited by Adam MP, Feldman J, Mirzaa GM, Pagon RA, Wallace SE, Bean LJH, Gripp KW, Amemiya A. Seattle (WA); 1993.

630. Evers C, Seitz A, Assmann B, Opladen T, Karch S, Hinderhofer K, Granzow M, Paramasivam N, Eils R, Diessl N *et al*: **Diagnosis of CoPAN by whole exome sequencing: Waking up a sleeping tiger's eye**. *Am J Med Genet A* 2017, **173**(7):1878-1886.

631. Cavestro C, Diodato D, Tiranti V, Di Meo I: **Inherited Disorders of Coenzyme A Biosynthesis: Models, Mechanisms, and Treatments**. *Int J Mol Sci* 2023, **24**(6).

632. Hunter AG: **Coffin-Lowry syndrome: a 20-year follow-up and review of long-term outcomes**. *Am J Med Genet* 2002, **111**(4):345-355.

633. Sivagamasundari U, Fernando H, Jardine P, Rao JM, Lunt P, Jayewardene SL: **The association between Coffin-Lowry syndrome and psychosis: a family study**. *J Intellect Disabil Res* 1994, **38 ( Pt 5)**:469-473.

634. Gursoy S, Hazan F, Cetinoglu E: **Novel RPS6KA3 mutations cause Coffin-Lowry syndrome in two patients and concurrent compulsive eyebrow-pulling behavior in one of them**. *Psychiatr Genet* 2022, **32**(5):194-198.

635. Hanauer A, Young ID: **Coffin-Lowry syndrome: clinical and molecular features**. *J Med Genet* 2002, **39**(10):705-713.

636. Abbott MH, Folstein SE, Abbey H, Pyeritz RE: **Psychiatric manifestations of homocystinuria due to cystathionine beta-synthase deficiency: prevalence, natural history, and relationship to neurologic impairment and vitamin B6-responsiveness**. *Am J Med Genet* 1987, **26**(4):959-969.

637. Almuqbil MA, Waisbren SE, Levy HL, Picker JD: **Revising the Psychiatric Phenotype of Homocystinuria**. *Genet Med* 2019, **21**(8):1827-1831.

638. Cederlof M, Karlsson R, Larsson H, Almqvist C, Magnusson PK, Nordlind K, Landen M, Lichtenstein P: **Intellectual disability and cognitive ability in Darier disease: Swedish nation-wide study**. *Br J Dermatol* 2015, **173**(1):155-158.

639. Cederlof M, Bergen SE, Langstrom N, Larsson H, Boman M, Craddock N, Ostberg P, Lundstrom S, Sjolander A, Nordlind K *et al*: **The association between Darier disease, bipolar disorder, and schizophrenia revisited: a population-based family study**. *Bipolar Disord* 2015, **17**(3):340-344.

640. Adachi N, Arima K, Asada T, Kato M, Minami N, Goto Y, Onuma T, Ikeuchi T, Tsuji S, Hayashi M *et al*: **Dentatorubral-pallidoluysian atrophy (DRPLA) presenting with psychosis**. *J Neuropsychiatry Clin Neurosci* 2001, **13**(2):258-260.

641. Alves Junior AC, Daker MV, Machado AMC, Luna AS, Valladares Neto DC, Valadares ER: **Neuropsychiatric and sleep study in autosomal dominant dopa-responsive dystonia**. *Mol Genet Metab Rep* 2022, **31**:100870.

642. Earl RK, Turner TN, Mefford HC, Hudac CM, Gerdts J, Eichler EE, Bernier RA: **Clinical phenotype of ASD-associated DYRK1A haploinsufficiency**. *Mol Autism* 2017, **8**:54.

643. van Bon BW, Coe BP, Bernier R, Green C, Gerdts J, Witherspoon K, Kleefstra T, Willemsen MH, Kumar R, Bosco P *et al*: **Disruptive de novo mutations of DYRK1A lead to a syndromic form of autism and ID**. *Mol Psychiatry* 2016, **21**(1):126-132.

644. Berglund B, Pettersson C, Pigg M, Kristiansson P: **Self-reported quality of life, anxiety and depression in individuals with Ehlers-Danlos syndrome (EDS): a questionnaire study**. *BMC Musculoskelet Disord* 2015, **16**:89.

645. Ishiguro H, Yagasaki H, Horiuchi Y: **Ehlers-Danlos Syndrome in the Field of Psychiatry: A Review**. *Front Psychiatry* 2021, **12**:803898.

646. Kennedy M, Loomba K, Ghani H, Riley B: **The psychological burden associated with Ehlers-Danlos syndromes: a systematic review**. *J Osteopath Med* 2022, **122**(8):381-392.

647. Bolsover FE, Murphy E, Cipolotti L, Werring DJ, Lachmann RH: **Cognitive dysfunction and depression in Fabry disease: a systematic review**. *J Inherit Metab Dis* 2014, **37**(2):177-187.

648. Lelieveld IM, Bottcher A, Hennermann JB, Beck M, Fellgiebel A: **Eight-Year Follow-Up of Neuropsychiatric Symptoms and Brain Structural Changes in Fabry Disease**. *PLoS One* 2015, **10**(9):e0137603.

649. Lauterbach EC, Cummings JL, Duffy J, Coffey CE, Kaufer D, Lovell M, Malloy P, Reeve A, Royall DR, Rummans TA *et al*: **Neuropsychiatric correlates and treatment of lenticulostriatal diseases: a review of the literature and overview of research opportunities in Huntington's, Wilson's, and Fahr's diseases. A report of the ANPA Committee on Research. American Neuropsychiatric Association**. *J Neuropsychiatry Clin Neurosci* 1998, **10**(3):249-266.

650. Weisman DC, Yaari R, Hansen LA, Thal LJ: **Density of the brain, decline of the mind: an atypical case of Fahr disease**. *Arch Neurol* 2007, **64**(5):756-757.

651. Amisha F, Munakomi S: **Fahr Syndrome**. In: *StatPearls.* Treasure Island (FL); 2023.

652. Feely MP, O'Hare J, Veale D, Callaghan N: **Episodes of acute confusion or psychosis in familial hemiplegic migraine**. *Acta Neurol Scand* 1982, **65**(4):369-375.

653. LaBianca S, Jensen R, van den Maagdenberg AM, Baandrup L, Bendtsen L: **Familial Hemiplegic Migraine and Recurrent Episodes of Psychosis: A Case Report**. *Headache* 2015, **55**(7):1004-1007.

654. Barros J, Mendes A, Matos I, Pereira-Monteiro J: **Psychotic aura symptoms in familial hemiplegic migraine type 2 (ATP1A2)**. *J Headache Pain* 2012, **13**(7):581-585.

655. Spranger M, Spranger S, Schwab S, Benninger C, Dichgans M: **Familial hemiplegic migraine with cerebellar ataxia and paroxysmal psychosis**. *Eur Neurol* 1999, **41**(3):150-152.

656. Santoro L, Manganelli F, Fortunato MR, Soldovieri MV, Ambrosino P, Iodice R, Pisciotta C, Tessa A, Santorelli F, Taglialatela M: **A new Italian FHM2 family: clinical aspects and functional analysis of the disease-associated mutation**. *Cephalalgia* 2011, **31**(7):808-819.

657. Wong LC, Singh S, Wang HP, Hsu CJ, Hu SC, Lee WT: **FOXG1-Related Syndrome: From Clinical to Molecular Genetics and Pathogenic Mechanisms**. *Int J Mol Sci* 2019, **20**(17).

658. Wong LC, Huang CH, Chou WY, Hsu CJ, Tsai WC, Lee WT: **The clinical and sleep manifestations in children with FOXG1 syndrome**. *Autism Res* 2023, **16**(5):953-966.

659. Wadell PM, Hagerman RJ, Hessl DR: **Fragile X Syndrome: Psychiatric Manifestations, Assessment and Emerging Therapies**. *Curr Psychiatry Rev* 2013, **9**(1):53-58.

660. Davidson M, Sebastian SA, Benitez Y, Desai S, Quinonez J, Ruxmohan S, Stein JD, Cueva W: **Behavioral Problems in Fragile X Syndrome: A Review of Clinical Management**. *Cureus* 2022, **14**(2):e21840.

661. Knight SJ, Ritchie RJ, Chakrabarti L, Cross G, Taylor GR, Mueller RF, Hurst J, Paterson J, Yates JR, Dow DJ *et al*: **A study of FRAXE in mentally retarded individuals referred for fragile X syndrome (FRAXA) testing in the United Kingdom**. *Am J Hum Genet* 1996, **58**(5):906-913.

662. Fichera M, Castaldo A, Mongelli A, Marchini G, Gellera C, Nanetti L, Mariotti C: **Comorbidities in Friedreich ataxia: incidence and manifestations from early to advanced disease stages**. *Neurol Sci* 2022, **43**(12):6831-6838.

663. Nieto A, Hernandez-Torres A, Perez-Flores J, Monton F: **Depressive symptoms in Friedreich ataxia**. *Int J Clin Health Psychol* 2018, **18**(1):18-26.

664. Ducharme S, Dols A, Laforce R, Devenney E, Kumfor F, van den Stock J, Dallaire-Theroux C, Seelaar H, Gossink F, Vijverberg E *et al*: **Recommendations to distinguish behavioural variant frontotemporal dementia from psychiatric disorders**. *Brain* 2020, **143**(6):1632-1650.

665. Woolley JD, Khan BK, Murthy NK, Miller BL, Rankin KP: **The diagnostic challenge of psychiatric symptoms in neurodegenerative disease: rates of and risk factors for prior psychiatric diagnosis in patients with early neurodegenerative disease**. *J Clin Psychiatry* 2011, **72**(2):126-133.

666. Tantawy AAG, Adly AAM, Salah El-Din NY, Abdeen M: **Psychiatric manifestations in Egyptian Gaucher patients on enzyme replacement therapy**. *J Psychosom Res* 2019, **122**:75-81.

667. Packman W, Wilson Crosbie T, Riesner A, Fairley C, Packman S: **Psychological complications of patients with Gaucher disease**. *J Inherit Metab Dis* 2006, **29**(1):99-105.

668. Packman W, Crosbie TW, Behnken M, Eudy K, Packman S: **Living with Gaucher disease: Emotional health, psychosocial needs and concerns of individuals with Gaucher disease**. *Am J Med Genet A* 2010, **152A**(8):2002-2010.

669. Abdelwahab M, Potegal M, Shapiro EG, Nestrasil I: **Previously unrecognized behavioral phenotype in Gaucher disease type 3**. *Neurol Genet* 2017, **3**(3):e158.

670. Bizec CL, Nicole S, Panagiotakaki E, Seta N, Vuillaumier-Barrot S: **No Mutation in the SLC2A3 Gene in Cohorts of GLUT1 Deficiency Syndrome-Like Patients Negative for SLC2A1 and in Patients with AHC Negative for ATP1A3**. *JIMD Rep* 2014, **12**:115-120.

671. Hao J, Kelly DI, Su J, Pascual JM: **Clinical Aspects of Glucose Transporter Type 1 Deficiency: Information From a Global Registry**. *JAMA Neurol* 2017, **74**(6):727-732.

672. Iskusnykh IY, Zakharova AA, Pathak D: **Glutathione in Brain Disorders and Aging**. *Molecules* 2022, **27**(1).

673. Singh V, Kishore N, Kumar D, Sengupta S: **Glucose-6 phosphate dehydrogenase deficiency and psychotic illness**. *Indian J Psychol Med* 2012, **34**(3):270-272.

674. Kravetz Z, Schmidt-Kastner R: **New aspects for the brain in Hartnup disease based on mining of high-resolution cellular mRNA expression data for SLC6A19**. *IBRO Neurosci Rep* 2023, **14**:393-397.

675. Hashmi MS, Gupta V: **Hartnup Disease**. In: *StatPearls.* Treasure Island (FL); 2024.

676. Wang B, Bissell DM: **Hereditary Coproporphyria**. In: *GeneReviews((R)).* Edited by Adam MP, Mirzaa GM, Pagon RA, Wallace SE, Bean LJH, Gripp KW, Amemiya A. Seattle (WA); 1993.

677. Siegesmund M, van Tuyll van Serooskerken AM, Poblete-Gutierrez P, Frank J: **The acute hepatic porphyrias: current status and future challenges**. *Best Pract Res Clin Gastroenterol* 2010, **24**(5):593-605.

678. Anderson KE, Bloomer JR, Bonkovsky HL, Kushner JP, Pierach CA, Pimstone NR, Desnick RJ: **Recommendations for the diagnosis and treatment of the acute porphyrias**. *Ann Intern Med* 2005, **142**(6):439-450.

679. Cao LX, Yin JH, Du G, Yang Q, Huang Y: **Identifying and verifying Huntington's disease subtypes: Clinical features, neuroimaging, and cytokine changes**. *Brain Behav* 2024, **14**(3):e3469.

680. McAllister B, Gusella JF, Landwehrmeyer GB, Lee JM, MacDonald ME, Orth M, Rosser AE, Williams NM, Holmans P, Jones L *et al*: **Timing and Impact of Psychiatric, Cognitive, and Motor Abnormalities in Huntington Disease**. *Neurology* 2021, **96**(19):e2395-e2406.

681. Beglinger LJ, Langbehn DR, Duff K, Stierman L, Black DW, Nehl C, Anderson K, Penziner E, Paulsen JS, Huntington Study Group I: **Probability of obsessive and compulsive symptoms in Huntington's disease**. *Biol Psychiatry* 2007, **61**(3):415-418.

682. Loberti L, Bruno LP, Granata S, Doddato G, Resciniti S, Fava F, Carullo M, Rahikkala E, Jouret G, Menke LA *et al*: **Natural history of KBG syndrome in a large European cohort**. *Hum Mol Genet* 2022, **31**(24):4131-4142.

683. Low K, Ashraf T, Canham N, Clayton-Smith J, Deshpande C, Donaldson A, Fisher R, Flinter F, Foulds N, Fryer A *et al*: **Clinical and genetic aspects of KBG syndrome**. *Am J Med Genet A* 2016, **170**(11):2835-2846.

684. Vermeulen K, de Boer A, Janzing JGE, Koolen DA, Ockeloen CW, Willemsen MH, Verhoef FM, van Deurzen PAM, van Dongen L, van Bokhoven H *et al*: **Adaptive and maladaptive functioning in Kleefstra syndrome compared to other rare genetic disorders with intellectual disabilities**. *Am J Med Genet A* 2017, **173**(7):1821-1830.

685. Verhoeven WMA, Kleefstra T, Egger JIM: **Behavioral phenotype in the 9q subtelomeric deletion syndrome: a report about two adult patients**. *Am J Med Genet B Neuropsychiatr Genet* 2010, **153B**(2):536-541.

686. Vermeulen K, Staal WG, Janzing JGE, Buitelaar JK, Hans, Bokhoven V, Egger JIM, Tjitske, Kleefstra: **From a Single Gene Defect Towards a Cross Species Neurocognitive Phenotype: The EHMT1 Disruption Example (Kleefstra Syndrome)**. In*: 2015*; 2015.

687. Kleefstra T, de Leeuw N: **Kleefstra Syndrome**. In: *GeneReviews((R)).* Edited by Adam MP, Mirzaa GM, Pagon RA, Wallace SE, Bean LJH, Gripp KW, Amemiya A. Seattle (WA); 1993.

688. Segar DJ, Chodakiewitz YG, Torabi R, Cosgrove GR: **Deep brain stimulation for the obsessive-compulsive and Tourette-like symptoms of Kleefstra syndrome**. *Neurosurg Focus* 2015, **38**(6):E12.

689. Debs R, Froissart R, Aubourg P, Papeix C, Douillard C, Degos B, Fontaine B, Audoin B, Lacour A, Said G *et al*: **Krabbe disease in adults: phenotypic and genotypic update from a series of 11 cases and a review**. *J Inherit Metab Dis* 2013, **36**(5):859-868.

690. Behrens MI, Bruggemann N, Chana P, Venegas P, Kagi M, Parrao T, Orellana P, Garrido C, Rojas CV, Hauke J *et al*: **Clinical spectrum of Kufor-Rakeb syndrome in the Chilean kindred with ATP13A2 mutations**. *Mov Disord* 2010, **25**(12):1929-1937.

691. Balint B, Damasio J, Magrinelli F, Guerreiro R, Bras J, Bhatia KP: **Psychiatric Manifestations of ATP13A2 Mutations**. *Mov Disord Clin Pract* 2020, **7**(7):838-841.

692. Trakadis YJ, Fulginiti V, Walterfang M: **Inborn errors of metabolism associated with psychosis: literature review and case-control study using exome data from 5090 adult individuals**. *J Inherit Metab Dis* 2018, **41**(4):613-621.

693. El Tahry R, de Tourtchaninoff M, Vrielynck P, Van Rijckevorsel K: **Lafora disease: psychiatric manifestations, cognitive decline, and visual hallucinations**. *Acta Neurol Belg* 2015, **115**(3):471-474.

694. Endres D, Pruss H, Dressing A, Schneider J, Feige B, Schweizer T, Venhoff N, Nickel K, Meixensberger S, Matysik M *et al*: **Psychiatric Manifestation of Anti-LGI1 Encephalitis**. *Brain Sci* 2020, **10**(6).

695. Wu H, Mei F, Liu L, Zhang L, Hao H, Zhang S: **Case Report/Case Series: Rare case of anti-LGI1 limbic encephalitis with rapidly progressive dementia, psychiatric symptoms, and frequently seizures: A case report**. *Medicine (Baltimore)* 2021, **100**(29):e26654.

696. Li LH, Ma CC, Zhang HF, Lian YJ: **Clinical and electrographic characteristics of seizures in LGI1-antibody encephalitis**. *Epilepsy Behav* 2018, **88**:277-282.

697. Munoz-Lopetegi A, Guasp M, Prades L, Martinez-Hernandez E, Rosa-Justicia M, Patricio V, Armangue T, Rami L, Borras R, Castro-Fornieles J *et al*: **Neurological, psychiatric, and sleep investigations after treatment of anti-leucine-rich glioma-inactivated protein 1 (LGI1) encephalitis in Spain: a prospective cohort study**. *Lancet Neurol* 2024, **23**(3):256-266.

698. Konno T, Yoshida K, Mizuno T, Kawarai T, Tada M, Nozaki H, Ikeda SI, Nishizawa M, Onodera O, Wszolek ZK *et al*: **Clinical and genetic characterization of adult-onset leukoencephalopathy with axonal spheroids and pigmented glia associated with CSF1R mutation**. *Eur J Neurol* 2017, **24**(1):37-45.

699. Lerma-Carrillo I, Molina JD, Cuevas-Duran T, Julve-Correcher C, Espejo-Saavedra JM, Andrade-Rosa C, Lopez-Munoz F: **Psychopathology in the Lujan-Fryns syndrome: report of two patients and review**. *Am J Med Genet A* 2006, **140**(24):2807-2811.

700. Purandare KN, Markar TN: **Psychiatric symptomatology of Lujan-Fryns syndrome: an X-linked syndrome displaying Marfanoid symptoms with autistic features, hyperactivity, shyness and schizophreniform symptoms**. *Psychiatr Genet* 2005, **15**(3):229-231.

701. Priolo M, Schanze D, Tatton-Brown K, Mulder PA, Tenorio J, Kooblall K, Acero IH, Alkuraya FS, Arias P, Bernardini L *et al*: **Further delineation of Malan syndrome**. *Hum Mutat* 2018, **39**(9):1226-1237.

702. Alfieri P, Montanaro FAM, Macchiaiolo M, Collotta M, Caciolo C, Galassi P, Panfili FM, Cortellessa F, Zollino M, Chinali M *et al*: **Behavioral profiling in children and adolescents with Malan syndrome**. *Frontiers in Child and Adolescent Psychiatry* 2023, **2**:1-10.

703. Braverman AC, Harris KM, Kovacs RJ, Maron BJ, American Heart Association E, Arrhythmias Committee of Council on Clinical Cardiology CoCDiYCoC, Stroke Nursing CoFG, Translational B, American College of C: **Eligibility and Disqualification Recommendations for Competitive Athletes With Cardiovascular Abnormalities: Task Force 7: Aortic Diseases, Including Marfan Syndrome: A Scientific Statement From the American Heart Association and American College of Cardiology**. *Circulation* 2015, **132**(22):e303-309.

704. Moura DS, Sultan S, Georgin-Lavialle S, Pillet N, Montestruc F, Gineste P, Barete S, Damaj G, Moussy A, Lortholary O *et al*: **Depression in patients with mastocytosis: prevalence, features and effects of masitinib therapy**. *PLoS One* 2011, **6**(10):e26375.

705. Spolak-Bobryk N, Romantowski J, Kujawska-Danecka H, Niedoszytko M: **Mastocytosis patients' cognitive dysfunctions correlate with the presence of spindle-shaped mast cells in bone marrow**. *Clin Transl Allergy* 2022, **12**(1):e12093.

706. Roulis E, Hyland C, Flower R, Gassner C, Jung HH, Frey BM: **Molecular Basis and Clinical Overview of McLeod Syndrome Compared With Other Neuroacanthocytosis Syndromes: A Review**. *JAMA Neurol* 2018, **75**(12):1554-1562.

707. Jung HH, Danek A, Frey BM: **McLeod syndrome: a neurohaematological disorder**. *Vox Sang* 2007, **93**(2):112-121.

708. Danek A, Rubio JP, Rampoldi L, Ho M, Dobson-Stone C, Tison F, Symmans WA, Oechsner M, Kalckreuth W, Watt JM *et al*: **McLeod neuroacanthocytosis: genotype and phenotype**. *Ann Neurol* 2001, **50**(6):755-764.

709. Magner M, Honzik T, Tesarova M, Dvorakova V, Hansikova H, Raboch J, Zeman J: **[Psychiatric disturbances in five patients with MELAS syndrome]**. *Psychiatr Pol* 2014, **48**(5):1035-1045.

710. Anglin RE, Garside SL, Tarnopolsky MA, Mazurek MF, Rosebush PI: **The psychiatric manifestations of mitochondrial disorders: a case and review of the literature**. *J Clin Psychiatry* 2012, **73**(4):506-512.

711. Chang SC, Eichinger CS, Field P: **The natural history and burden of illness of metachromatic leukodystrophy: a systematic literature review**. *Eur J Med Res* 2024, **29**(1):181.

712. Forny P, Horster F, Ballhausen D, Chakrapani A, Chapman KA, Dionisi-Vici C, Dixon M, Grunert SC, Grunewald S, Haliloglu G *et al*: **Guidelines for the diagnosis and management of methylmalonic acidaemia and propionic acidaemia: First revision**. *J Inherit Metab Dis* 2021, **44**(3):566-592.

713. Nizon M, Ottolenghi C, Valayannopoulos V, Arnoux JB, Barbier V, Habarou F, Desguerre I, Boddaert N, Bonnefont JP, Acquaviva C *et al*: **Long-term neurological outcome of a cohort of 80 patients with classical organic acidurias**. *Orphanet J Rare Dis* 2013, **8**:148.

714. Wijburg FA, Wegrzyn G, Burton BK, Tylki-Szymanska A: **Mucopolysaccharidosis type III (Sanfilippo syndrome) and misdiagnosis of idiopathic developmental delay, attention deficit/hyperactivity disorder or autism spectrum disorder**. *Acta Paediatr* 2013, **102**(5):462-470.

715. Cross EM, Hare DJ: **Behavioural phenotypes of the mucopolysaccharide disorders: a systematic literature review of cognitive, motor, social, linguistic and behavioural presentation in the MPS disorders**. *J Inherit Metab Dis* 2013, **36**(2):189-200.

716. Valstar MJ, Marchal JP, Grootenhuis M, Colland V, Wijburg FA: **Cognitive development in patients with Mucopolysaccharidosis type III (Sanfilippo syndrome)**. *Orphanet J Rare Dis* 2011, **6**:43.

717. do Valle DA, Santos M, Telles BA, Cordeiro ML: **Neurological, neurobehavioral, and radiological alterations in patients with mucopolysaccharidosis III (Sanfilippo's syndrome) in Brazil**. *Front Neurol* 2022, **13**:968297.

718. Kuiper GA, Meijer OLM, Langereis EJ, Wijburg FA: **Failure to shorten the diagnostic delay in two ultra-orphan diseases (mucopolysaccharidosis types I and III): potential causes and implications**. *Orphanet J Rare Dis* 2018, **13**(1):2.

719. Yang DD, Rio M, Michot C, Boddaert N, Yacoub W, Garcelon N, Thierry B, Bonnet D, Rondeau S, Herve D *et al*: **Natural history of Myhre syndrome**. *Orphanet J Rare Dis* 2022, **17**(1):304.

720. Peall KJ, Smith DJ, Kurian MA, Wardle M, Waite AJ, Hedderly T, Lin JP, Smith M, Whone A, Pall H *et al*: **SGCE mutations cause psychiatric disorders: clinical and genetic characterization**. *Brain* 2013, **136**(Pt 1):294-303.

721. Nardocci N, Zorzi G, Barzaghi C, Zibordi F, Ciano C, Ghezzi D, Garavaglia B: **Myoclonus-dystonia syndrome: clinical presentation, disease course, and genetic features in 11 families**. *Mov Disord* 2008, **23**(1):28-34.

722. Hess CW, Raymond D, Aguiar Pde C, Frucht S, Shriberg J, Heiman GA, Kurlan R, Klein C, Bressman SB, Ozelius LJ *et al*: **Myoclonus-dystonia, obsessive-compulsive disorder, and alcohol dependence in SGCE mutation carriers**. *Neurology* 2007, **68**(7):522-524.

723. Peall KJ, Waite AJ, Blake DJ, Owen MJ, Morris HR: **Psychiatric disorders, myoclonus dystonia, and the epsilon-sarcoglycan gene: a systematic review**. *Mov Disord* 2011, **26**(10):1939-1942.

724. Klunemann HH, Ridha BH, Magy L, Wherrett JR, Hemelsoet DM, Keen RW, De Bleecker JL, Rossor MN, Marienhagen J, Klein HE *et al*: **The genetic causes of basal ganglia calcification, dementia, and bone cysts: DAP12 and TREM2**. *Neurology* 2005, **64**(9):1502-1507.

725. Paloneva J, Autti T, Raininko R, Partanen J, Salonen O, Puranen M, Hakola P, Haltia M: **CNS manifestations of Nasu-Hakola disease: a frontal dementia with bone cysts**. *Neurology* 2001, **56**(11):1552-1558.

726. Keogh M, Singh B, Chinnery PF: **Early neuropsychiatry features in neuroferritinopathy**. *Movement Disorders* 2013, **28**.

727. Lehwald LM, Pappa R, Steward S, de Los Reyes E: **Neuronal Ceroid Lipofuscinosis and Associated Sleep Abnormalities**. *Pediatr Neurol* 2016, **59**:30-35.

728. Nita DA, Mole SE, Minassian BA: **Neuronal ceroid lipofuscinoses**. *Epileptic Disord* 2016, **18**(S2):73-88.

729. Valadares ER, Pizarro MX, Oliveira LR, Amorim RH, Pinheiro TM, Grieben U, Santos HH, Queiroz RR, Lopes Gde C, Godard AL: **Juvenile neuronal ceroid-lipofuscinosis: clinical and molecular investigation in a large family in Brazil**. *Arq Neuropsiquiatr* 2011, **69**(1):13-18.

730. Dolisca SB, Mehta M, Pearce DA, Mink JW, Maria BL: **Batten disease: clinical aspects, molecular mechanisms, translational science, and future directions**. *J Child Neurol* 2013, **28**(9):1074-1100.

731. Nadjar Y, Hutter-Moncada AL, Latour P, Ayrignac X, Kaphan E, Tranchant C, Cintas P, Degardin A, Goizet C, Laurencin C *et al*: **Adult Niemann-Pick disease type C in France: clinical phenotypes and long-term miglustat treatment effect**. *Orphanet J Rare Dis* 2018, **13**(1):175.

732. Dinopoulos A, Kure S, Chuck G, Sato K, Gilbert DL, Matsubara Y, Degrauw T: **Glycine decarboxylase mutations: a distinctive phenotype of nonketotic hyperglycinemia in adults**. *Neurology* 2005, **64**(7):1255-1257.

733. Farris J, Calhoun B, Alam MS, Lee S, Haldar K: **Large scale analyses of genotype-phenotype relationships of glycine decarboxylase mutations and neurological disease severity**. *PLoS Comput Biol* 2020, **16**(5):e1007871.

734. Brassier A, Gobin S, Arnoux JB, Valayannopoulos V, Habarou F, Kossorotoff M, Servais A, Barbier V, Dubois S, Touati G *et al*: **Long-term outcomes in Ornithine Transcarbamylase deficiency: a series of 90 patients**. *Orphanet J Rare Dis* 2015, **10**:58.

735. Finkelstein JE, Hauser ER, Leonard CO, Brusilow SW: **Late-onset ornithine transcarbamylase deficiency in male patients**. *J Pediatr* 1990, **117**(6):897-902.

736. Lu D, Han F, Qiu W, Zhang H, Ye J, Liang L, Wang Y, Ji W, Zhan X, Gu X *et al*: **Clinical and molecular characteristics of 69 Chinese patients with ornithine transcarbamylase deficiency**. *Orphanet J Rare Dis* 2020, **15**(1):340.

737. Kadono S, Miyawaki D, Goto A, Hirai K, Sakamoto S, Hama H, Nishiura S, Hamazaki T, Inoue K: **Delayed recognition of autism spectrum disorder and attention-deficit/hyperactivity disorder in a girl with ornithine transcarbamylase deficiency: A case report**. *Medicine (Baltimore)* 2023, **102**(8):e33055.

738. Hartig MB, Hortnagel K, Garavaglia B, Zorzi G, Kmiec T, Klopstock T, Rostasy K, Svetel M, Kostic VS, Schuelke M *et al*: **Genotypic and phenotypic spectrum of PANK2 mutations in patients with neurodegeneration with brain iron accumulation**. *Ann Neurol* 2006, **59**(2):248-256.

739. Chang X, Zhang J, Jiang Y, Wang J, Wu Y: **Natural history and genotype-phenotype correlation of pantothenate kinase-associated neurodegeneration**. *CNS Neurosci Ther* 2020, **26**(7):754-761.

740. Hayflick SJ, Westaway SK, Levinson B, Zhou B, Johnson MA, Ching KH, Gitschier J: **Genetic, clinical, and radiographic delineation of Hallervorden-Spatz syndrome**. *N Engl J Med* 2003, **348**(1):33-40.

741. Wider C, Wszolek ZK: **Rapidly progressive familial parkinsonism with central hypoventilation, depression and weight loss (Perry syndrome)--a literature review**. *Parkinsonism Relat Disord* 2008, **14**(1):1-7.

742. Mishima T, Fujioka S, Tomiyama H, Yabe I, Kurisaki R, Fujii N, Neshige R, Ross OA, Farrer MJ, Dickson DW *et al*: **Establishing diagnostic criteria for Perry syndrome**. *J Neurol Neurosurg Psychiatry* 2018, **89**(5):482-487.

743. Kohlenberg TM, Trelles MP, McLarney B, Betancur C, Thurm A, Kolevzon A: **Psychiatric illness and regression in individuals with Phelan-McDermid syndrome**. *J Neurodev Disord* 2020, **12**(1):7.

744. von und zu Fraunberg M, Timonen K, Mustajoki P, Kauppinen R: **Clinical and biochemical characteristics and genotype-phenotype correlation in Finnish variegate porphyria patients**. *Eur J Hum Genet* 2002, **10**(10):649-657.

745. Hift RJ, Peters TJ, Meissner PN: **A review of the clinical presentation, natural history and inheritance of variegate porphyria: its implausibility as the source of the 'Royal Malady'**. *J Clin Pathol* 2012, **65**(3):200-205.

746. Stolzel U, Doss MO, Schuppan D: **Clinical Guide and Update on Porphyrias**. *Gastroenterology* 2019, **157**(2):365-381 e364.

747. Klauck SM, Lindsay S, Beyer KS, Splitt M, Burn J, Poustka A: **A mutation hot spot for nonspecific X-linked mental retardation in the MECP2 gene causes the PPM-X syndrome**. *Am J Hum Genet* 2002, **70**(4):1034-1037.

748. Maiti A, Chatterjee S: **Neuropsychiatric manifestations and their outcomes in chronic hypocalcaemia**. *J Indian Med Assoc* 2013, **111**(3):174-177.

749. Perez KM, Lee EB, Kahanda S, Duis J, Reyes M, Juppner H, Shoemaker AH: **Cognitive and behavioral phenotype of children with pseudohypoparathyroidism type 1A**. *Am J Med Genet A* 2018, **176**(2):283-289.

750. Boban S, Wong K, Epstein A, Anderson B, Murphy N, Downs J, Leonard H: **Determinants of sleep disturbances in Rett syndrome: Novel findings in relation to genotype**. *Am J Med Genet A* 2016, **170**(9):2292-2300.

751. Neul JL, Kaufmann WE, Glaze DG, Christodoulou J, Clarke AJ, Bahi-Buisson N, Leonard H, Bailey ME, Schanen NC, Zappella M *et al*: **Rett syndrome: revised diagnostic criteria and nomenclature**. *Ann Neurol* 2010, **68**(6):944-950.

752. Buchanan CB, Stallworth JL, Scott AE, Glaze DG, Lane JB, Skinner SA, Tierney AE, Percy AK, Neul JL, Kaufmann WE: **Behavioral profiles in Rett syndrome: Data from the natural history study**. *Brain Dev* 2019, **41**(2):123-134.

753. Stephen CD, Balkwill D, James P, Haxton E, Sassower K, Schmahmann JD, Eichler F, Lewis R: **Quantitative oculomotor and nonmotor assessments in late-onset GM2 gangliosidosis**. *Neurology* 2020, **94**(7):e705-e717.

754. Masingue M, Dufour L, Lenglet T, Saleille L, Goizet C, Ayrignac X, Ory-Magne F, Barth M, Lamari F, Mandia D *et al*: **Natural History of Adult Patients with GM2 Gangliosidosis**. *Ann Neurol* 2020, **87**(4):609-617.

755. Van Hove JL, Steyaert J, Matthijs G, Legius E, Theys P, Wevers R, Romstad A, Moller LB, Hedrich K, Goriounov D *et al*: **Expanded motor and psychiatric phenotype in autosomal dominant Segawa syndrome due to GTP cyclohydrolase deficiency**. *J Neurol Neurosurg Psychiatry* 2006, **77**(1):18-23.

756. Timmers ER, Kuiper A, Smit M, Bartels AL, Kamphuis DJ, Wolf NI, Poll-The BT, Wassenberg T, Peeters EAJ, de Koning TJ *et al*: **Non-motor symptoms and quality of life in dopa-responsive dystonia patients**. *Parkinsonism Relat Disord* 2017, **45**:57-62.

757. Poisson A, Nicolas A, Cochat P, Sanlaville D, Rigard C, de Leersnyder H, Franco P, Des Portes V, Edery P, Demily C: **Behavioral disturbance and treatment strategies in Smith-Magenis syndrome**. *Orphanet J Rare Dis* 2015, **10**:111.

758. Garayzabal E, Hidalgo I, Miranda de Souza ALD, da Silva NC, Giacheti CM, Pinato L: **Sleep disturbances and behavior in Smith-Magenis syndrome**. *Res Dev Disabil* 2022, **128**:104286.

759. Sheth K, Moss J, Hyland S, Stinton C, Cole T, Oliver C: **The behavioral characteristics of Sotos syndrome**. *Am J Med Genet A* 2015, **167A**(12):2945-2956.

760. Miles JH, Hadden LL, Takahashi TN, Hillman RE: **Head circumference is an independent clinical finding associated with autism**. *Am J Med Genet* 2000, **95**(4):339-350.

761. Lo RY, Figueroa KP, Pulst SM, Perlman S, Wilmot G, Gomez C, Schmahmann J, Paulson H, Shakkottai VG, Ying S *et al*: **Depression and clinical progression in spinocerebellar ataxias**. *Parkinsonism Relat Disord* 2016, **22**:87-92.

762. Opal P, Ashizawa T: **Spinocerebellar Ataxia Type 1**. In: *GeneReviews((R)).* Edited by Adam MP, Feldman J, Mirzaa GM, Pagon RA, Wallace SE, Bean LJH, Gripp KW, Amemiya A. Seattle (WA); 1993.

763. Klinke I, Minnerop M, Schmitz-Hubsch T, Hendriks M, Klockgether T, Wullner U, Helmstaedter C: **Neuropsychological features of patients with spinocerebellar ataxia (SCA) types 1, 2, 3, and 6**. *Cerebellum* 2010, **9**(3):433-442.

764. Pulst SM: **Spinocerebellar Ataxia Type 2**. In: *GeneReviews((R)).* Edited by Adam MP, Feldman J, Mirzaa GM, Pagon RA, Wallace SE, Bean LJH, Gripp KW, Amemiya A. Seattle (WA); 1993.

765. Burk K, Globas C, Bosch S, Graber S, Abele M, Brice A, Dichgans J, Daum I, Klockgether T: **Cognitive deficits in spinocerebellar ataxia 2**. *Brain* 1999, **122 ( Pt 4)**:769-777.

766. Silva UC, Marques W, Jr., Lourenco CM, Hallak JE, Osorio FL: **Psychiatric disorders, spinocerebellar ataxia type 3 and CAG expansion**. *J Neurol* 2015, **262**(7):1777-1779.

767. Maas R, Killaars S, van de Warrenburg BPC, Schutter D: **The cerebellar cognitive affective syndrome scale reveals early neuropsychological deficits in SCA3 patients**. *J Neurol* 2021, **268**(9):3456-3466.

768. Braga-Neto P, Pedroso JL, Gadelha A, Laureano MR, de Souza Noto C, Garrido GJ, Barsottini OG: **Psychosis in Machado-Joseph Disease: Clinical Correlates, Pathophysiological Discussion, and Functional Brain Imaging. Expanding the Cerebellar Cognitive Affective Syndrome**. *Cerebellum* 2016, **15**(4):483-490.

769. Coutelier M, Blesneac I, Monteil A, Monin ML, Ando K, Mundwiller E, Brusco A, Le Ber I, Anheim M, Castrioto A *et al*: **A Recurrent Mutation in CACNA1G Alters Cav3.1 T-Type Calcium-Channel Conduction and Causes Autosomal-Dominant Cerebellar Ataxia**. *Am J Hum Genet* 2015, **97**(5):726-737.

770. Morino H, Matsuda Y, Muguruma K, Miyamoto R, Ohsawa R, Ohtake T, Otobe R, Watanabe M, Maruyama H, Hashimoto K *et al*: **A mutation in the low voltage-gated calcium channel CACNA1G alters the physiological properties of the channel, causing spinocerebellar ataxia**. *Mol Brain* 2015, **8**:89.

771. Pearl PL, DiBacco ML, Papadelis C, Opladen T, Hanson E, Roullet JB, Gibson KM: **Succinic Semialdehyde Dehydrogenase Deficiency: Review of the Natural History Study**. *J Child Neurol* 2021, **36**(13-14):1153-1161.

772. Rong M, Benke T, Zulfiqar Ali Q, Aledo-Serrano A, Bayat A, Rossi A, Devinsky O, Qaiser F, Ali AS, Fasano A *et al*: **Adult Phenotype of SYNGAP1-DEE**. *Neurol Genet* 2023, **9**(6):e200105.

773. Vlaskamp DRM, Shaw BJ, Burgess R, Mei D, Montomoli M, Xie H, Myers CT, Bennett MF, XiangWei W, Williams D *et al*: **SYNGAP1 encephalopathy: A distinctive generalized developmental and epileptic encephalopathy**. *Neurology* 2019, **92**(2):e96-e107.

774. Tatton-Brown K, Zachariou A, Loveday C, Renwick A, Mahamdallie S, Aksglaede L, Baralle D, Barge-Schaapveld D, Blyth M, Bouma M *et al*: **The Tatton-Brown-Rahman Syndrome: A clinical study of 55 individuals with de novo constitutive DNMT3A variants**. *Wellcome Open Res* 2018, **3**:46.

775. Toro C, Shirvan L, Tifft C: **HEXA Disorders**. In: *GeneReviews((R)).* Edited by Adam MP, Feldman J, Mirzaa GM, Pagon RA, Wallace SE, Bean LJH, Gripp KW, Amemiya A. Seattle (WA); 1993.

776. Shapiro BE, Hatters-Friedman S, Fernandes-Filho JA, Anthony K, Natowicz MR: **Late-onset Tay-Sachs disease: adverse effects of medications and implications for treatment**. *Neurology* 2006, **67**(5):875-877.

777. MacQueen GM, Rosebush PI, Mazurek MF: **Neuropsychiatric aspects of the adult variant of Tay-Sachs disease**. *J Neuropsychiatry Clin Neurosci* 1998, **10**(1):10-19.

778. Pulsifer MB, Winterkorn EB, Thiele EA: **Psychological profile of adults with tuberous sclerosis complex**. *Epilepsy Behav* 2007, **10**(3):402-406.

779. Lewis JC, Thomas HV, Murphy KC, Sampson JR: **Genotype and psychological phenotype in tuberous sclerosis**. *J Med Genet* 2004, **41**(3):203-207.

780. Northrup H, Koenig MK, Pearson DA, Au KS: **Tuberous Sclerosis Complex**. In: *GeneReviews((R)).* Edited by Adam MP, Feldman J, Mirzaa GM, Pagon RA, Wallace SE, Bean LJH, Gripp KW, Amemiya A. Seattle (WA); 1993.

781. Miura S, Hiruki S, Okada T, Takei SI, Senzaki K, Okada Y, Ochi M, Tanabe Y, Ochi H, Igase M *et al*: **Case report: Frontotemporal dementia and amyotrophic lateral sclerosis caused by a missense variant (p.Arg89Trp) in the valosin-containing protein gene**. *Front Genet* 2023, **14**:1155998.

782. Papadimas GK, Paraskevas GP, Zambelis T, Karagiaouris C, Bourbouli M, Bougea A, Walter MC, Schumacher NU, Krause S, Kapaki E: **The multifaceted clinical presentation of VCP-proteinopathy in a Greek family**. *Acta Myol* 2017, **36**(4):203-206.

783. Goldman JS, Adamson J, Karydas A, Miller BL, Hutton M: **New genes, new dilemmas: FTLD genetics and its implications for families**. *Am J Alzheimers Dis Other Demen* 2007, **22**(6):507-515.

784. Korb M, Peck A, Alfano LN, Berger KI, James MK, Ghoshal N, Healzer E, Henchcliffe C, Khan S, Mammen PPA *et al*: **Development of a standard of care for patients with valosin-containing protein associated multisystem proteinopathy**. *Orphanet J Rare Dis* 2022, **17**(1):23.

785. Mah-Som AY, Daw J, Huynh D, Wu M, Creekmore BC, Burns W, Skinner SA, Holla OL, Smeland MF, Planes M *et al*: **An autosomal-dominant childhood-onset disorder associated with pathogenic variants in VCP**. *Am J Hum Genet* 2023, **110**(11):1959-1975.

786. Hamilton EMC, van der Lei HDW, Vermeulen G, Gerver JAM, Lourenco CM, Naidu S, Mierzewska H, Gemke R, de Vet HCW, Uitdehaag BMJ *et al*: **Natural History of Vanishing White Matter**. *Ann Neurol* 2018, **84**(2):274-288.

787. Labauge P, Horzinski L, Ayrignac X, Blanc P, Vukusic S, Rodriguez D, Mauguiere F, Peter L, Goizet C, Bouhour F *et al*: **Natural history of adult-onset eIF2B-related disorders: a multi-centric survey of 16 cases**. *Brain* 2009, **132**(Pt 8):2161-2169.

788. Benzoni C, Moscatelli M, Farina L, Magri S, Ciano C, Scaioli V, Alvera S, Cammarata G, Bianchi-Marzoli S, Castellani M *et al*: **Adult-onset leukodystrophy with vanishing white matter: a case series of 19 patients**. *J Neurol* 2023, **270**(9):4219-4234.

789. Wang B, Rudnick S, Cengia B, Bonkovsky HL: **Acute Hepatic Porphyrias: Review and Recent Progress**. *Hepatol Commun* 2019, **3**(2):193-206.

790. Millward LM, Kelly P, King A, Peters TJ: **Anxiety and depression in the acute porphyrias**. *J Inherit Metab Dis* 2005, **28**(6):1099-1107.

791. Kaftory R, Edel Y, Snast I, Lapidoth M, Mamet R, Elis A, Hodak E, Levi A: **Greater disease burden of variegate porphyria than hereditary coproporphyria: An Israeli nationwide study of neurocutaneous porphyrias**. *Mol Genet Metab Rep* 2021, **26**:100707.

792. Assia Batzir N, White J, Sutton VR: **White-Sutton Syndrome**. In: *GeneReviews((R)).* Edited by Adam MP, Feldman J, Mirzaa GM, Pagon RA, Wallace SE, Bean LJH, Gripp KW, Amemiya A. Seattle (WA); 1993.

793. Akil M, Schwartz JA, Dutchak D, Yuzbasiyan-Gurkan V, Brewer GJ: **The psychiatric presentations of Wilson's disease**. *J Neuropsychiatry Clin Neurosci* 1991, **3**(4):377-382.

794. Litwin T, Dusek P, Szafranski T, Dziezyc K, Czlonkowska A, Rybakowski JK: **Psychiatric manifestations in Wilson's disease: possibilities and difficulties for treatment**. *Ther Adv Psychopharmacol* 2018, **8**(7):199-211.

795. Reiersen AM, Noel JS, Doty T, Sinkre RA, Narayanan A, Hershey T: **Psychiatric Diagnoses and Medications in Wolfram Syndrome**. *Scand J Child Adolesc Psychiatr Psychol* 2022, **10**(1):163-174.

796. Swift RG, Sadler DB, Swift M: **Psychiatric findings in Wolfram syndrome homozygotes**. *Lancet* 1990, **336**(8716):667-669.

797. Woodhouse NJ, Sakati NA: **A syndrome of hypogonadism, alopecia, diabetes mellitus, mental retardation, deafness, and ECG abnormalities**. *J Med Genet* 1983, **20**(3):216-219.

798. Bohlega S, Abusrair AH, Al-Ajlan FS, Alharbi N, Al-Semari A, Bohlega B, Abualsaud D, Alkuraya F: **Patterns of neurological manifestations in Woodhouse-Sakati Syndrome**. *Parkinsonism Relat Disord* 2019, **69**:99-103.

799. Ali R, Al-Dewik N, Mohammed S, Elfituri M, Agouba S, Musa S, Mahmoud L, Almulla M, El-Akouri K, Mohd H *et al*: **Expanding on the phenotypic spectrum of Woodhouse-Sakati syndrome due to founder pathogenic variant in DCAF17: Report of 58 additional patients from Qatar and literature review**. *Am J Med Genet A* 2022, **188**(1):116-129.

800. Vodička S, Zelko E: **Management of mitochondrial leukoencephalopathy-related ataxia and cognitive impairment in a primary health care setup: A case report**. *Medicine Case Reports and Study Protocols* 2021, **2**(6):3.

801. Lobbes H, Reynaud Q, Mainbourg S, Lega JC, Durieu I, Durupt S: **[Aceruloplasminemia, a rare condition not to be overlooked]**. *Rev Med Interne* 2020, **41**(11):769-775.

802. Wang B, Bonkovsky HL, Lim JK, Balwani M: **AGA Clinical Practice Update on Diagnosis and Management of Acute Hepatic Porphyrias: Expert Review**. *Gastroenterology* 2023, **164**(3):484-491.

803. Horgan P, Jones H: **Olanzapine use in acute porphyria**. *Int J Psychiatry Clin Pract* 2003, **7**(1):67-69.

804. Palau-Hernandez S, Rodriguez-Leyva I, Shiguetomi-Medina JM: **Late onset adrenoleukodystrophy: A review related clinical case report**. *eNeurologicalSci* 2019, **14**:62-67.

805. Engelen M, van Ballegoij WJC, Mallack EJ, Van Haren KP, Kohler W, Salsano E, van Trotsenburg ASP, Mochel F, Sevin C, Regelmann MO *et al*: **International Recommendations for the Diagnosis and Management of Patients With Adrenoleukodystrophy: A Consensus-Based Approach**. *Neurology* 2022, **99**(21):940-951.

806. Adang LA, Sherbini O, Ball L, Bloom M, Darbari A, Amartino H, DiVito D, Eichler F, Escolar M, Evans SH *et al*: **Revised consensus statement on the preventive and symptomatic care of patients with leukodystrophies**. *Mol Genet Metab* 2017, **122**(1-2):18-32.

807. Borgwardt L, Guffon N, Amraoui Y, Dali CI, De Meirleir L, Gil-Campos M, Heron B, Geraci S, Ardigo D, Cattaneo F *et al*: **Efficacy and safety of Velmanase alfa in the treatment of patients with alpha-mannosidosis: results from the core and extension phase analysis of a phase III multicentre, double-blind, randomised, placebo-controlled trial**. *J Inherit Metab Dis* 2018, **41**(6):1215-1223.

808. Lund AM, Borgwardt L, Cattaneo F, Ardigo D, Geraci S, Gil-Campos M, De Meirleir L, Laroche C, Dolhem P, Cole D *et al*: **Comprehensive long-term efficacy and safety of recombinant human alpha-mannosidase (velmanase alfa) treatment in patients with alpha-mannosidosis**. *J Inherit Metab Dis* 2018, **41**(6):1225-1233.

809. **LAMZEDE (velmanase alfa-tycv) FDA Label**. In*.* Edited by Administration FaD; 2023.

810. Castro RG, Perez AMG, Curto MCR, Alvarez JC, Ferreiros AC, Cuadros AV, Bueno DM, Fernandez AJC: **A New Case of Schindler Disease**. *Eur J Case Rep Intern Med* 2019, **6**(11):001269.

811. Scheithauer M, Bernstein A, Stremel JM: **Treatment of Self-Injury in Bainbridge-Ropers Syndrome: Replication and Extensions of Behavioral Assessments**. *Behav Anal Pract* 2023, **16**(2):611-616.

812. Taylor MH, Doody GA: **CADASIL: a guide to a comparatively unrecognised condition in psychiatry**. *Advances in Psychiatric Treatment* 2008, **14**(5):350-357.

813. Jinnah HA: **HPRT1 Disorders**. In: *GeneReviews((R)).* Edited by Adam MP, Mirzaa GM, Pagon RA, Wallace SE, Bean LJH, Gripp KW, Amemiya A. Seattle (WA); 1993.

814. Viau KS, Ernst SL, Pasquali M, Botto LD, Hedlund G, Longo N: **Evidence-based treatment of guanidinoacetate methyltransferase (GAMT) deficiency**. *Mol Genet Metab* 2013, **110**(3):255-262.

815. Stöckler-Ipsiroglu S, Braissant O, Schulze A: **Disorders of Creatine Metabolism**. In: *Physician's Guide to the Diagnosis, Treatment, and Follow-Up of Inherited Metabolic Diseases.* Edited by Blau N, Dionisi Vici C, Ferreira CR, Vianey-Saban C, van Karnebeek CDM. Cham: Springer International Publishing; 2022: 235-249.

816. Termsarasab P, Thammongkolchai T, Frucht SJ: **Medical treatment of dystonia**. *J Clin Mov Disord* 2016, **3**:19.

817. van Ravenswaaij-Arts C, Martin DM: **New insights and advances in CHARGE syndrome: Diagnosis, etiologies, treatments, and research discoveries**. *Am J Med Genet C Semin Med Genet* 2017, **175**(4):397-406.

818. **Self-Regulation of Emotion in CHARGE Syndrome** [Self-Regulation of Emotion in CHARGE Syndrome]

819. Walker RH: **Management of Neuroacanthocytosis Syndromes**. *Tremor Other Hyperkinet Mov (N Y)* 2015, **5**:346.

820. **The American Psychiatric Association Practice Guideline for the Treatment of Patients With Eating Disorders**.

821. Arora S, Srivastava MVP, Singh MB, Goyal V, Haberle J, Gupta N, Prabhakar A, Aggarwal B, Agarwal A, Vishnu VY: **Adult onset type II citrullinemia--a great masquerader**. *QJM* 2020, **113**(1):49-51.

822. Hayasaka K, Numakura C: **Adult-onset type II citrullinemia: Current insights and therapy**. *Appl Clin Genet* 2018, **11**:163-170.

823. Wilson JL, Gregory A, Kurian MA, Bushlin I, Mochel F, Emrick L, Adang L, Group BGCA, Hogarth P, Hayflick SJ: **Consensus clinical management guideline for beta-propeller protein-associated neurodegeneration**. *Dev Med Child Neurol* 2021, **63**(12):1402-1409.

824. Nakamura M, Yamagata T, Momoi MY, Yamazaki T: **Drop episodes in Coffin-Lowry syndrome: exaggerated startle responses treated with clonazepam**. *Pediatr Neurol* 1998, **19**(2):148-150.

825. Majtan T, Kozich V, Kruger WD: **Recent therapeutic approaches to cystathionine beta-synthase-deficient homocystinuria**. *Br J Pharmacol* 2023, **180**(3):264-278.

826. Haber RN, Dib NG: **Management of Darier disease: A review of the literature and update**. *Indian J Dermatol Venereol Leprol* 2021, **87**(1):14-21.

827. Ngo J, Haber R: **Exacerbation of Darier disease by lithium carbonate**. *J Cutan Med Surg* 2010, **14**(2):80-84.

828. Narita Z, Sumiyoshi T: **Successful treatment of psychosis in dentatorubral-pallidoluysian atrophy with quetiapine: A case report**. *Neuropsychopharmacol Rep* 2018, **38**(1):44-46.

829. van Bon BWM, Coe BP, de Vries BBA, Eichler EE: **DYRK1A Syndrome**. In: *GeneReviews((R)).* Edited by Adam MP, Mirzaa GM, Pagon RA, Wallace SE, Bean LJH, Gripp KW, Amemiya A. Seattle (WA); 1993.

830. Ortiz A, Germain DP, Desnick RJ, Politei J, Mauer M, Burlina A, Eng C, Hopkin RJ, Laney D, Linhart A *et al*: **Fabry disease revisited: Management and treatment recommendations for adult patients**. *Mol Genet Metab* 2018, **123**(4):416-427.

831. Di Stefano V, Rispoli MG, Pellegrino N, Graziosi A, Rotondo E, Napoli C, Pietrobon D, Brighina F, Parisi P: **Diagnostic and therapeutic aspects of hemiplegic migraine**. *J Neurol Neurosurg Psychiatry* 2020, **91**(7):764-771.

832. Mjaset C, Russell MB: **Intravenous nimodipine worsening prolonged attack of familial hemiplegic migraine**. *J Headache Pain* 2008, **9**(6):381-384.

833. Papandreou A, Schneider RB, Augustine EF, Ng J, Mankad K, Meyer E, McTague A, Ngoh A, Hemingway C, Robinson R *et al*: **Delineation of the movement disorders associated with FOXG1 mutations**. *Neurology* 2016, **86**(19):1794-1800.

834. Ranjan R, Jha S, Prajjwal P, Chaudhary A, Dudeja P, Vora N, Mateen MA, Yousuf MA, Chaudhary B: **Neurological, Psychiatric, and Multisystemic Involvement of Fragile X Syndrome Along With Its Pathophysiology, Methods of Screening, and Current Treatment Modalities**. *Cureus* 2023, **15**(2):e35505.

835. Sachdeva A, Jain P, Gunasekaran V, Mahay SB, Mukherjee S, Hagerman R, Shankar S, Kapoor S, Kedia SN, Indian Academy of Pediatrics Consensus in D *et al*: **Consensus Statement of the Indian Academy of Pediatrics on Diagnosis and Management of Fragile X Syndrome in India**. *Indian Pediatr* 2019, **56**(3):221-228.

836. Hagerman RJ, Polussa J: **Treatment of the psychiatric problems associated with fragile X syndrome**. *Curr Opin Psychiatry* 2015, **28**(2):107-112.

837. Correia F, Cafe C, Almeida J, Mouga S, Oliveira G: **Autism spectrum disorder: FRAXE mutation, a rare etiology**. *J Autism Dev Disord* 2015, **45**(3):888-892.

838. **SKYCLARYS (omaveloxolone) [package insert]. Plano, TX. Reata Pharmaceuticals, Inc.** 2023.

839. Irwin D, Lippa CF, Rosso A: **Effects of prescribed medications on cognition and behavior in frontotemporal lobar degeneration**. *Am J Alzheimers Dis Other Demen* 2010, **25**(7):566-571.

840. Tartaglia MC, Hu B, Mehta K, Neuhaus J, Yaffe K, Miller BL, Boxer A: **Demographic and neuropsychiatric factors associated with off-label medication use in frontotemporal dementia and Alzheimer's disease**. *Alzheimer Dis Assoc Disord* 2014, **28**(2):182-189.

841. Bei H, Ross L, Neuhaus J, Knopman D, Kramer J, Boeve B, Caselli RJ, Graff-Radford N, Mendez MF, Miller BL *et al*: **Off-label medication use in frontotemporal dementia**. *Am J Alzheimers Dis Other Demen* 2010, **25**(2):128-133.

842. Neylan KD, Miller BL: **New Approaches to the Treatment of Frontotemporal Dementia**. *Neurotherapeutics* 2023.

843. Gambogi LB, Guimaraes HC, de Souza LC, Caramelli P: **Treatment of the behavioral variant of frontotemporal dementia: a narrative review**. *Dement Neuropsychol* 2021, **15**(3):331-338.

844. Dodge S, Dacks P, Niehoff D, Dickinson S, Denny S, Graham L, Freeman B: **Frontotemporal Degeneration (FTD): A Voice of the Patient Report**. In*.*; 2021.

845. Wang RY, Bodamer OA, Watson MS, Wilcox WR: **Lysosomal storage diseases: Diagnostic confirmation and management of presymptomatic individuals**. *Genetics in Medicine* 2011, **13**(5):457-484.

846. Martensson J, Gustafsson J, Larsson A: **A therapeutic trial with N-acetylcysteine in subjects with hereditary glutathione synthetase deficiency (5-oxoprolinuria)**. *J Inherit Metab Dis* 1989, **12**(2):120-130.

847. Ristoff E, Mayatepek E, Larsson A: **Long-term clinical outcome in patients with glutathione synthetase deficiency**. *J Pediatr* 2001, **139**(1):79-84.

848. Boxer LA, Oliver JM, Spielberg SP, Allen JM, Schulman JD: **Protection of granulocytes by vitamin E in glutathione synthetase deficiency**. *N Engl J Med* 1979, **301**(17):901-905.

849. Milovanovic D, Djukic A, Stepanovic R, Pekovic D, Vranjesevic D: **[Hartnup disease (report of 2 cases in one family)]**. *Srp Arh Celok Lek* 2000, **128**(3-4):97-103.

850. Stoker TB, Mason SL, Greenland JC, Holden ST, Santini H, Barker RA: **Huntington's disease: diagnosis and management**. *Pract Neurol* 2022, **22**(1):32-41.

851. Laule C, Vavasour IM, Shahinfard E, Madler B, Zhang J, Li DKB, MacKay AL, Sirrs SM: **Hematopoietic Stem Cell Transplantation in Late-Onset Krabbe Disease: No Evidence of Worsening Demyelination and Axonal Loss 4 Years Post-allograft**. *J Neuroimaging* 2018, **28**(3):252-255.

852. McNeil-Gauthier AL, Brais B, Rouleau G, Anoja N, Ducharme S: **Successful treatment of psychosis in a patient with Kufor-Rakeb syndrome with low dose aripiprazole: a case report**. *Neurocase* 2019, **25**(3-4):133-137.

853. Pietrzak A, Badura-Stronka M, Kangas-Kontio T, Felczak P, Kozubski W, Latos-Bielenska A, Wierzba-Bobrowicz T, Florczak-Wyspianska J: **Clinical and ultrastructural findings in an ataxic variant of Kufor-Rakeb syndrome**. *Folia Neuropathol* 2019, **57**(3):285-294.

854. Pondrelli F, Muccioli L, Licchetta L, Mostacci B, Zenesini C, Tinuper P, Vignatelli L, Bisulli F: **Natural history of Lafora disease: a prognostic systematic review and individual participant data meta-analysis**. *Orphanet J Rare Dis* 2021, **16**(1):362.

855. Rodriguez A, Klein CJ, Sechi E, Alden E, Basso MR, Pudumjee S, Pittock SJ, McKeon A, Britton JW, Lopez-Chiriboga AS *et al*: **LGI1 antibody encephalitis: acute treatment comparisons and outcome**. *J Neurol Neurosurg Psychiatry* 2022, **93**(3):309-315.

856. Porpiglia F, Guillaume M, Bliaux E, Psimaras D, Decazes P, Guillin O, Rotharmel M, Morin A: **Anti-leucine-rich glioma-inactivated 1 encephalitis revealed by a manic episode: insights from frontal lobe dysfunction in neuropsychiatry through neuropsychology and metabolic imaging. A case report**. *Front Psychiatry* 2023, **14**:1168302.

857. Sundal C, Wszolek ZK: **CSF1R-Related Adult-Onset Leukoencephalopathy with Axonal Spheroids and Pigmented Glia**. In: *GeneReviews((R)).* Edited by Adam MP, Mirzaa GM, Pagon RA, Wallace SE, Bean LJH, Gripp KW, Amemiya A. Seattle (WA); 1993.

858. Doyle JJ, Doyle AJ, Wilson NK, Habashi JP, Bedja D, Whitworth RE, Lindsay ME, Schoenhoff F, Myers L, Huso N *et al*: **A deleterious gene-by-environment interaction imposed by calcium channel blockers in Marfan syndrome**. *Elife* 2015, **4**.

859. Jung HH, Danek A, Walker RH, Frey BM, Peikert K: **McLeod Neuroacanthocytosis Syndrome**. In: *GeneReviews((R)).* Edited by Adam MP, Mirzaa GM, Pagon RA, Wallace SE, Bean LJH, Gripp KW, Amemiya A. Seattle (WA); 1993.

860. Vazquez MJ, Martinez MC: **Electroconvulsive therapy in neuroacanthocytosis or McLeod syndrome**. *J ECT* 2009, **25**(1):72-73.

861. Santa KM: **Treatment options for mitochondrial myopathy, encephalopathy, lactic acidosis, and stroke-like episodes (MELAS) syndrome**. *Pharmacotherapy* 2010, **30**(11):1179-1196.

862. Solders M, Martin DA, Andersson C, Remberger M, Andersson T, Ringden O, Solders G: **Hematopoietic SCT: a useful treatment for late metachromatic leukodystrophy**. *Bone Marrow Transplant* 2014, **49**(8):1046-1051.

863. Benussi A, Cantoni V, Manes M, Libri I, Dell'Era V, Datta A, Thomas C, Ferrari C, Di Fonzo A, Fancellu R *et al*: **Motor and cognitive outcomes of cerebello-spinal stimulation in neurodegenerative ataxia**. *Brain* 2021, **144**(8):2310-2321.

864. Ng YS, Bindoff LA, Gorman GS, Horvath R, Klopstock T, Mancuso M, Martikainen MH, McFarland R, Nesbitt V, Pitceathly RDS *et al*: **Consensus-based statements for the management of mitochondrial stroke-like episodes**. *Wellcome Open Res* 2019, **4**:201.

865. Gruosso F, Montano V, Simoncini C, Siciliano G, Mancuso M: **Therapeutical Management and Drug Safety in Mitochondrial Diseases-Update 2020**. *J Clin Med* 2020, **10**(1).

866. Li SY, Lei HS, Wu XY, Li K, Liu ZM, Lu JH, Chen XY: **Minor allele of rs55763075 located in MTHFR is associated with the risk of cognitive impairment after anesthesia via modulating miR-34b**. *Sci Rep* 2021, **11**(1):11157.

867. Gomes JDA, Olstad EW, Kowalski TW, Gervin K, Vianna FSL, Schuler-Faccini L, Nordeng HME: **Genetic Susceptibility to Drug Teratogenicity: A Systematic Literature Review**. *Front Genet* 2021, **12**:645555.

868. Kalkan Ucar S, Ozbaran B, Demiral N, Yuncu Z, Erermis S, Coker M: **Clinical overview of children with mucopolysaccharidosis type III A and effect of Risperidone treatment on children and their mothers psychological status**. *Brain Dev* 2010, **32**(2):156-161.

869. Muschol N, Giugliani R, Jones SA, Muenzer J, Smith NJC, Whitley CB, Donnell M, Drake E, Elvidge K, Melton L *et al*: **Sanfilippo syndrome: consensus guidelines for clinical care**. *Orphanet J Rare Dis* 2022, **17**(1):391.

870. Lin A, Starr L, Schimmenti LA: **Myhre Syndrome Patient and Family Handbook**. In*.*, vol. 2; 2022.

871. Chandarana M, Saraf U, Divya KP, Krishnan S, Kishore A: **Myoclonus- A Review**. *Ann Indian Acad Neurol* 2021, **24**(3):327-338.

872. Verloes A, Maquet P, Sadzot B, Vivario M, Thiry A, Franck G: **Nasu-Hakola syndrome: polycystic lipomembranous osteodysplasia with sclerosing leucoencephalopathy and presenile dementia**. *J Med Genet* 1997, **34**(9):753-757.

873. Augustine E, Mink J: **Neuronal ceroid lipofuscinosis**. UpToDate, Waltham, MA. (Accessed on July 1, 2023.): UpToDate; 2022.

874. Lewis G, Morrill AM, Conway-Allen SL, Kim B: **Review of Cerliponase Alfa: Recombinant Human Enzyme Replacement Therapy for Late-Infantile Neuronal Ceroid Lipofuscinosis Type 2**. *J Child Neurol* 2020, **35**(5):348-353.

875. Keam SJ: **Arimoclomol: First Approval**. *Drugs* 2025, **85**(1):111-116.

876. Shelkowitz E, Saneto RP, Al-Hertani W, Lubout CMA, Stence NV, Brown MS, Long P, Walleigh D, Nelson JA, Perez FE *et al*: **Ketogenic diet as a glycine lowering therapy in nonketotic hyperglycinemia and impact on brain glycine levels**. *Orphanet J Rare Dis* 2022, **17**(1):423.

877. Van Hove JL, Vande Kerckhove K, Hennermann JB, Mahieu V, Declercq P, Mertens S, De Becker M, Kishnani PS, Jaeken J: **Benzoate treatment and the glycine index in nonketotic hyperglycinaemia**. *J Inherit Metab Dis* 2005, **28**(5):651-663.

878. Dhamija R, Gavrilova RH, Wirrell EC: **Valproate-induced worsening of seizures: clue to underlying diagnosis**. *J Child Neurol* 2011, **26**(10):1319-1321.

879. Rubenstein JL, Johnston K, Elliott GR, Brusilow SW: **Haloperidol-induced hyperammonaemia in a child with citrullinaemia**. *J Inherit Metab Dis* 1990, **13**(5):754-755.

880. Kazmierski D, Sharma N, O'Leary K, Ochieng P: **Valproate-induced fatal acute hyperammonaemia-related encephalopathy in late-onset ornithine transcarbamylase deficiency**. *BMJ Case Rep* 2021, **14**(5).

881. Bigot A, Brunault P, Lavigne C, Feillet F, Odent S, Kaphan E, Thauvin C, Leguy V, Broue P, Tchan MC *et al*: **Psychiatric adult-onset of urea cycle disorders: A case-series**. *Mol Genet Metab Rep* 2017, **12**:103-109.

882. Gomber S, Saxena R, Madan N: **Comparative efficacy of desferrioxamine, deferiprone and in combination on iron chelation in thalassemic children**. *Indian Pediatr* 2004, **41**(1):21-27.

883. San Jose Caceres A, Landlust AM, Carbin JM, European Phelan-McDermid Syndrome c, Loth E: **Consensus recommendations on sleeping problems in Phelan-McDermid syndrome**. *Eur J Med Genet* 2023, **66**(6):104750.

884. Phelan K, Rogers RC, Boccuto L: **Phelan-McDermid Syndrome**. In: *GeneReviews((R)).* Edited by Adam MP, Mirzaa GM, Pagon RA, Wallace SE, Bean LJH, Gripp KW, Amemiya A. Seattle (WA); 1993.

885. Kolevzon A, Bennett J, W., Berry-Kravis EM, Neumeyer A, Rogers C: **Therapeutic interventions in Phelan-McDermid syndrome**. In*.* Phelan-McDermid Syndrome Foundation; 2021.

886. Kolevzon A, Bennett J, W., Berry-Kravis EM, Neumeyer A, Rogers C, Kohlenberg T: **Sensitivity to Psychiatric Medications in Phelan-McDermid Syndrome**. In*.* Phelan-McDermid Syndrome Foundation; 2021: 1.

887. Srivastava S, Sahin M, Buxbaum JD, Berry-Kravis E, Soorya LV, Thurm A, Bernstein JA, Asante-Otoo A, Bennett WE, Jr., Betancur C *et al*: **Updated consensus guidelines on the management of Phelan-McDermid syndrome**. *Am J Med Genet A* 2023.

888. Kaur S, Christodoulou J: **MECP2 Disorders**. In: *GeneReviews((R)).* Edited by Adam MP, Mirzaa GM, Pagon RA, Wallace SE, Bean LJH, Gripp KW, Amemiya A. Seattle (WA); 1993.

889. Sarathi V, Wadhwa R: **Albright Hereditary Osteodystrophy**. In: *StatPearls.* Treasure Island (FL); 2023.

890. Fu C, Armstrong D, Marsh E, Lieberman D, Motil K, Witt R, Standridge S, Nues P, Lane J, Dinkel T *et al*: **Consensus guidelines on managing Rett syndrome across the lifespan**. *BMJ Paediatr Open* 2020, **4**(1):e000717.

891. Fernandez-Ramos JA, De la Torre-Aguilar MJ, Quintans B, Perez-Navero JL, Beyer K, Lopez-Laso E, Spanish Segawa Disease Research g: **Genetic landscape of Segawa disease in Spain. Long-term treatment outcomes**. *Parkinsonism Relat Disord* 2022, **94**:67-78.

892. Polymeropoulos CM, Brooks J, Czeisler EL, Fisher MA, Gibson MM, Kite K, Smieszek SP, Xiao C, Elsea SH, Birznieks G *et al*: **Tasimelteon safely and effectively improves sleep in Smith-Magenis syndrome: a double-blind randomized trial followed by an open-label extension**. *Genet Med* 2021, **23**(12):2426-2432.

893. Sullivan R, Yau WY, O'Connor E, Houlden H: **Spinocerebellar ataxia: an update**. *J Neurol* 2019, **266**(2):533-544.

894. Ostrowski PJ, Tatton-Brown K: **Tatton-Brown-Rahman Syndrome**. In: *GeneReviews((R)).* Edited by Adam MP, Mirzaa GM, Pagon RA, Wallace SE, Bean LJH, Gripp KW, Amemiya A. Seattle (WA); 1993.

895. Xiao C, Tifft C, Toro C: **Sandhoff Disease**. In: *GeneReviews((R)).* Edited by Adam MP, Mirzaa GM, Pagon RA, Wallace SE, Bean LJH, Gripp KW, Amemiya A. Seattle (WA); 1993.

896. Northrup H, Aronow ME, Bebin EM, Bissler J, Darling TN, de Vries PJ, Frost MD, Fuchs Z, Gosnell ES, Gupta N *et al*: **Updated International Tuberous Sclerosis Complex Diagnostic Criteria and Surveillance and Management Recommendations**. *Pediatr Neurol* 2021, **123**:50-66.

897. van der Knaap MS, Bonkowsky JL, Vanderver A, Schiffmann R, Krageloh-Mann I, Bertini E, Bernard G, Fatemi SA, Wolf NI, Saunier-Vivar E *et al*: **Therapy Trial Design in Vanishing White Matter: An Expert Consortium Opinion**. *Neurol Genet* 2022, **8**(2):e657.

898. Albanese A, Romito LM, Calandrella D: **Therapeutic advances in dystonia**. *Mov Disord* 2015, **30**(11):1547-1556.

899. Bohlega SA, Abusrair A: **Woodhouse-Sakati Syndrome**. In: *GeneReviews((R)).* Edited by Adam MP, Mirzaa GM, Pagon RA, Wallace SE, Bean LJH, Gripp KW, Amemiya A. Seattle (WA); 1993.
